# Asymmetric Sulfhydryl-Substituted Squaraine Fluorophores for Sustained SIM Imaging in Live Cells

**DOI:** 10.1021/jacs.6c06633

**Published:** 2026-08-27

**Authors:** Yibo Zhou, Pin Shang, Huiqiu Shi, Jun Zhang, Fan Li, Zhihe Qing, Tony D. James

**Affiliations:** † Hunan Provincial Key Laboratory of Cytochemistry, School of Chemistry and Pharmaceutical Engineering, 12418 Changsha University of Science and Technology, Changsha 410114, China; ‡ Department of Chemistry, 1555 University of Bath, Bath BA7AY, U.K.; § School of Chemistry and Chemical Engineering, Henan Normal University, Xinxiang 453007, China

## Abstract

Recognized for its excellent temporal and spatial resolution, structured illumination microscopy (SIM) requires photostable fluorophores to enable dynamic imaging. Squaraine fluorophores are promising candidates for fluorescence imaging because of their intriguing photophysical properties. However, traditional squaraine fluorophores, based on an oxocyclobutenolate ring with a symmetric structure, suffer from poor photostability, limiting their application in SIM super-resolution imaging, especially for sustained tracking of biological events. To address this issue, we developed a strategy for improving the photostability of squaraine by synthesizing asymmetric molecular antennae and substituting the hydroxyl with sulfhydryl. The enhancement of intramolecular charge transfer (ICT) in the asymmetric structure enhances resistance to photobleaching, and the thiolation prolongs the fluorescence lifetime, resulting in a highly stable squaraine with remarkable optical performance. As such, we used our asymmetric thio-squaraine for tracking the dynamic changes of mitochondria during interactions between mitochondria and lysosomes. Significantly, our design strategy enables the synthesis of various thio-squaraine derivatives with superior photostability, long emission wavelengths, and high brightness, broadening the potential applicability of squaraine fluorophores in the field of SIM imaging.

## Graphical abstract

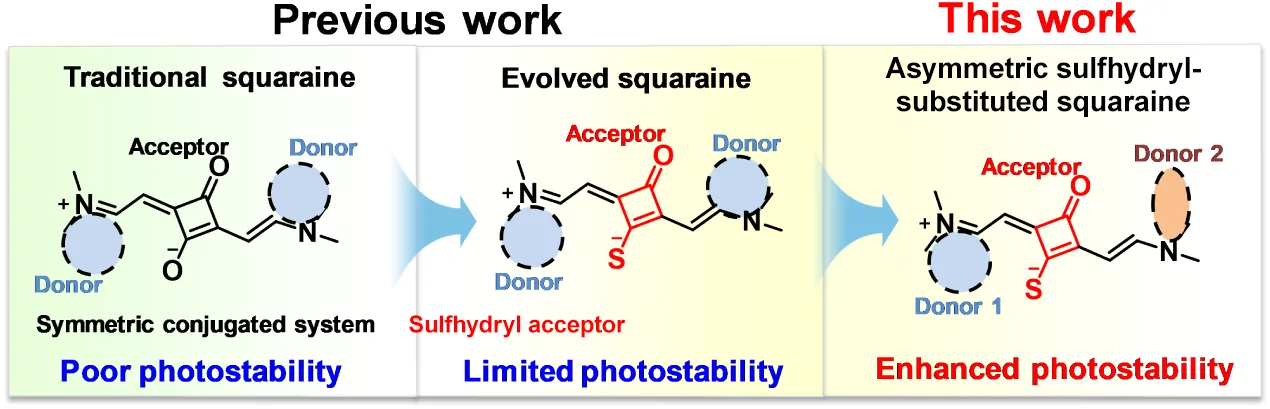

## Introduction

Super-resolution microscopy has been extensively used to acquire information about cellular submicrostructure due to its exceptionally high resolution (<200 nm).^1,2^ Among various super-resolution microscopic methods, structured illumination microscopy (SIM) exhibits several advantages compared with other optical microscopic techniques.^3^ For instance, stochastic optical reconstruction microscopy (STORM)^4^ and photoactivation localization microscopy (PALM)^5^ realize high spatial resolution by sacrificing temporal resolution, and stimulated emission depletion microscopy (STED)^6^ uses high-power depletion lasers that can cause fluorescence quenching. In contrast, with improved imaging speed, SIM achieves an optimal balance between temporal and spatial resolution.^7,8^ However, excessive light exposure can affect the performance of fluorescence imaging, requiring improved stability of the fluorophore in order to prevent photobleaching.^9,10^ Thus, developing a fluorophore with superior photostability is crucial for SIM imaging, especially during prolonged imaging, which requires continuous illumination.

Squaraine fluorophores are based on oxocyclobutenolate rings, and have been widely used for fluorescence imaging due to their near-infrared emission and high fluorescence quantum yields.^11–13^ Nevertheless, the oxocyclobutenolate core of squaraine is photolytically labile,^14^ resulting in reduced stability of squaraine and limiting its practical utility. To alleviate this problem, several strategies focused on the central cyclobutene have been developed for improving the photostability of squaraine. An early solution was to use the formation of an internal salt bridge to protect the central oxocyclobutenolate ring by introducing quaternary ammonium-modified alkanes.^15,16^ Another method requires replacement of oxhydryl with sulfhydryl; sulfur’s stronger electronegativity enhances 3p orbital delocalization, which prolongs the fluorescence lifetime of squaraine by suppressing vibrational nonradiative decay pathways (Figure 1).^17,18^ However, despite these significant improvements in photostability using ring protection or lifetime extension, the impact of the molecular framework itself has been largely neglected.

**Figure 1:**
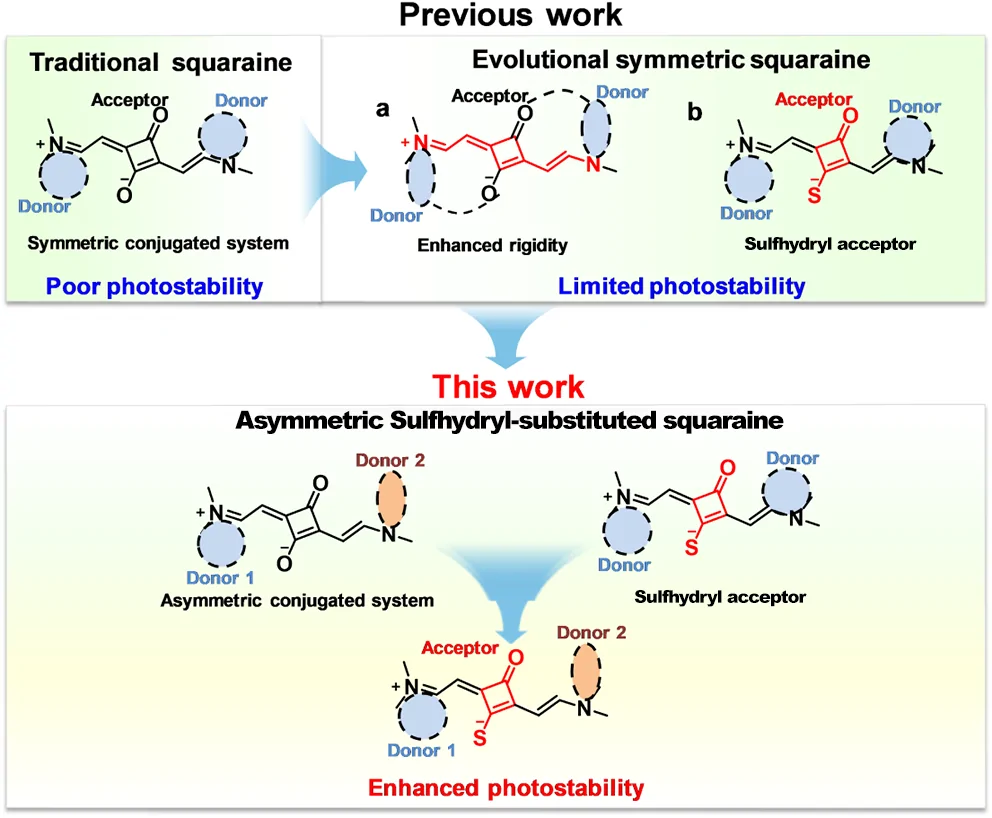
Design of traditional symmetric squaraine with limited photostability (above) and asymmetric sulfhydryl-substituted squaraine with excellent photostability prepared as part of this research (below).

Generally, squaraines are constructed using symmetric electron-rich aromatic or heterocyclic compounds with electron-deficient squaric acid, such that electron transfer in the donor–acceptor–donor (D-A-D) molecular structure generates fluorescence.^19,20^ Unfortunately, the fluorescence rapidly decreases after several excitation–emission cycles due to photobleaching of the symmetric fluorophores.^21^ As such, we considered how to use molecular engineering strategies to tailor the molecular structure of squaraine in order to achieve enhanced photostability.

Herein, to circumvent the stability limitations of squaraine, we developed a strategy using asymmetric molecular antennae and sulfhydryl substitution of the oxocyclobutenolate ring to enable enhanced photostability and extended fluorescence lifetime (Figure 1). Specifically, the strong intramolecular charge transfer (ICT) effect induced by symmetry breaking lowers the excited-state energy and segregates electron density, thereby suppressing photooxidative degradation pathways to enhance photostability.^22^ Moreover, thiolation of the oxocyclobutenolate ring can prolong the fluorescence lifetime of the entire molecular system.^17^ To demonstrate the fluorescence performance of asymmetric thio-squaraines, we synthesized a mitochondria-targeted fluorescent tag by introducing an octadecyl chain, which was successfully used for high-resolution SIM fluorescence imaging of mitochondria in live cells. Compared with commercial mitochondria trackers, this fluorescent tag exhibited a prolonged fluorescence lifetime and revealed the interactions between mitochondria and lysosomes during mitophagy. As such, with this research, the application of squaraine fluorophores in the field of SIM imaging was significantly extended.

## Results and Discussion

### Design and Synthesis of Asymmetric Squaraine Derivatives

Using standard squaraines as a model system, we set out to explore the development of asymmetric squaraines by changing one of the two electron-rich groups. As shown in Figure 2A, a symmetric squaraine was prepared using two indole derivatives (2,3,3-trimethylindolenine) covalently linked to squaric acid to generate the control squaraine fluorophore (SF), which contains symmetric molecular antennae.^23^ We then prepared asymmetric squaraine derivatives using squaric acid as the scaffold, where one side of the molecule still contained an indole derivative, but the other side was replaced with a flavylium salt, a benzoindolenine derivative, and a benz­[cd]­indol derivative to obtain SF-F, SF-BI, and SF-BcI, respectively. From the perspective of molecular structure, the asymmetric electron cloud density in SF-F triggers an ICT effect, which serves as a protective mechanism against photodegradation by stabilizing the excited state. Driven by this consideration, SF-F was selected for further thionation to prepare the SF-F-S squaraine derivative to improve stability. The detailed synthetic routes of these dyes are described in Scheme S1, and the ^1^H and ^13^C NMR and ESI-MS results confirmed that the intermediate compounds and final dyes were successfully synthesized (Figures S1–S9).

**Figure 2:**
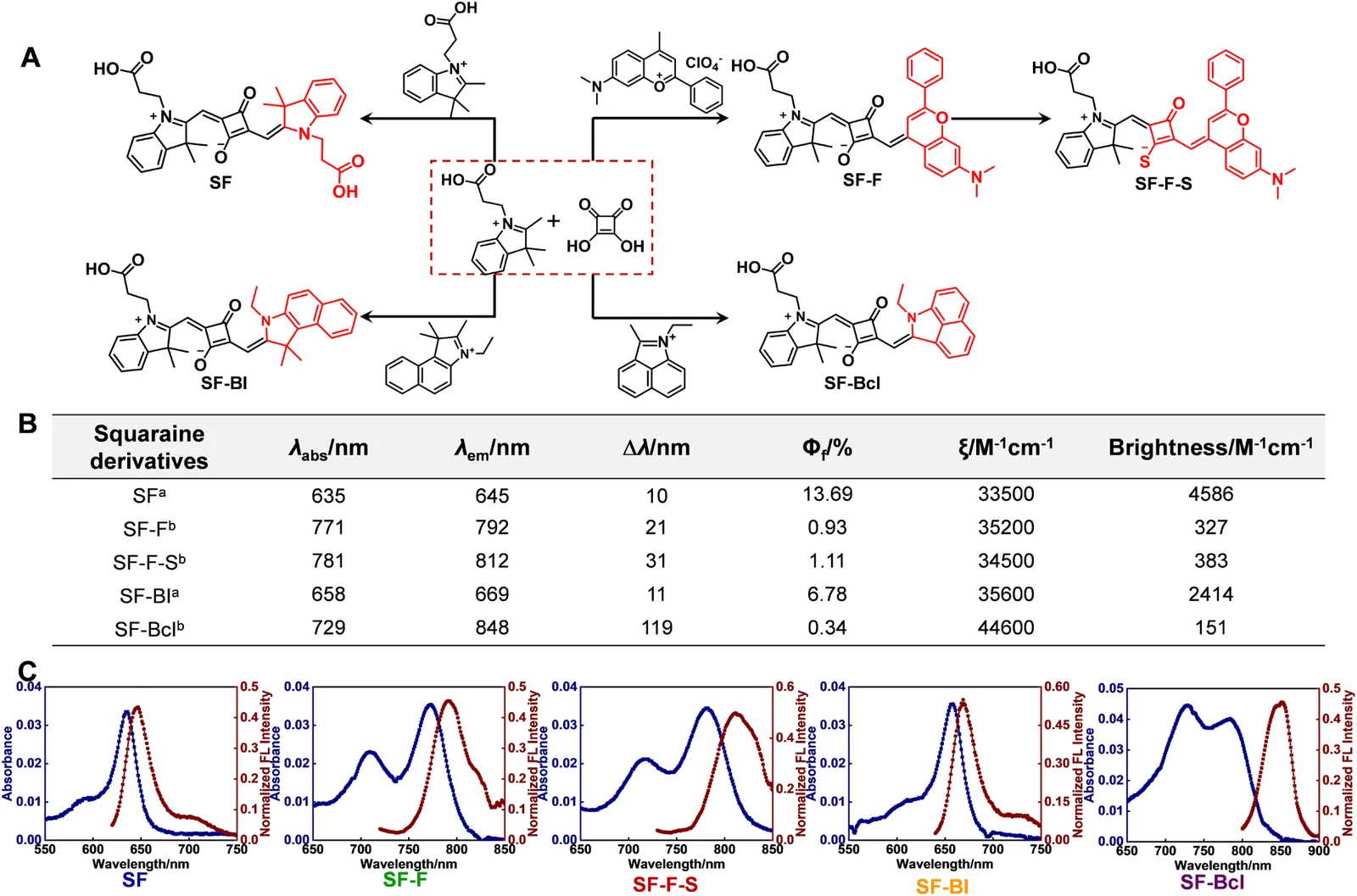
(A) Synthetic route for asymmetric squaraine derivatives. (B) Photophysical properties of the asymmetric squaraine derivatives in DMSO. a. Fluorescence quantum yields were calculated with cresyl violet as the reference (Φ_f_ = 0.54, MeOH). b. Fluorescence quantum yields were calculated with IR 780 as the reference (Φ_f_ = 0.17, EtOH). (C) UV–vis-NIR absorption spectra and normalized fluorescence emission spectra of the squaraine derivatives in DMSO.

### Photophysical Properties of Asymmetric Squaraine Derivatives

With these squaraine derivatives in hand, their photophysical properties were investigated. As shown in Figure 2B and Figure 2C, compared with the control SF (λ_abs_/λ_emo_ = 635/645 nm), apart for SF-BI, the absorption peak of the squaraine derivatives exhibited bathochromic shifts (>700 nm), with SF-F, SF–F-S and SF-BcI at 771, 781, and 729 nm, respectively. From the molecular structure, the conjugative effect of SF-BI was similar to SF, but the other squaraine derivatives contained larger π-conjugated systems, and thus the observed absorptions were red-shifted.^24,25^ Correspondingly, the fluorescence emission peaks of SF-F and SF–F-S appeared at around 800 nm, which exceeded that of SF (645 nm). While the emission of SF-BcI achieved 848 nm due to the strong electronic push–pull effect between the indole derivative and benz­[cd]­indol. Notably, the asymmetric molecular structures (SF-F, SF–F-S, and SF-BcI) exhibited larger Stokes shifts than SF, which was attributed to enhanced ICT.^25,26^ Taking SF-F as an example, the flavylium has superior electron-donating ability when compared to the indole in SF, which not only facilitates molecular polarization but also significantly enhances the ICT effect;^27,28^ thus, larger Stokes shifts and red-shifted emissions were generated in these asymmetric squaraine derivatives.

All the squaraine derivatives exhibited high fluorescence quantum yields. For SF, the quantum yield was nearly 14% in DMSO (Figure 2B). For SF-BI, its quantum yield is comparable to that of SF in various solvents, including EtOH, ACN, and MeOH (Figure S10, Table S1). Compared with SF, the reduced quantum yield of asymmetric SF-F and SF–F-S arises from the introduction of strong ICT character, which decreases the radiative decay rate while simultaneously promoting nonradiative decay.^17^ Notably, for SF–F-S, the incorporation of sulfur (S) atoms increases the nonradiative decay rate through enhanced vibrational deactivation and a reduced energy gap, further decreasing the quantum yield. However, for bioimaging applications, the resulting large Stokes shift and superior photostability are of greater significance and are highly desirable. All the asymmetric squaraine derivatives exhibited large molar extinction coefficients (>10^4^ M^–1^ cm^–1^), which contribute to the exceptional optical brightness. These results indicate that these asymmetric squaraine derivatives not only exhibit conventional photophysical properties comparable to SF but also exhibit larger Stokes shifts and red-shifted fluorescence excitation and emission signals, making them more suitable for biological applications. Given the similar molecular structures, SF, SF-F, and SF–F-S were selected to investigate photostability in the following sections.

### Theoretical Calculations

In order to better understand the potential relationship between molecular structure and the photophysical properties of squaraine derivatives, the electronic orbitals of SF, SF-F, and SF–F-S were calculated by density functional theory (DFT). For SF, the highest occupied molecular orbital (HOMO, −4.885 eV) and the lowest unoccupied molecular orbital (LUMO, −2.589 eV) are shown in Figure 3A. For SF-F (HOMO, −4.750 eV; LUMO, −2.786 eV) and for SF–F-S (HOMO, −4.736 eV; LUMO, −2.806 eV), the corresponding orbitals are shown in Figure 3A. As such, the electronic gap of SF was 2.296 eV, much larger than that of SF-F (1.964 eV) and SF–F-S (1.929 eV), which helps explain the observed photophysical properties. The electron density distribution map indicated that the electrons of SF are equally distributed throughout the backbone of the molecule; thus, the “cyanine limit” observed for cyanine dyes and originating from the molecular symmetry can be observed for SF,^29,30^ resulting in limited Stokes shifts (Δλ = 10 nm). In contrast, with SF-F and SF–F-S, the asymmetric substitution with a flavylium group breaks the balance of electron densities, and the electron cloud mainly concentrates on the flavylium group (LUMO), effectively decreasing the LUMO energy level to reduce the energy gap, indicating that that the asymmetric structure here is beneficial for overcoming the “cyanine limit.” ^31,32^ For the optimized structures of squaraine derivatives in different states (Figure 3B), the root-mean-square deviation (RMSD) of SF–F-S is 0.071 Å, which is much larger than that of SF-F (0.067 Å) and SF (0.056 Å),^33^ indicating that SF–F-S undergoes pronounced geometric relaxation, which enhances the Stokes shift.

**Figure 3:**
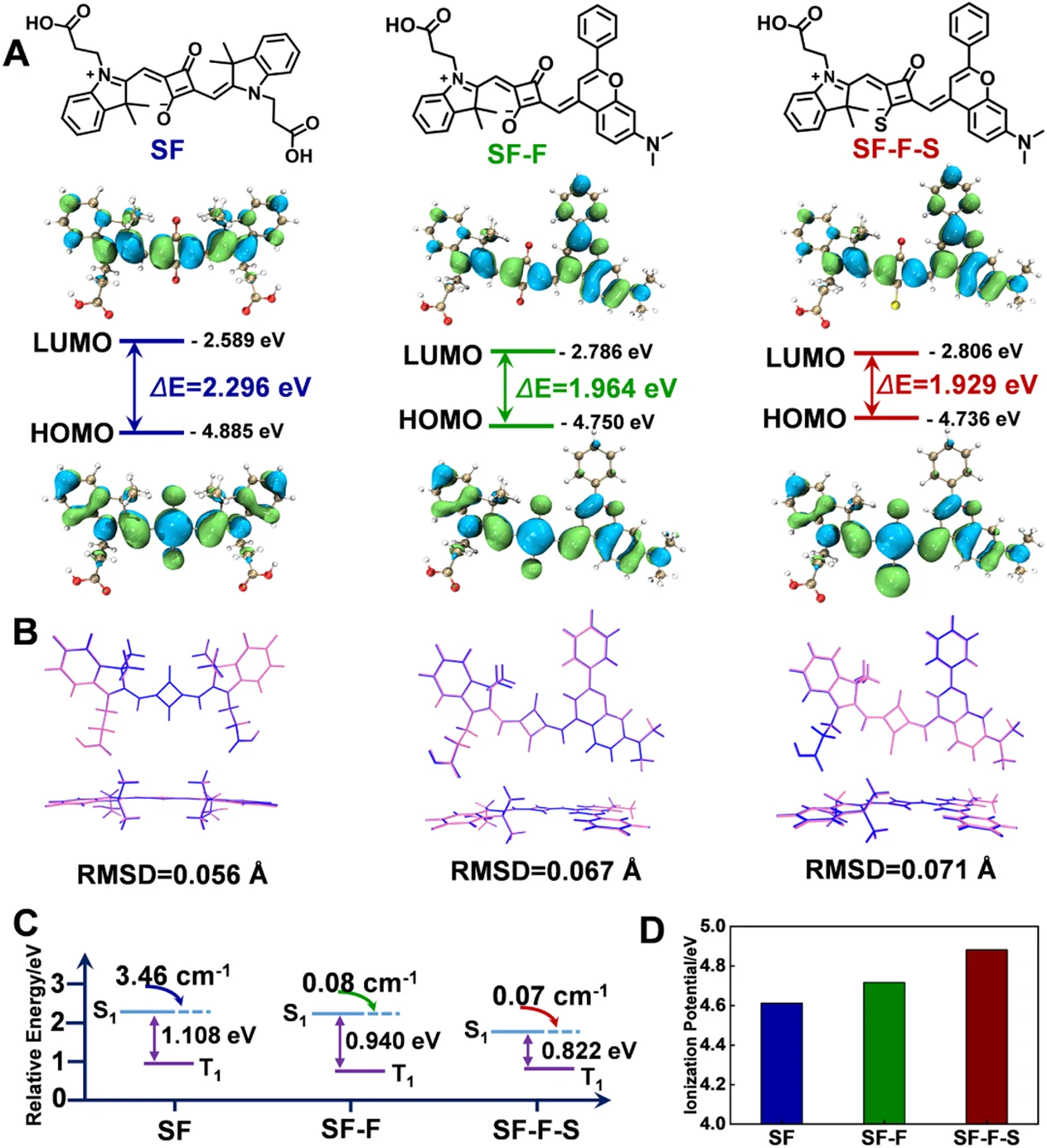
(A) Molecular structure and DFT-optimized molecular orbital diagrams (LUMO and HOMO) of SF, SF-F, and SF–F-S, and the quantized energy gap between the LUMO and HOMO. (B) Optimized structures of SF, SF-F, and SF–F-S, and the RMSD of different molecular structures during excited-state geometry relaxation. (C) Calculated spin–orbit coupling (SOC) and Δ*E*_ST_ of SF, SF-F, and SF–F-S at their S_1_ state. (D) Ionization potentials of SF, SF-F, and SF–F-S at the T_1_ state, respectively.

Since it is important to verify the photostability of these synthetic fluorophores, we calculated related molecular parameters to help predict the potential mechanism of resistance to photobleaching. As shown in Figure 3C, the singlet (S_1_) and triplet (T_1_) states of squaraine fluorophores were determined. The spin–orbit coupling (SOC) of SF was 3.46 cm^–1^, much larger than that of SF-F (0.08 cm^–1^) and SF–F-S (0.07 cm^–1^). Although the energy gap (Δ*E*_ST_) decreases from SF (1.108 eV) to SF–F-S (0.822 eV), which would thermodynamically favor intersystem crossing (ISC), the dramatic reduction in SOC magnitude from SF to SF–F-S plays a more dominant role in suppressing the ISC.^34,35^ Given that photochemical reactions often occur when in the reactive T_1_ state but not the S_1_ state,^36^ the limited ISC of SF–F-S provides superior photostability. Notably, despite the incorporation of sulfur, the enhanced ICT character in SF–F-S induces pronounced orbital decoupling that outweighs the heavy-atom effect, resulting in a reduced SOC compared to SF-F. Meanwhile, the ionization potentials of the three fluorophores were determined in order to evaluate their susceptibility to photooxidation. As shown in Figure 3D, the ionization potentials of SF, SF-F, and SF–F-S were 4.63, 4.72, and 4.82 eV, respectively. The higher ionization potentials of SF-F and SF–F-S suggested that the asymmetric squaraine fluorophores exhibit enhanced resistance toward photooxidation. Taken together, these theoretical results indicate strong oxidation resistance and a reduced probability of T_1_ state formation for SF-F and SF–F-S. As such, these asymmetric squaraine fluorophores are expected to exhibit excellent photostability during fluorescence imaging applications when compared with SF.

### Photostability of Squaraine Derivatives

Subsequently, we examined the photostability of the squaraine derivatives in vitro. First, to ensure that all samples absorbed an identical number of photons for the evaluation of their photostability, 660 nm was selected as the excitation wavelength because the absorbance remained constant across the three dye samples at the same concentration. Then, the absorption spectra of the squaraine derivatives were recorded at different powers (Figure S11). The absorption peak at 639 nm of SF gradually decreased with increasing laser power, but the absorption spectra of SF-F and SF–F-S changed only slightly under the same conditions. When the laser power was set to 83 mW, the difference between the three fluorophores could be clearly observed; thus, we chose 83 mW laser illumination for the following experiments. The absorption spectra of squaraine derivatives were monitored with an increase of illumination time. As shown in Figure 4A, the absorption value of SF reduced to 33.2% after 210 min, but that of SF-F only exhibited a slight decrease in the absorption peak (Figure 4B). In contrast, the SF–F-S remained stable throughout the whole illumination period (Figure 4D, 95.5%), demonstrating the favorable photostability of the asymmetric squaraine. Meanwhile, the fluorescence emission of the three dyes was also recorded under the same conditions. As depicted in the absorption spectra, a significant decrease of fluorescence intensity was observed for SF (Figure 4E) along with SF-F (Figure 4F). Interestingly, the fluorescence intensity of SF–F-S exhibited an increase at the start of illumination and then gradually decreased with increasing illumination time (Figure 4G). From the quantized fluorescence ratio (Figure 4H), the fluorescence intensity of SF–F-S remained basically unchanged until 190 min, and we believe that this change is due to the photoconversion of SF–F-S to SF-F, since, when SF–F-S is illuminated with a laser, the sulfhydryl of SF–F-S can be transformed into a hydroxyl,^17^ eventually forming SF-F. To further confirm this result, fluorescence decay curve measurements of the three dyes were performed. As shown in Figure S12, the decay curve of SF exhibited a single-exponential pattern with a lifetime of 1.08 ns, but the lifetimes of SF-F and SF–F-S were 2.04 and 2.78 ns, respectively, verifying the previous conclusion. Moreover, both of the squaraine dyes were stable under dark conditions (Figure S13), proving that the reduction of fluorescence intensity originates from photobleaching. Simultaneously, we examined the photostability of SF-BI and SF-BcI under the same conditions (Figure S14). Much like SF, the absorbance and fluorescence of SF-BI dropped to 39.2% and 41% after exposure to light, while SF-BcI exhibited 80.4% and 76.8% retention, respectively. These findings confirm that SF–F-S remains the most stable among the five SF derivatives. To further verify the enhancement of photostability by the asymmetric structure and thionation, we synthesized the sulfhydryl-substituted squaraine SF-S as a control (Figure S15). Spectroscopic experiments demonstrated that both the absorption and fluorescence intensities of SF-S gradually decreased under continuous light irradiation (Figure S16), which demonstrates that enhanced photostability arises from the cooperative interplay between ICT-induced orbital separation and sulfur-mediated electronic modulation,^37^ rather than the simple addition of two independent effects.

**Figure 4:**
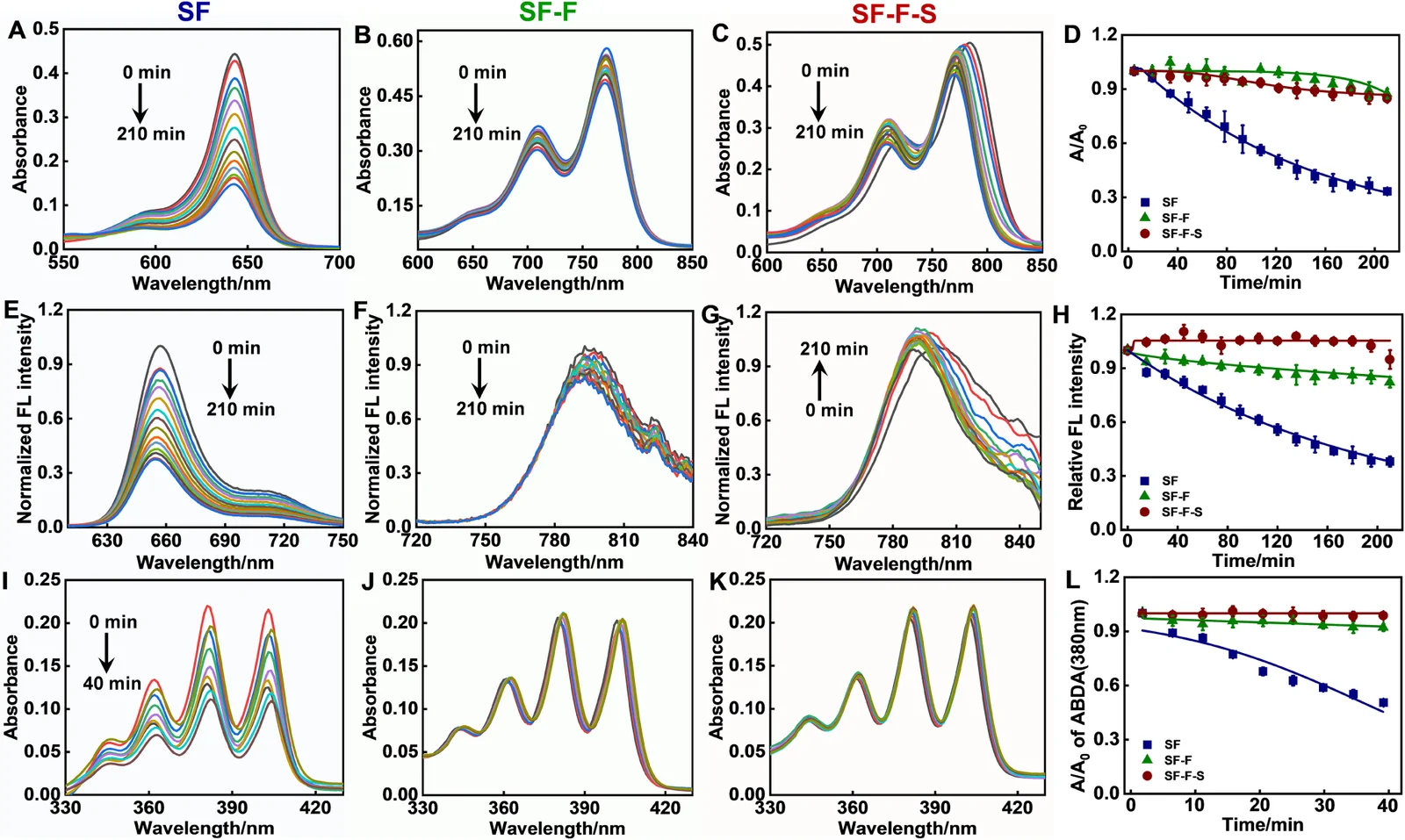
UV–vis-NIR absorption spectra of (A) SF (5 μM), (B) SF-F (5 μM), and (C) SF–F-S (5 μM) in trifluoroethanol under laser illumination (83 mW) at different time points (0, 15, 30, 45, 60, 75, 90, 105, 120, 135, 150, 165, 180, 195, and 210 min). (D) Changes of relative absorption for the three squaraine derivatives at different time points. Fluorescence emission spectra of (E) SF (5 μM), (F) SF-F (5 μM), and (G) SF–F-S (5 μM) in trifluoroethanol under laser illumination (83 mW) at different time points (0, 15, 30, 45, 60, 75, 90, 105, 120, 135, 150, 165, 180, 195, and 210 min). (H) Fluorescence ratio changes of squaraine derivatives at different time points. UV–vis absorption spectrum of ABDA (15 μM) containing (I) SF (5 μM), (J) SF-F (5 μM), and (K) SF–F-S (5 μM) under laser illumination (83 mW) at different time points (0, 5, 10, 15, 20, 25, 30, 35, and 40 min). (L) Relative changes of absorbance at 380 nm of ABDA containing squaraine derivatives under laser illumination. Error bars were calculated from three repeated measurements.

One reason for the photobleaching of the fluorophore is that the triplet state of the fluorophore can react with oxygen to generate singlet oxygen,^38^ which will cause fluorophore bleaching and cytotoxicity. To investigate the triplet-state formation of the squaraine derivatives, the singlet oxygen generated by these dyes was monitored. As shown in Figure 4I, using 9,10-anthracenediyl-bis­(methylene)-dimalonic acid (ABDA) as an indicator,^39^ the absorption spectra of ABDA decreased significantly when a solution containing SF was exposed to laser illumination. Considering that the intensity of ABDA remains stable in the absence of SF under the same conditions (Figure S17), we conclude that SF generates singlet oxygen, causing photobleaching. However, ABDA was only slightly affected by SF-F (Figure 4J), and almost no response was observed with SF–F-S (Figure 4K and 4L), demonstrating excellent stability. These optical experimental results are in accordance with the theoretical predictions, providing a solid foundation for the application of these probes to fluorescence imaging.

### Photostability and Phototoxicity of Squaraine Derivatives in Live Cells

On the basis of the excellent performance of the asymmetric squaraine derivatives, we decided to evaluate fluorescence imaging in cells. However, prior to that, we evaluated the cytotoxicity. As shown in Figure S18, live hepatoma carcinoma cells (HepG2 cells) were incubated with various concentrations of SF, SF-F, and SF–F-S, and the cell viability was recorded. Significantly, the cell viability was maintained at over 83% even after being treated with the dyes for 24 h, demonstrating the good biocompatibility of the squaraine derivatives. Then, cells were incubated with dyes and imaged using confocal microscopy to investigate photostability. As shown in Figure S19, the fluorescence signal penetrated the whole cell (Z-scanning fluorescence images), confirming that all of the dyes entered the cells. Moreover, the cells were monitored for a long time to evaluate the imaging ability of the squaraine derivatives. As shown in Figure 5A, after being treated with the fluorophores for 30 min, the images were acquired using SIM. The fluorescence signal with SF reduced gradually, and the intensity was reduced to 26.4% after the acquisition of 60 images (at 40 min) (Figure 5D), illustrating that the fluorescence of SF was bleached under sustained laser excitation. However, the fluorescence intensity of SF-F decreased to just 80.5% after 110 min of irradiation (Figure 5B), and the SF–F-S remained stable during the entire process of imaging (0–110 min) (Figure 5C), which indicated that the asymmetric and sulfhydryl-functionalization strategy can effectively enhance the imaging ability of squaraine fluorophores.

**Figure 5:**
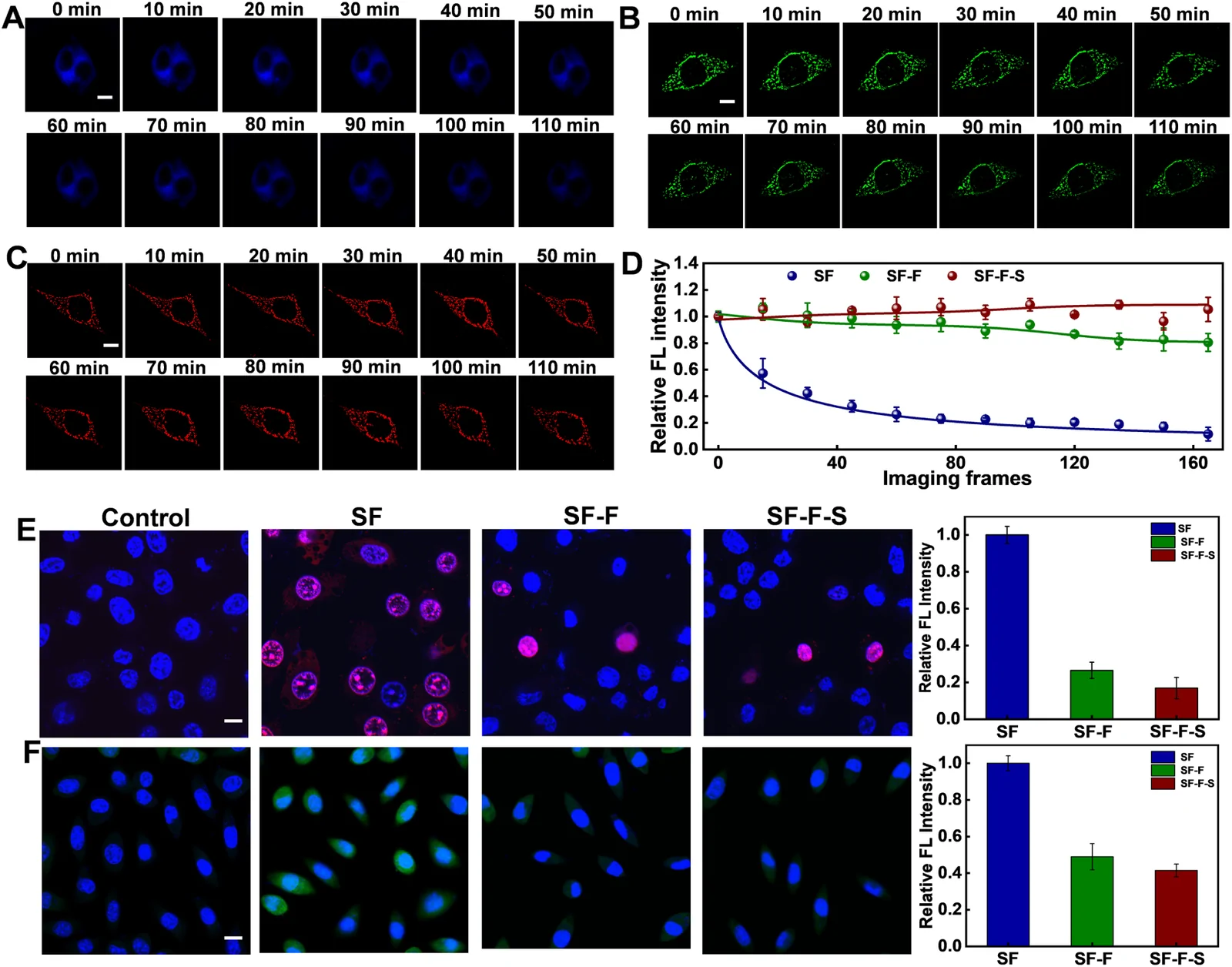
SIM images of live cells incubated with (A) SF (10 μM), (B) SF-F (10 μM), and (C) SF–F-S (10 μM) under laser illumination (50 W/cm^2^) at different time points. λ_ex_ = 640 nm, λ_em_ = 663–738 nm. Scale bars: 10 μm. (D) Changes of relative fluorescence intensity of live cells incubated with squaraine derivatives under continuous imaging mode. (E) Confocal fluorescence images of live cells incubated with squaraine derivatives and after being treated with Hoechst 33342 (5 μM, λ_ex_ = 405 nm, λ_em_ = 430–470 nm) and PI (5 μM, λ_ex_ = 561 nm, λ_em_ = 570–670 nm). The fluorescence intensity of live cells incubated with SF was defined as 1.0. Scale bar: 10 μm. (F) Confocal fluorescence images of live cells incubated with squaraine derivatives and with Hoechst 33342 (5 μM, λ_ex_ = 405 nm, λ_em_ = 430–470 nm) and DCFH-DA (5 μM, λ_ex_ = 488 nm, λ_em_ = 500–600 nm). The fluorescence intensity of live cells incubated with SF was defined as 1.0. Scale bar: 10 μm. Error bars were calculated from three repeated measurements.

In addition, the phototoxicity of the fluorescence signaling unit is an important aspect of imaging, especially during long-term fluorescence imaging. As such, live cells were incubated with the squaraine derivatives for 1 h and then treated with a cell viability indicator (propidium iodide, PI). As shown in Figure 5E, the number of apoptotic cells for SF–F-S was significantly less than that of SF-F and SF. Significantly, the apoptosis rate of cells incubated with SF and SF-F was 5.9- and 1.6-fold higher than that of SF–F-S, which may be attributed to the minimal formation of singlet oxygen with SF–F-S. To further confirm this hypothesis, a commercial ROS fluorescent probe (DCFH-DA) was coincubated with the live cells under the same conditions. From Figure 5F, we can see that the green fluorescence signal of DCFH-DA can be observed for cells treated with SF, but the signal is much weaker for SF-F. For SF–F-S, the signal intensity is 41% that of SF, indicating that only trace amounts of ROS are generated by SF–F-S, which is consistent with the in vitro result.

### Synthesis of Mitochondria-Targeted Tracker Using SF–F-S

Motivated by the high photostability and low phototoxicity of SF–F-S, we decided to prepare a mitochondria-targeted tracker using SF–F-S to confirm the practical applicability of the squaraine probe (Figure 6A). Live cells were incubated with SF–F-S, commercial mitochondrial, and lysosomal trackers to investigate the location of the free dyes. As shown in Figure S20, the green fluorescence signal of the commercial trackers and the red fluorescence signal of SF–F-S can be found in the cells, and the merged images revealed that the red and green colors exhibited a low Pearson’s colocalization coefficient of 0.24, suggesting that SF–F-S does not localize in the mitochondria or lysosomes. And this phenomenon was also observed for SF (Figure S21) and SF-F (Figure S22), which implies that these squaraine derivatives do not target specific organelles.

**Figure 6:**
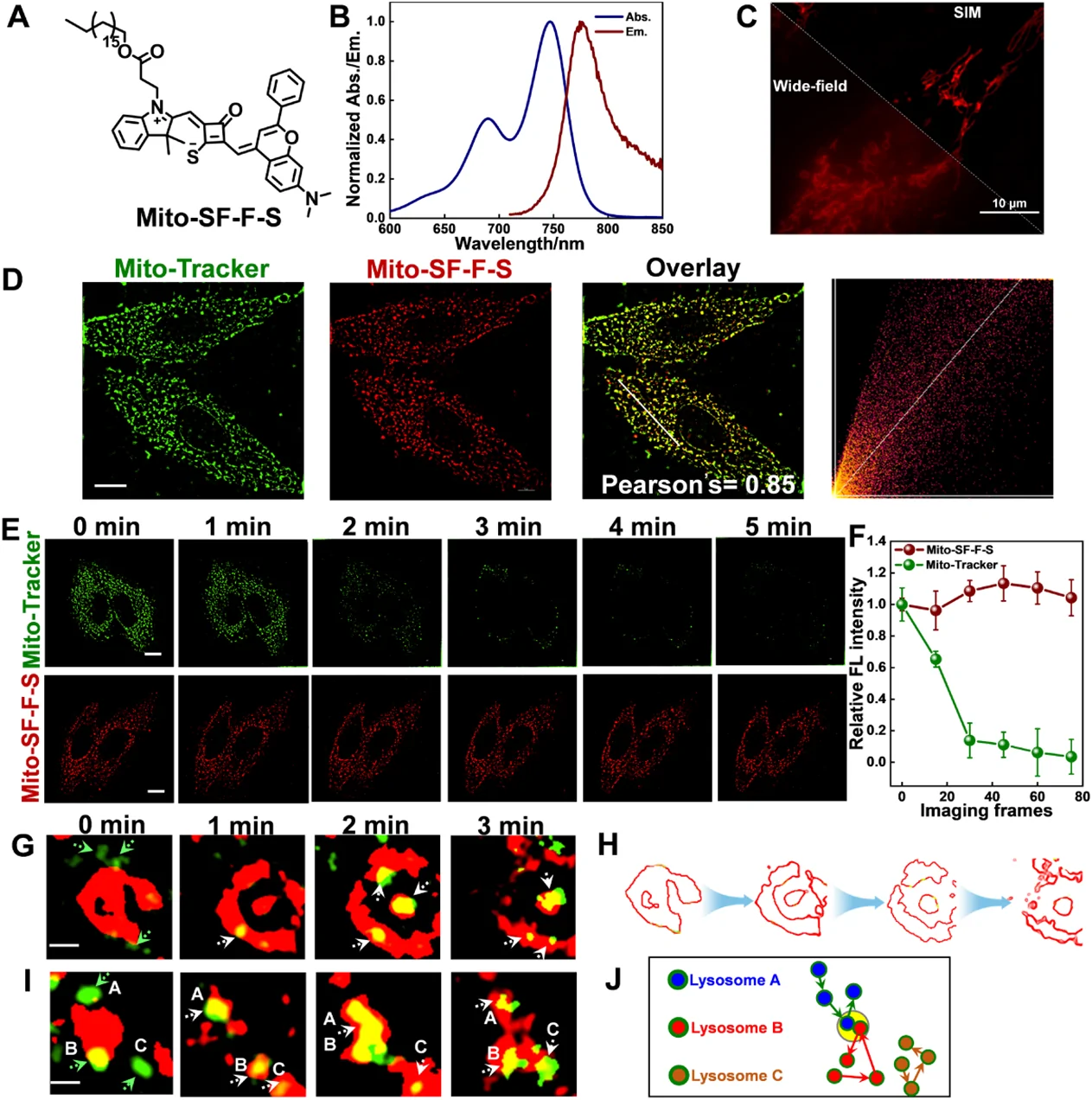
(A) Molecular structure of Mito-SF–F-S. (B) UV–vis-NIR absorption spectrum and fluorescence emission spectrum of Mito-SF–F-S. (C) Comparison between SIM and wide-field images of live cells incubated with Mito-SF–F-S (10 μM). Scale bar: 10 μm. (D) SIM images of live cells incubated with mitochondrial tracker (5 μM) and Mito-SF–F-S (10 μM), and the overlay of the two fluorescence images. Scale bar: 10 μm. (E) SIM images of live cells incubated with mitochondrial tracker (5 μM) and Mito-SF–F-S (10 μM) at different time points (50 W/cm^2^). Scale bars: 10 μm. (F) Change of relative fluorescence intensity of the mitochondrial tracker and Mito-SF–F-S shown in (E). Error bars were calculated from three repeated measurements. (G, I) SIM images of live cells incubated with lysosomal tracker (5 μM) and Mito-SF–F-S (10 μM) and then stimulated with CCCP (10 μM) at different time points. Scale bars: 0.5 μm. (H) Shape changes of mitochondria shown in (G). (J) Trajectories of lysosomes in cells shown in (I). Mito-SF–F-S: λ_ex_ = 640 nm, λ_em_ = 663–738 nm; Mitochondrial tracker: λ_ex_ = 488 nm, λ_em_ = 500–545 nm.

To make full use of SF–F-S, an octadecyl chain was covalently linked to the molecule via an esterification reaction (Mito-SF–F-S) to improve targeting ability (Figure S23).^40^ The absorption and fluorescence-emission spectrum of Mito-SF–F-S were determined and found to be identical with free SF–F-S (Figure 6B), exhibiting superior photostability in vitro (Figure S24), illustrating that this fluorescent tracker exhibited the same advantages, including photostability, low phototoxicity, and high brightness. To highlight the advantages of SIM in imaging, a comparison between SIM and wide-field imaging was performed. As shown in Figure 6C and Figure S25, the SIM imaging sharply captures the morphology and clearly delineates the mitochondrial morphology, whereas the corresponding structures appear blurred and poorly defined in the wide-field image. A magnified view of the region of interest (ROI) explicitly demonstrates that adjacent mitochondria are clearly differentiated in the SIM image, while they appear overlapping and unresolvable in the wide-field image (Figure S26 A, C). Furthermore, cross-sectional intensity profile analysis shows a full width at half-maximum (fwhm) of 182 nm for SIM, significantly narrower than that obtained in the wide-field image (395 nm, Figure S26 D). These quantitative results demonstrate the substantially improved spatial resolution achieved by SF–F-S under SIM imaging conditions. When cells were coincubated with Mito-SF–F-S and a commercial mitochondrial tracker, the green and red fluorescence signals overlapped well, and the Pearson’s colocalization coefficient was calculated to be 0.85 (Figure 6D and Figure S27), confirming the mitochondria-targeting ability of Mito-SF–F-S. Then we evaluated the photobleaching resistance of Mito-SF–F-S and the mitochondrial tracker under the same conditions. Under light illumination, the green fluorescence generated from cells incubated with the mitochondrial tracker gradually reduced with time (Figure 6E), and the fluorescence intensity decreased to 13.8% after the acquisition of 30 images (at the time point corresponding to 2 min) (Figure 6F). But the signal of Mito-SF–F-S remained stable during this process, reflecting the favorable photostability of Mito-SF–F-S, making it suitable for persistent fluorescence imaging.

### Prolonged Imaging of the Deformation of Mitochondria During Mitophagy

Having confirmed the long-term imaging ability of Mito-SF–F-S in live cells, we sought to monitor the deformation of mitochondria during organelle interactions by using SIM. Cells were incubated with Mito-SF–F-S and a lysosome tracker, and then stimulated using carbonyl cyanide 3-chlorophenylhydrazone (CCCP) to induce mitophagy.^41^ Two sites in the cells were selected to investigate the mitochondria and lysosomes (Figure S28). As shown in Figure 6G, red mitochondria exhibited typical filamentous and network-like morphology, and the green lysosomes exhibited speck-like morphology. Significantly, the overlapped areas (yellow regions) of the mitochondria and lysosomes were minimal before stimulation, indicating the normal state of the organelles (0 min). However, the mitochondria began cracking after mitophagy, and the yellow region gradually expanded (1 and 2 min). The integral mitochondria were broken into numerous fragments at 3 min, revealing the breakage of mitochondria during mitophagy and the reduced function of lysosomes.^42,43^ Meanwhile, during the period of mitophagy (0–3 min), the signal of the lysosome tracker remained stable throughout (Figure S29), further demonstrating that the changes of the yellow fluorescence signal can be attributed to organelle interactions. As such, Mito-SF–F-S can monitor structural changes of the mitochondria, Figure 6G, clearly highlighting the process of mitochondrial fragmentation (Figure 6H). At another location, three different lysosomes can be observed, with each exhibiting heterogeneous motility and positional shifts as the experiment progressed (green fluorescence, Figure 6I). By constructing a sequence of temporal snapshots, we generated a comprehensive trajectory map that illustrates the spatiotemporal dynamics of the lysosomes (Figure 6J). It can be observed that lysosomes A and B converge at the mitochondria at 2 min, followed by a subsequent dissociation. These trajectory analyses were reconstructed from discrete time-point imaging, which represents an inherent limitation in the quantitative characterization of lysosomal dynamics, and future studies will focus on continuous time-lapse imaging for revealing the dynamic behavior of lysosomes during mitophagy. Altogether, these imaging results confirm that Mito-SF–F-S can be used to visualize dynamic changes in mitochondria.

In order to visualize the deformation of mitochondria in detail, live cells incubated with Mito-SF–F-S were treated with CCCP, and the changes of fluorescence signals were monitored (Figure S30). From the enlarged images (Figure 7A), the mitochondria maintained structural integrity during the period (0–14 min). However, after being stimulated with CCCP, one of the mitochondria in the live cell divided into three parts (Figure 7B, 2–6 min), and this trend continued as time increased. We can clearly observe that the three sections of the mitochondria gradually moved away (8 and 10 min), and the parts were clearly separated at 12 min. Ultimately, a number of fragments can be found for the mitochondria (14 min),^44^ exhibiting distinct differences from normal cells. We quantified the mean length and area of mitochondria to further monitor the changes. The mean length of the mitochondria in live cells maintained the normal state throughout, but the length of mitochondria increased at the beginning of mitophagy and then distinctly decreased (Figure 7C). While the area of fragments was calculated to be 49.8% compared with the initial mitochondria (Figure 7D). These quantitative results demonstrate that the mitochondria experienced significant shape changes during mitophagy. To further validate these findings, the cells were incubated with 5,5′,6,6’-tetrachloro-1,1’,3,3′-tetraethylbenzimidazolylcarbocyanine iodide (JC-1, an indicator of mitochondrial membrane potential) under the same conditions.^45^ The intensity of red fluorescence was much larger than that of green fluorescence in normal live cells (Figure S31), indicating the aggregation of JC-1 in healthy mitochondria. But the reduced red fluorescence and enhanced green fluorescence indicated that the mitochondrial membrane potential was significantly depolarized (Figure S32), indicating the presence of mitochondrial dysfunction induced by CCCP. All these results confirmed that the persistent imaging ability of Mito-SF–F-S enables the real-time imaging of mitochondrial deformation in live cells.

**Figure 7:**
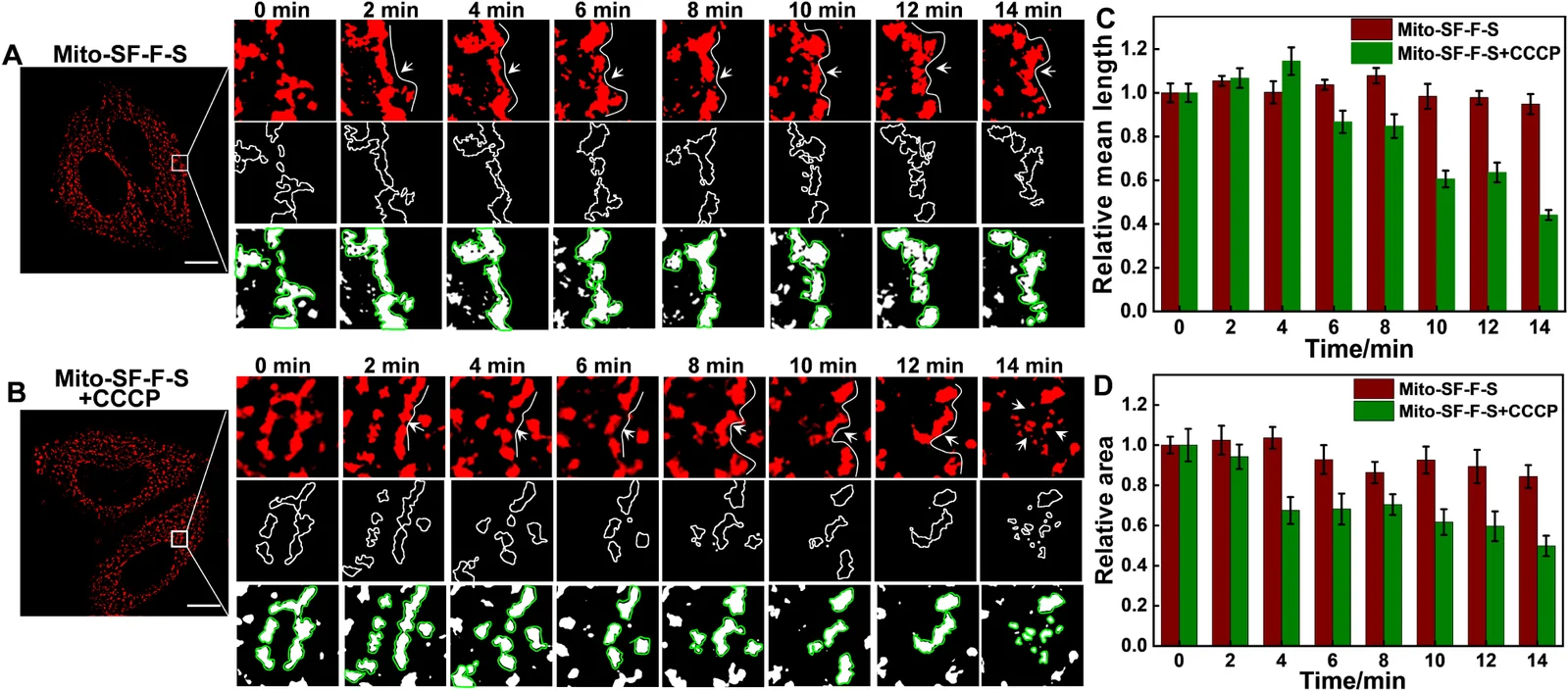
SIM images of live cells incubated Mito-SF–F-S (15 μM, λ_ex_ = 640 nm, λ_em_ = 663–738 nm) in the (A) absence and (B) presence of CCCP (10 μM) at different time points (0, 2, 4, 6, 8, 10, 12, and 14 min) (50 W/cm^2^). Scale bars: 10 μm. The ROI area: 5 × 5 μm. (C) The relative mean length and (D) area of the mitochondrial perimeter under different conditions shown in (A) and (B). Error bars were calculated from three repeated measurements.

## Conclusions

In conclusion, we developed an approach to enhance the photostability of squaraine fluorophores using an asymmetric sulfhydryl-substituted molecular structure. Compared with conventional squaraine fluorophores, the asymmetric structure and sulfhydryl substitution synergistically boost the energy reversibility to prolong the fluorescence lifetime of squaraine. Theoretical calculations, in vitro optical experiments, and cell imaging confirm that the fluorophore exhibits excellent photostability and low phototoxicity. Using this dye as a fluorescent tag, we synthesized a mitochondria-targeting tracker to monitor, in real time, organelle interactions during mitophagy. Resulting from the excellent photophysical and chemical properties of the as-developed dye, the decomposition of mitochondria under stimulation could be imaged using super-resolution SIM imaging. We anticipate that our strategy can be used to develop additional fluorophores, expanding the range of applications and emphasizing the advantages of asymmetric sulfhydryl-substituted squaraine fluorophores in the field of SIMs.

## Supplementary Material



## Notes

Reagents and instruments, synthesis, fluorescence quantum yield, theoretical calculations, spectrometry, cytotoxicity assay, cell culture, supporting data (PDF)
