# Supplementary material for: Asymmetric Sulfhydryl-Substituted Squaraine Fluorophores for Sustained SIM Imaging in Live Cells
Source: J Am Chem Soc. 2026 Aug 27;148(35):37751–61. doi: 10.1021/jacs.6c06633 (PMC13564421; doi:10.1021/jacs.6c06633)
Supplement: Supplementary file 1 [file ja6c06633_si_001.pdf]

Supporting information for

Asymmetric Sulfydryl-Substituted Squaraine Fluorophores for Sustained SIM Imaging in Live Cells

Yibo Zhou,[1,2] Pin Shang,[1] Huiqiu Shi,[1] Jun Zhang,[1] Fan Li,[1] Zhihe Qing*,[1] and Tony D. James*,[2,3]

[1]Hunan Provincial Key Laboratory of Cytochemistry, School of Chemistry and Pharmaceutical Engineering, Changsha University of Science and Technology, Changsha, 410114, P. R. China.

[2]Department of Chemistry, University of Bath, Bath BA2 7AY, U.K.

[3]School of Chemistry and Chemical Engineering, Henan Normal University, Xinxiang 453007, China.

1. Experimental reagents and instruments

The chemicals used in this work for synthesizing the squaraine derivatives were purchased from commercial suppliers, and the water was purified using a Milli-Q water system (Millipore, USA) in our laboratory. The organic solvents, including tetrahydrofuran (THF), dichloromethane (DCM), dimethylformamide (DMF) and acetonitrile (ACN) were dried by refluxing for 24 hours over calcium hydride. The live cells were purchased from Central South University. The lysosomal tracker (Lyso-Tracker Green), mitochondrial tracker (Mito-Tracker Green), Hoechst 33342, 2',7'-Dichlorodihydrofluorescein diacetate (DCFH-DA), 5,5’,6,6’-tetrachloro-1,1’,3,3’-tetraethylbenzi midazolylcarbocyanine iodide (JC-1) and 9,10-anthracenediyl-bis(methylene)-dimalonic acid (ABDA) were purchased from Changsha Well Biological Science Company (China).

^{1}H and ^{13}C NMR spectra were recorded using a Bruker AVANCE NEO 400 NMR spectrometer, and the mass spectra were measured using an ultra-high resolution time-of-flight liquid chromatography-mass spectrometry (Shimadzu LCMS 9030, Japan). Density functional theory (DFT) calculations were performed by Changsha Well Biological Science Company (China). Ultraviolet-Visible spectrophotometer (UV-2700, Shimadzu, Japan) and Photon Technology International (Horiba PTI Quanta Master 400) were used to record the absorption and fluorescence emission spectra. Laser Diode Controller (TLD, Changchun Laser Technology CO., Ltd. ADR-1805) was used as light source in the photostability assays. MTT assays were measured using a Benchmark Plus (Bio-Rad Instruments Inc., Japan). Structured illumination microscopy (SIM) images were obtained on Nikon (N-SIM S) super-resolution microscopes. The large-scale instruments were available in the Analyses and Testing Center of School of Chemistry and Pharmaceutical Engineering in Changsha University of Science and Technology.

2. Synthesis

Synthesis of control squaraine fluorophore (SF). The synthesis of SF was according to the reported literature.[1]

Synthesis of flavylium-substituted squaraine fluorophore (SF-F). The synthesis of SF-F is given in **Scheme S1A**.

Synthesis of compound 1. The synthesis of compound 1 was according to the reported literature.[2] ^{1}H NMR (400 MHz, Methanol-d_4) δ 6.99 (t, J = 8.1 Hz, 2H), 6.29 (d, J = 2.5 Hz, 1H), 6.27 (d, J = 2.5 Hz, 1H), 6.22 (t, J = 2.3 Hz, 2H), 6.17 (d, J = 2.4 Hz, 1H), 6.15 (d, J = 2.4 Hz, 1H), 2.87 (s, 6H), 2.04 (t, J = 6.1 Hz, 3H). ^{13}C NMR (101 MHz, Methanol-d_4) δ 159.09, 153.73, 140.15, 130.86, 130.65, 125.50, 114.69, 106.17, 105.40, 101.36, 41.10, 30.77. MS (ESI) m/z calcd for $C_{18}H_{18}NO^+$ 264.35, found 264.33.

Synthesis of compound 2. 2,3,3-trimethyl-3H-indole-N-propionic acid (586 mg, 1.41 mmol) and 3,4-diisopropoxycyclobutane-1,2-dione (158 mg，0.823 mmol) were dissolved in ethyl alcohol (EtOH, 7 mL) containing trimethylamine (200 μL), the mixture was stirred at 70 °C for 3 h under an argon atmosphere. After the reaction was complete, the solution was extracted with ethyl acetate (EtOAc), and the organic phase was dried using anhydrous sodium sulfate before undergoing vacuum distillation to remove the organic solvent. The crude product was purified via chromatography (MeOH/CH_2Cl_2=1:10) to yield a yellow liquid (100 mg, 30.6% yield). ^{1}H NMR (400 MHz, DMSO-d_6) δ 7.44 – 7.34 (m, 1H), 7.29 – 7.21 (m, 1H), 7.15 (d, J = 7.9 Hz, 1H), 7.03 (t, J = 7.4 Hz, 1H), 5.37 (s, 1H), 4.12 (t, J = 7.2 Hz, 2H), 2.59 (s, 1H), 2.47 (p, J = 1.8 Hz, 1H), 1.51 (s, 6H), 1.42 (d, J = 6.2 Hz, 6H). ^{13}C NMR (101 MHz, $CDCl_3$) δ 192.93, 187.41, 175.26, 173.62, 167.94, 141.92, 140.73, 127.94, 123.03, 122.07, 108.49, 81.62, 78.55, 70.25, 47.96, 38.34, 27.02, 23.09. MS (ESI) m/z calcd for $C_{21}H_{23}NO_5$ 369.42, found 370.17.

Synthesis of compound 3. Compound 2 (100 mg, 0.252 mmol) and HCl (4N, 4 mL) were added into a round-bottom flask containing EtOH (5 mL), the mixture was heated to 70 °C and reacted for 0.5 h. Then the solution was cooled to room temperature and extracted with EtOAc, the obtained organic phase was dried by anhydrous sodium sulfate and the product was acquired by vacuum distillation to remove the organic solvent (50 mg, 60%). ^{1}H NMR (400 MHz, DMSO-d_6) δ 7.26 (s, 1H), 7.17 (s, 1H), 6.90 (s, 2H), 5.49 (s, 1H), 4.04 (d, J = 28.2 Hz, 2H), 2.62 (d, J = 43.5 Hz, 2H), 1.61 (s, 6H). ^{13}C NMR (101 MHz, DMSO) δ 171.43, 143.74, 127.67, 127.67, 122.03, 120.40, 107.44, 60.72, 46.31, 31.16, 27.96. MS (ESI) m/z calcd for $C_{18}H_{17}NO_5$ 327.34, found 345.15 (+NH_4^+).

Synthesis of SF-F. Compound 3 (52 mg, 0.159 mmol) and compound 1 (70 mg，0.151 mmol) were dissolved in the butanol (2 mL), then the benzene (2 mL) and quinoline (140 μL)

were also added to react at 85 °C for 13 h in the dark environment. After the reaction was complete, the solvent was removed by vacuum distillation and the crude product was purified via chromatography (MeOH/CH_2Cl_2=1:20) to yield SF-F as a green liquid (15 mg, 22.6% yield). ^{1}H NMR (400 MHz, $CDCl_3$) δ 9.34 (d, *J* = 1.8 Hz, 1H), 8.22 – 8.10 (m, 2H), 7.87 (td, *J* = 5.9, 3.0 Hz, 1H), 7.56 – 7.43 (m, 3H), 7.39 – 7.24 (m, 3H), 7.15 (t, *J* = 7.4 Hz, 1H), 7.08 (d, *J* = 7.9 Hz, 1H), 6.74 (dd, *J* = 6.2, 3.1 Hz, 1H), 6.54 – 6.45 (m, 1H), 5.96 (s, 1H), 4.11 (q, *J* = 7.2 Hz, 2H), 3.11 (d, *J* = 2.7 Hz, 6H), 2.83 (t, *J* = 7.2 Hz, 2H), 1.79 (s, 6H). ^{13}C NMR (101 MHz, $CDCl_3$) δ 183.45, 170.79, 155.80, 155.43, 153.49, 145.67, 142.05, 132.19, 130.60, 128.96, 127.85, 125.91, 123.82, 122.34, 111.91, 111.60, 97.61, 61.21, 49.29, 40.16, 29.34, 27.14, 14.07. MS (ESI) m/z calcd for $C_{36}H_{32}N_2O_5$ 572.66, found 572.61.

Synthesis of sulfydryl and flavylium-substituted squaraine fluorophore (SF-F-S). The synthesis of SF-F-S is given in **Scheme S1B.** SF-F (50 mg, 0.087 mmol) was added into a round-bottom flask containing toluene (3 mL), and Lawesson's reagent (34 mg, 0.084 mmol) was also added to react at room temperature for 5 h in a dark environment. After the reaction was complete, the solvent was removed by vacuum distillation and the crude product was purified via chromatography (MeOH/CH_2Cl_2=40:1) to yield a green solid (20 mg, 39.1% yield). ^{1}H NMR (400 MHz, $CDCl_3$) δ 9.19 (s, 1H), 8.15 (d, *J* = 7.5 Hz, 2H), 8.07 – 7.98 (m, 1H), 7.53 (dq, *J* = 14.1, 7.2 Hz, 4H), 7.41 – 7.29 (m, 2H), 7.23 – 7.10 (m, 2H), 6.94 (s, 1H), 6.75 (dd, *J* = 9.3, 2.8 Hz, 1H), 6.53 (d, *J* = 2.9 Hz, 1H), 6.34 (s, 1H), 4.10 (q, *J* = 7.1 Hz, 2H), 3.13 (d, *J* = 2.2 Hz, 6H), 2.90 (t, *J* = 6.9 Hz, 2H), 1.80 (s, 6H). ^{13}C NMR (101 MHz, $CDCl_3$) δ 170.82, 156.04, 153.82, 132.19, 130.84, 129.91, 129.01, 127.95, 126.15, 124.13, 122.32, 112.05, 109.89, 108.17, 97.54, 89.79, 61.27, 49.40, 40.25, 29.35, 27.12, 13.19. MS (ESI) m/z calcd for $C_{36}H_{32}N_2O_4S$ 588.72, found 588.78.

Synthesis of benzoindolenine-substituted squaraine fluorophore (SF-BI). The synthesis of SF-BI is given in **Scheme S1C.** Compound 3 (52 mg, 0.156 mmol) and 1,1,2-Trimethyl-N-ethyl-benz[e]indole (50 mg, 0.129 mmol) were dissolved in butanol (2 mL), then benzene (2 mL) and quinoline (140 μL) were reacted at 85 °C for 15 h in the dark. After the reaction was complete, the solvent was removed by vacuum distillation and the crude product was purified via chromatography (MeOH/CH_2Cl_2=1:75) to yield a blue liquid (25 mg, 14.8% yield). ^{1}H NMR (400 MHz, $CDCl_3$) δ 8.19 (d, *J* = 8.5 Hz, 1H), 7.96–7.83 (m,

2H), 7.58 (t, J = 7.7 Hz, 1H), 7.43 (t, J = 7.6 Hz, 1H), 7.32 (dd, J = 20.2, 8.5 Hz, 3H), 7.13 (t, J = 7.4 Hz, 1H), 7.06 (d, J = 8.0 Hz, 1H), 6.02 (s, 1H), 5.94 (s, 1H), 4.32 (s, 2H), 4.17–3.97 (m, 2H), 3.73 (s, 3H), 2.83 (t, J=7.3 Hz, 2H), 2.05 (s, 6H), 1.35–1.18 (m, 6H). ^{13}C NMR (101 MHz, $CDCl_3$) δ 131.47, 129.83, 129.79, 128.58, 127.80, 127.45, 124.56, 123.60, 122.60, 122.31, 110.05, 61.17, 51.40, 29.71, 27.22, 14.05. MS (ESI) m/z calcd for $C_{35}H_{34}N_2O_4$ 546.67, found 546.65.

Synthesis of benz[cd]indol-substituted squaraine fluorophore (SF-BcI). The synthesis of SF-BcI is given in **Scheme S1D.**

Synthesis of compound 4. The benzo[cd]indol-2(1H)-one (150 mg, 0.89 mmol) and NaH (56 mg, 0.133 mmol) were dissolved in anhydrous DMF (50.0 mL), and iodoethane (86 mg, 0.106 mmol) was also added to react at 90 °C for 14 h, then the solution was extracted with EtOAc and the obtained organic phase was purified by removing the organic solvent under reduced pressure. The crude product was purified with silica gel column chromatography to afford a yellow oil product (90 mg, 51.1% yield). ^{1}H NMR (400 MHz, $CDCl_3$) δ 7.93 (d, J = 7.0 Hz, 1H), 7.82 (d, J = 8.1 Hz, 1H), 7.57 – 7.51 (m, 1H), 7.35 (s, 1H), 7.34 – 7.27 (m, 1H), 6.73 (d, J = 6.9 Hz, 1H), 3.87 (q, J = 7.2 Hz, 2H), 1.30 (t, J = 7.3 Hz, 3H). ^{13}C NMR (101 MHz, $CDCl_3$) δ 167.48, 138.83, 130.49, 128.33, 126.57, 123.79, 119.97, 104.66, 34.78, 13.99. MS (ESI) m/z calcd for $C_{13}H_{11}NO$ 197.24, found 198.09.

Synthesis of compound 5. The intermediate products (100 mg, 0.5 mmol) were dissolved with anhydrous THF (2.0 mL) in an ice bath, then methylmagnesium chloride (3 M solution in THF, 1.0 mL, 0.61 mmol) was added to react at 65 °C for 2 h. After that, the solution was acidified using HCl solution (1 mL, 2 M) and the KI was added to the solution, and the crude product was filtered to obtain compound 5 (71.6 mg, 73% yield). ^{1}H NMR (400 MHz, D_2O) δ 8.53 (d, J = 7.3 Hz, 1H), 8.46 (d, J = 8.1 Hz, 1H), 8.14 (d, J = 8.3 Hz, 1H), 8.07 (d, J = 7.4 Hz, 1H), 7.89 (s, 1H), 7.73 (t, J = 7.8 Hz, 1H), 4.50 (q, J = 7.4 Hz, 2H), 2.55 (s, 3H), 1.44 (t, J = 7.4 Hz, 3H). ^{13}C NMR (101 MHz, D_2O) δ 138.60, 138.09, 134.28, 130.92, 130.47, 129.24, 120.18, 42.05, 38.55, 14.13. MS (ESI) m/z calcd for $C_{14}H_{14}N^+$ 196.27, found 196.12.

Synthesis of SF-BcI. Compound 5 (80 mg, 0.151 mmol) and compound 3 (60 mg, 0.159 mmol) were dissolved in n-butanol (2 mL), then the benzene (2 mL) and quinoline (140 μL)

were also added to react at 85 °C for 13 h in the dark. After the reaction was complete, the solvent was removed by vacuum distillation and the crude product was purified via chromatography (MeOH/CH_2Cl_2=1:25) to yield a green liquid (8 mg, 5.7% yield). ^{1}H NMR (400 MHz, DMSO-d_6) δ 8.92 (s, 1H), 8.03 (d, J = 8.1 Hz, 1H), 7.79 (t, J = 7.7 Hz, 1H), 7.58 (t, J = 8.4 Hz, 3H), 7.47 – 7.38 (m, 2H), 7.32 (d, J = 5.5 Hz, 1H), 7.25 (d, J = 7.2 Hz, 1H), 6.14 (s, 1H), 5.98 (d, J = 2.2 Hz, 1H), 4.48 (t, J = 6.6 Hz, 2H), 3.99 (q, J = 7.1 Hz, 2H), 2.83 (q, J = 6.8 Hz, 2H), 1.72 (s, 6H), 1.34 (t, J = 7.1 Hz, 3H). ^{13}C NMR (101 MHz, DMSO-d_6) δ 187.29, 180.27, 171.18, 170.76, 142.26, 137.35, 129.17, 128.56, 122.89, 120.57, 111.80, 106.93, 55.35, 49.05, 31.80, 26.46, 14.27, 13.81. MS (ESI) m/z calcd for $C_{32}H_{28}N_2O_4$ 504.59, found 504.77.

Synthesis of sulfydryl-substituted squaraine fluorophore (SF-S). The synthesis of SF-S followed a procedure similar to that of SF-F-S (**Scheme S1E**). SF (50 mg, 0.0925 mmol) was added into a round-bottom flask containing toluene (3 mL), and Lawesson's reagent (35.94 mg, 0.0887 mmol) was also added to react at room temperature for 5 h in a dark environment. After the reaction was complete, the solvent was removed by vacuum distillation and the crude product was purified via chromatography (MeOH/CH_2Cl_2=40:1) to yield a green solid (18 mg, 35.1% yield). ^{1}H NMR (400 MHz, DMSO-d_6) δ 7.52 (d, J = 7.4 Hz, 1H), 7.43 – 7.29 (m, 4H), 7.29 – 7.22 (m, 1H), 7.17 (d, J = 9.9 Hz, 2H), 7.05 – 6.93 (m, 1H), 5.80 (s, 1H), 4.38 (s, 2H), 4.15 – 3.87 (m, 2H), 3.56 (s, 4H), 1.67 (s, 6H), 1.44 (d, J = 9.2 Hz, 6H). ^{13}C NMR (101 MHz, DMSO-d_6) δ 171.29, 130.15, 130.13, 130.11, 128.40, 124.59, 122.73, 52.15, 49.22, 31.76, 31.71, 31.58, 30.85, 30.29, 29.46, 26.96. MS (ESI) m/z calcd for $C_{32}H_{32}N_2O_5S$ 556.68, found 555.07.

Synthesis of Mito-SF-F-S. The synthesis of SF-F is given in **Figure S23.** SF-F-S (28 mg, 0.05 mmol), EDC (13 mg, 0.07 mmol) and DMAP (9 mg, 0.074 mmol) were dissolved with DCM (10 mL) in a round-bottom flask, and the mixture was stirred for 15 min at room temperature. Then 1-Octadecanol (27 mg, 0.1 mmol) and *N,N*-Diisopropylethylamine (19 mg, 0.15 mmol) were added to react for another 5 h, and the reaction was stopped by the addition of saline (50 mL). The solvent was removed at reduced pressure and the crude product was purified via chromatography (MeOH/CH_2Cl_2=1:50) to yield a dark green power (22.7 mg, 54.3% yield). ^{1}H NMR (400 MHz, $CDCl_3$) δ 8.17 (d, J = 7.6 Hz, 2H), 7.99 – 7.83 (m, 1H),

7.62 – 7.41 (m, 3H), 7.38 – 7.28 (m, 2H), 7.23 – 7.03 (m, 2H), 6.84 – 6.62 (m, 2H), 6.55 (s, 1H), 5.94 (s, 1H), 5.35 (t, *J* = 4.9 Hz, 2H), 4.03 (s, 2H), 3.13 (s, 6H), 2.84 (q, *J* = 9.0, 6.9 Hz, 2H), 2.01 (q, *J* = 6.5 Hz, 2H), 1.65 (d, *J* = 8.2 Hz, 6H), 1.54 (t, *J* = 6.7 Hz, 2H), 1.25 (dd, *J* = 8.9, 4.4 Hz, 28H), 0.89 – 0.86 (m, 3H); ^{13}C NMR (101 MHz, $CDCl_3$) δ 155.85, 155.38, 153.51, 132.33, 130.55, 129.91, 128.96, 122.35, 111.87, 97.75, 65.45, 49.39, 31.95, 29.73, 29.69, 28.43, 25.85, 22.72, 14.17.

3. Spectrophotometric experiments.

To investigate the photophysical properties of the squaraine fluorophore, five kinds of squaraine derivatives (SF, SF-F, SF-F-S, SF-BI and SF-BcI, 5 μM) were dissolved in MeOH, EtOH, dimethylsulfoxide (DMSO), acetonitrile (ACN) and PBS, and the UV-absorption spectra and fluorescence emission spectra were recorded with Ultraviolet-Visible spectrophotometer (UV-2700, Shimadzu, Japan) and Photon Technology International (Horiba PTI Quanta Master 400).

4. Fluorescence quantum yield

The fluorescence quantum yield of SF and SF-BI was calculated using cresyl violet as a reference standard ($\Phi_f = 0.54$, MeOH),[3] and the IR780 was used as the reference standard for SF-F, SF-F-S and SF-BcI ($\Phi_f = 0.17$, EtOH).[4] The quantum yield was calculated according to the following formula:

$$\Phi_f = \Phi_r \, (A_r F_f \, / \, A_f F_r) \, (n_f/n_r)^2$$

Where the Φ_r is the quantum yield of reference standard, the A_r and A_f are the absorbance of reference standard and squaraine derivatives, F_r and F_f are the integral area of fluorescence spectra of reference standard and squaraine derivatives, and the n_r and n_f are the refractive index of the solvents.

5. Theoretical calculations

The theoretical calculations of squaraine derivatives were performed by Well Biosciences Co., Ltd in Changsha company. The molecular geometry was fully optimized using density functional theory (DFT) with the B3LYP functional and Def2-SVP basis set. All calculations were performed using the Gaussian 16 software package.

All optimized structures were confirmed to be at local minima on the ground state (S_0),

first singlet excited state (S_1) and triplet excited state (T_1 or T_2) potential energy surfaces, with no imaginary frequencies observed. The HOMO and LUMO energy levels were derived from the optimized structures, and the root-mean-square deviation (RMSD) values were calculated with Multiwfn 3.8 and VMD 1.9.3.

Geometry optimization based on the S_1 state was performed, and spin–orbit coupling (SOC) matrix elements between singlet and triplet excited states were computed at the Def2-SVP level with ORCA 5.0.3. In addition, the vertical ionization potential was evaluated by calculating the energy difference between the neutral and cationic states at the neutral optimized geometry.

6. Assessment of the photostability of squaraine derivatives

Optimizing the power of laser illumination. SF (5 μM), SF-F (5 μM) and SF-F-S (5 μM) were illuminated with a 660 nm laser at different powers (0, 30, 83, 160 and 800 mW) for 1 h, and the absorption spectra were recorded with Ultraviolet-Visible spectrophotometer (UV-2700, Shimadzu, Japan).

Photostability of squaraine derivatives. SF-F (5 μM) and SF-F-S (5 μM) were illuminated with a 660 nm laser (83 mW) for different times (0, 5, 15, 30, 50, 70, 90, 110, 130, 150, 170, 190 and 210 min), and the absorption and fluorescence emission spectra were recorded at different time points.

7. Assessing singlet oxygen generated by squaraine derivatives

To confirm the singlet oxygen generated by squaraine derivatives, 9,10-anthracenediyl-bis(methylene)-dimalonic acid (ABDA) was used as an indicator. Briefly, three kinds of squaraine derivatives (SF, SF-F, SF-F-S, 5 μM) were added into a solution containing ABDA (15 μM), and the absorption spectra were recorded after being illuminated with a 660 nm laser (83 mW) for different times (0, 5, 10, 15, 20, 25, 30, 35 and 40 min). As a control, the ABDA (15 μM) solution without squaraine derivatives was also illuminated with a 660 nm laser (83 mW) for different times (0, 5, 10, 15, 20, 25, 30, 35 and 40 min), and the change of absorption spectra were recorded. After that, the absorption value at 380 nm was obtained to calculate the change of absorption value ratio.

8. Fluorescent lifetime of squaraine derivatives

The fluorescent lifetime of squaraine derivatives was determined by Changsha Well Biological Science Company (China). The fluorescence lifetime spectrum of squaraine derivatives was recorded using a steady state and time-resolved photoluminescence spectrometer (FLS1000 Photoluminescence Spectrometer, Edinburgh Instruments, UK) equipped with a frequency-tunable pulsed laser (pulse repetition rate reduced to 20 Hz). In the lifetime imaging experiments, the settings of excitation/emission wavelengths were based on the parameters shown in Figure 2. The data were analyzed using the LAS X software n-Exponential Tail Fit model for decay curve fitting.

9. Cytotoxicity assay

The biocompatibility of the squaraine derivatives was evaluated using a standard MTT assay.[6] Human hepatoma cell line (HepG2 cell) were purchased from Central South University and used in this work, cultured with Dulbecco's Modified Eagle Medium (DMEM, GIBCO) containing fetal calf serum (FBS) and penicillin-streptomycin (1%) under an atmosphere of 5% CO_2 and 95% air at 37 °C. When the cells were grown up to 90% in the culture flask, the cells were transferred to 96-well plate at a concentration of 5×10^4 cells each well. Then different concentrations of SF, SF-F and SF-F-S (0, 5, 10, 15, 20 and 25 μM) were added and cultured for 24 h. After that, the culture medium was removed and washed with PBS three times, MTT solution (0.5 mg/mL) was added into each well and incubated for another 4 h. The MTT was removed and DMSO (150 μL) was added for shaking for 10 min, the absorption value was recorded with Tecan microplate (ELISA) reader. The survival rate was calculated according to the following equation:

$$\text{Cell viability (\%)} = [A_{\text{sample}} - A_{\text{blank}}] / [A_{\text{control}} - A_{\text{blank}}] \times 100$$

10. Fluorescence imaging of live cells

3D fluorescence imaging in live cells. SF (15 μM), SF-F (15 μM) and SF-F-S (15 μM) were added into a confocal dish and incubated with HepG2 cells for 30 min, then the culture medium was removed and washed with PBS three times before imaging. The SIM images were acquired with the excitation wavelength of 561-640 nm.

Colocalization cell imaging of squaraine derivatives. SF (15 μM), SF-F (15 μM) and SF-F-S (15 μM) were added into a confocal dish and incubated with HepG2 cells for 30 min,

then the culture medium was removed and washed with PBS three times. Fresh culture medium containing mitochondrial tracker (Mito-Tracker Green, 5 μM, λ_{ex}=488 nm, λ_{em}=500-545 nm) or lysosomal tracker (Lyso-Tracker Green, 5 μM, λ_{ex} =488 nm, λ_{em} =500-545 nm) was added and incubated for 20 min, the culture medium was removed and washed with PBS for three times before imaging.

Photostability of squaraine derivatives in live cells. Three groups of HepG2 cells were incubated with SF (15 μM), SF-F (15 μM) and SF-F-S (15 μM) for 30 min, then the culture medium was removed and washed with PBS three times. The SIM images were obtained at different time points (0, 10, 20, 30, 40, 50, 60, 70, 80, 90, 100 and 110 min, 50 W/cm^2). For comparative SIM imaging, all acquisition parameters, including excitation wavelength (640 nm), emission wavelength (663–738 nm), laser power (100 mW), laser utilization efficiency (15%), exposure time (50 ms), imaging frame rate (13 fps), effective temporal resolution (1.16 s/frame), were kept identical for SF, SF-F and SF-F-S.

Phototoxicity and ROS-generation of squaraine derivatives in live cells. Live cells were pre-incubated with Hoechst 33342 (15 μM) for 30 min and further incubated with SF (15 μM), SF-F (15 μM) and SF-F-S (15 μM) for another 30 min. After that, the cells were illuminated with SIM for 50 min, then Propidium Iodide (PI, 5 μM) and Dihydroethidium (DHE, 5 μM) were added to incubate for 50 min. Finally, the cells were washed with PBS and imaged by fluorescence confocal microscopy (Olympus, FV3000).

Colocalization cell imaging of Mito-SF-F-S. Live cells were incubated with Mito-SF-F-S (15 μM) for 30 min, then the culture medium was removed and washed with PBS three times. Fresh culture medium containing mitochondrial tracker (Mito-Tracker Green, 5 μM, λ_{ex}=488 nm, λ_{em}=500-545 nm) was added to incubate for another 20 min, the cells were then washed with PBS before imaging.

Imaging of the organelle interactions during mitophagy. Live HepG2 cells were incubated with Mito-SF-F-S (15 μM) for 30 min, and then lysosomal tracker (Lyso-Tracker Green, 5 μM, λ_{ex} =488 nm, λ_{em} =500-545 nm) was added to incubate for 20 min. After that, the CCCP (10 μM) was added and the cells were imaged with SIM imaging system at different time points (0, 1, 2 and 3 min).

Imaging of the change of mitochondria during mitophagy. Live HepG2 cells were

treated with Mito-SF-F-S (15 μM) for 30 min, and then CCCP (10 μM) was added to incubate for 20 min. The cells were placed in a temperature-controlled microchamber containing CO_2 for SIM imaging, and the images were acquired at different time points (0, 2, 4, 6, 8, 10, 12 and 14 min).

Imaging of the change of mitochondrial membrane potential with JC-1. Live HepG2 cells were incubated with JC-1 (10 μM) for 20 min, then the cells were divided into two groups. One group was further treated with CCCP (10 μM) for 20 min, and then the images were acquired at different time points (0, 2, 6, 8 and 14 min). As a control, the other group was treated with nothing and imaged with SIM under the same conditions.

11. Supporting data

Scheme S1. Synthetic routes of asymmetric squaraine derivatives.

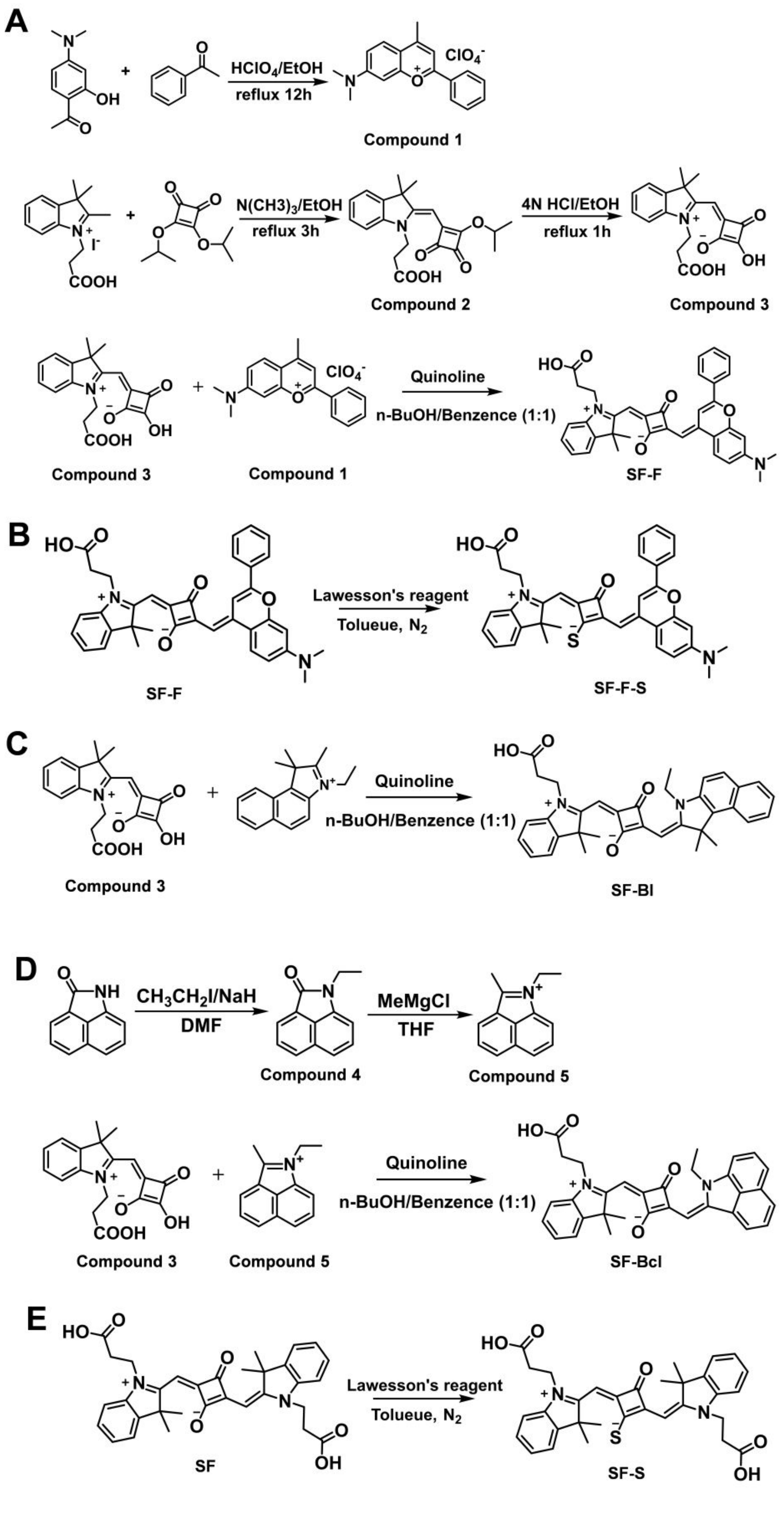

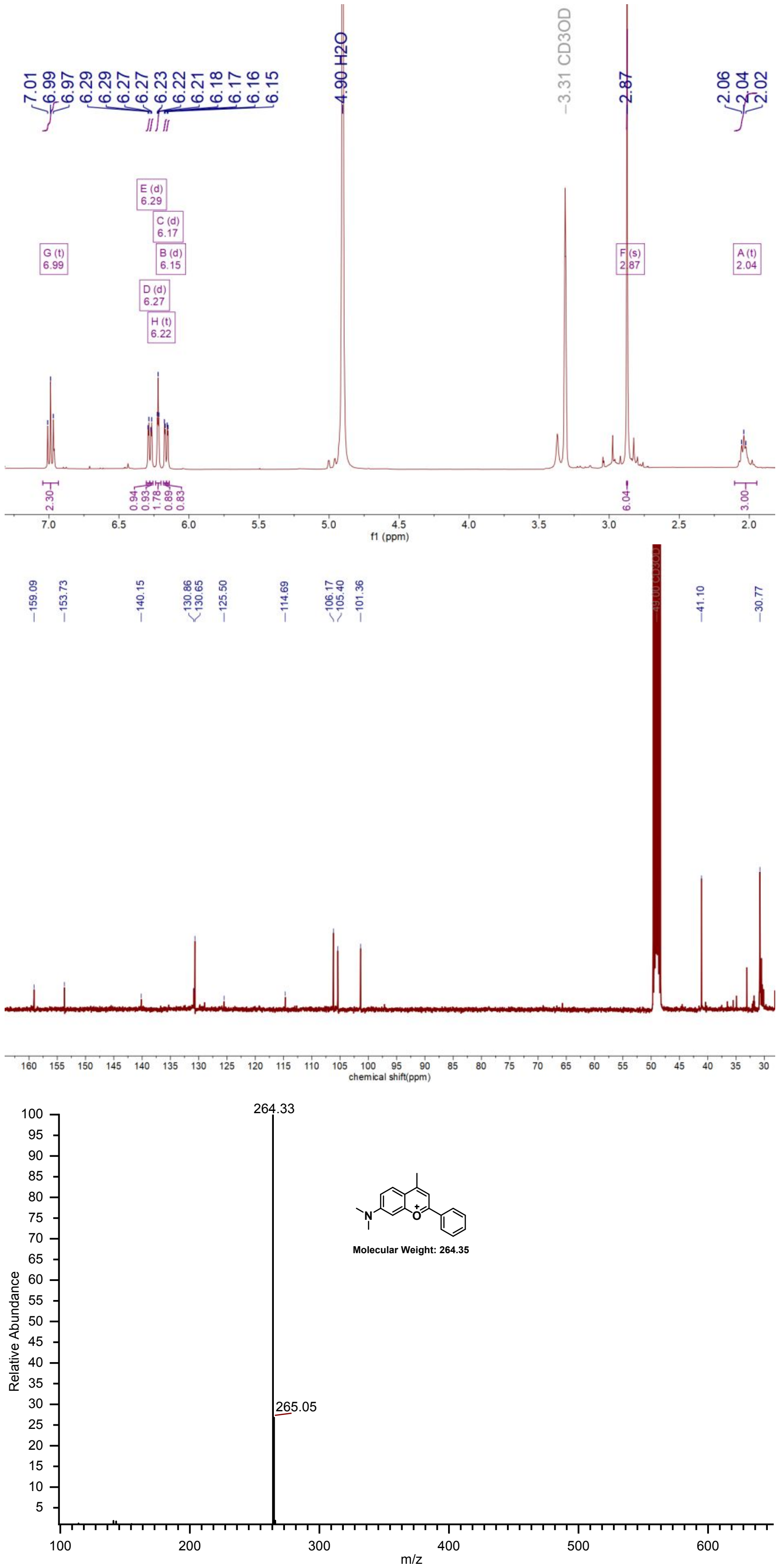

Figure S1. ^{1}H NMR (top), ^{13}C NMR (middle) and ESI-MS (bottom) of compound 1.

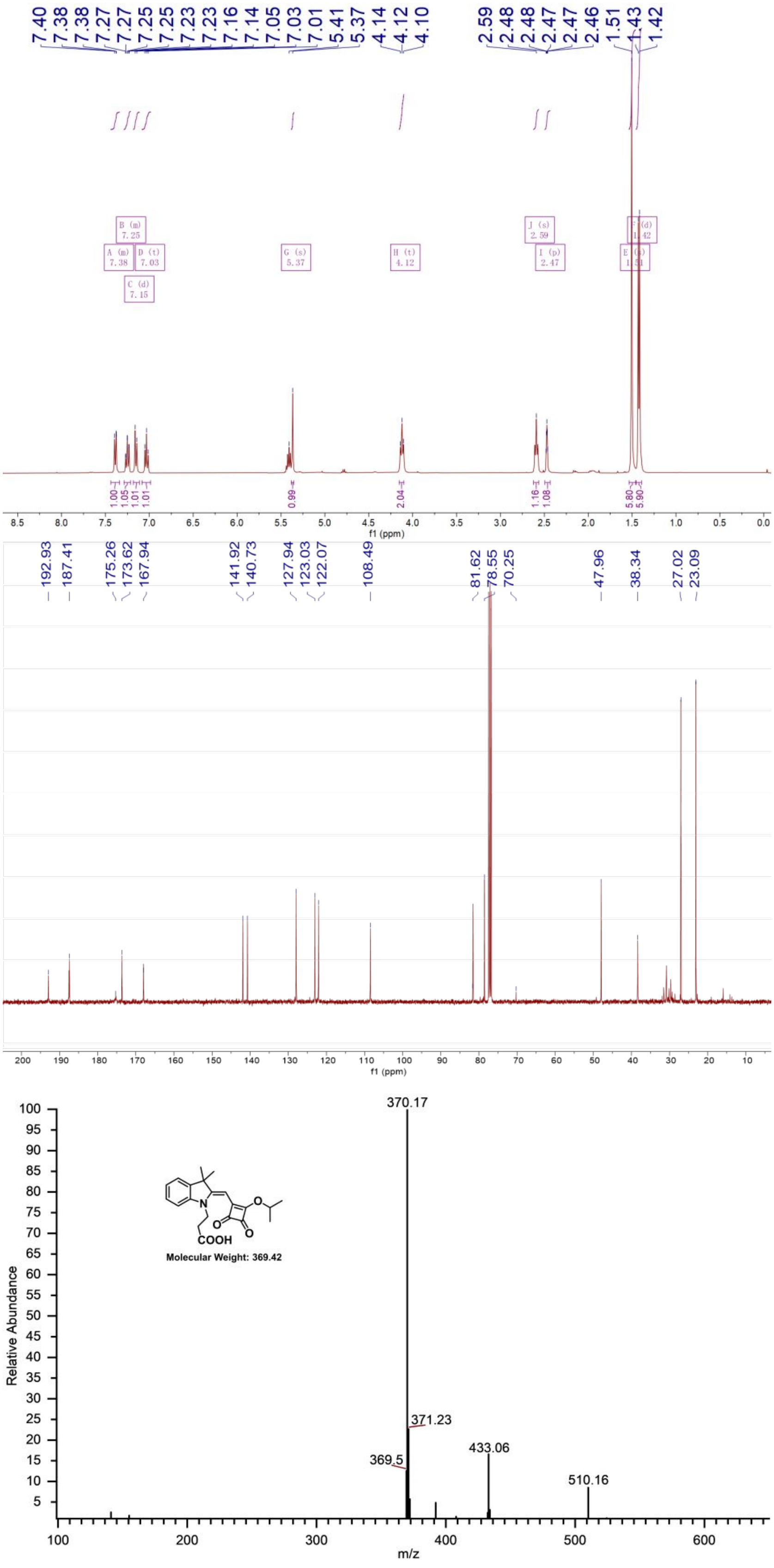

Figure S2. ^{1}H NMR (top), ^{13}C NMR (middle) and ESI-MS (bottom) of compound 2.

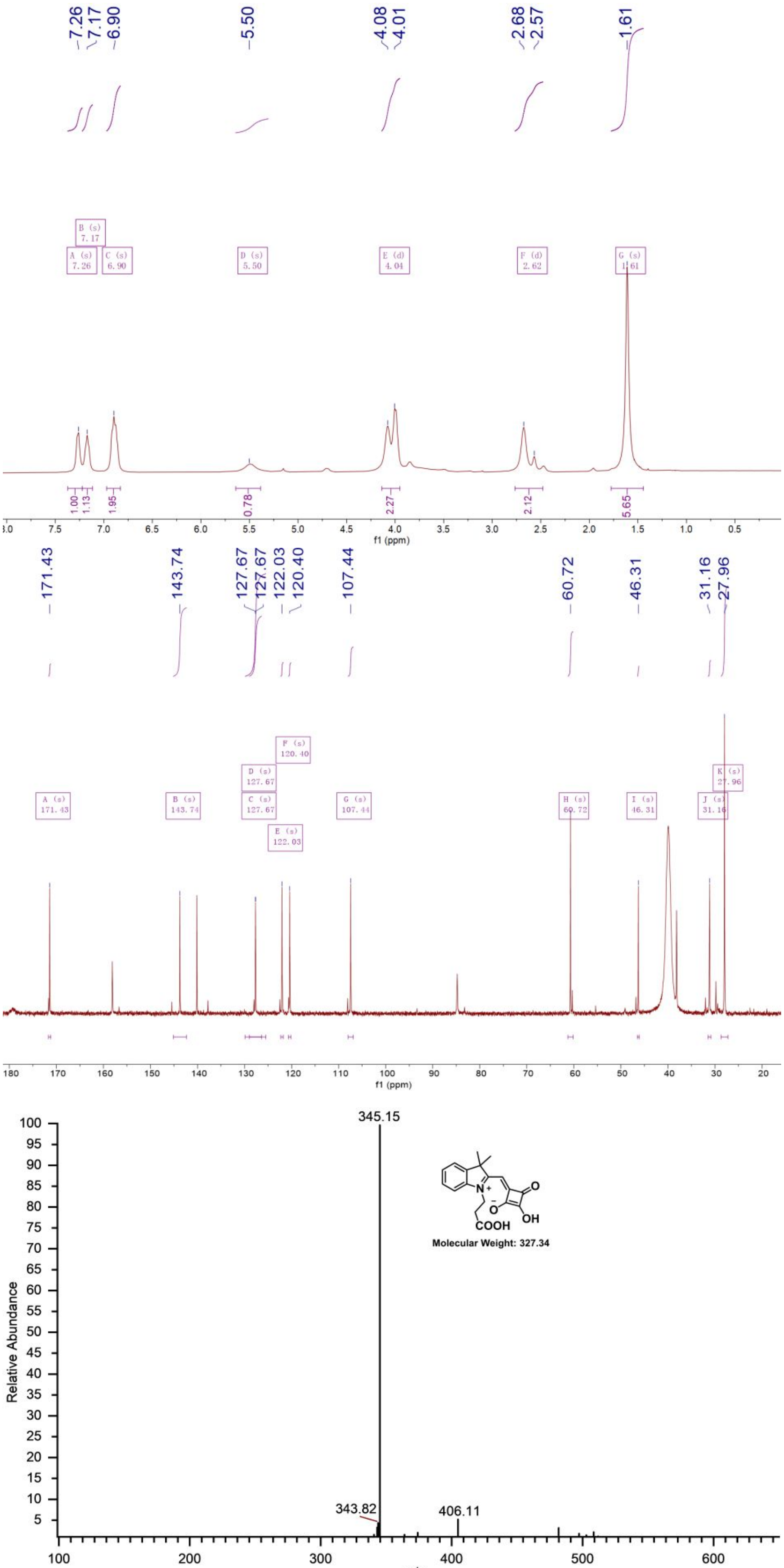

Figure S3. ^{1}H NMR (top), ^{13}C NMR (middle) and ESI-MS (bottom) of compound 3.

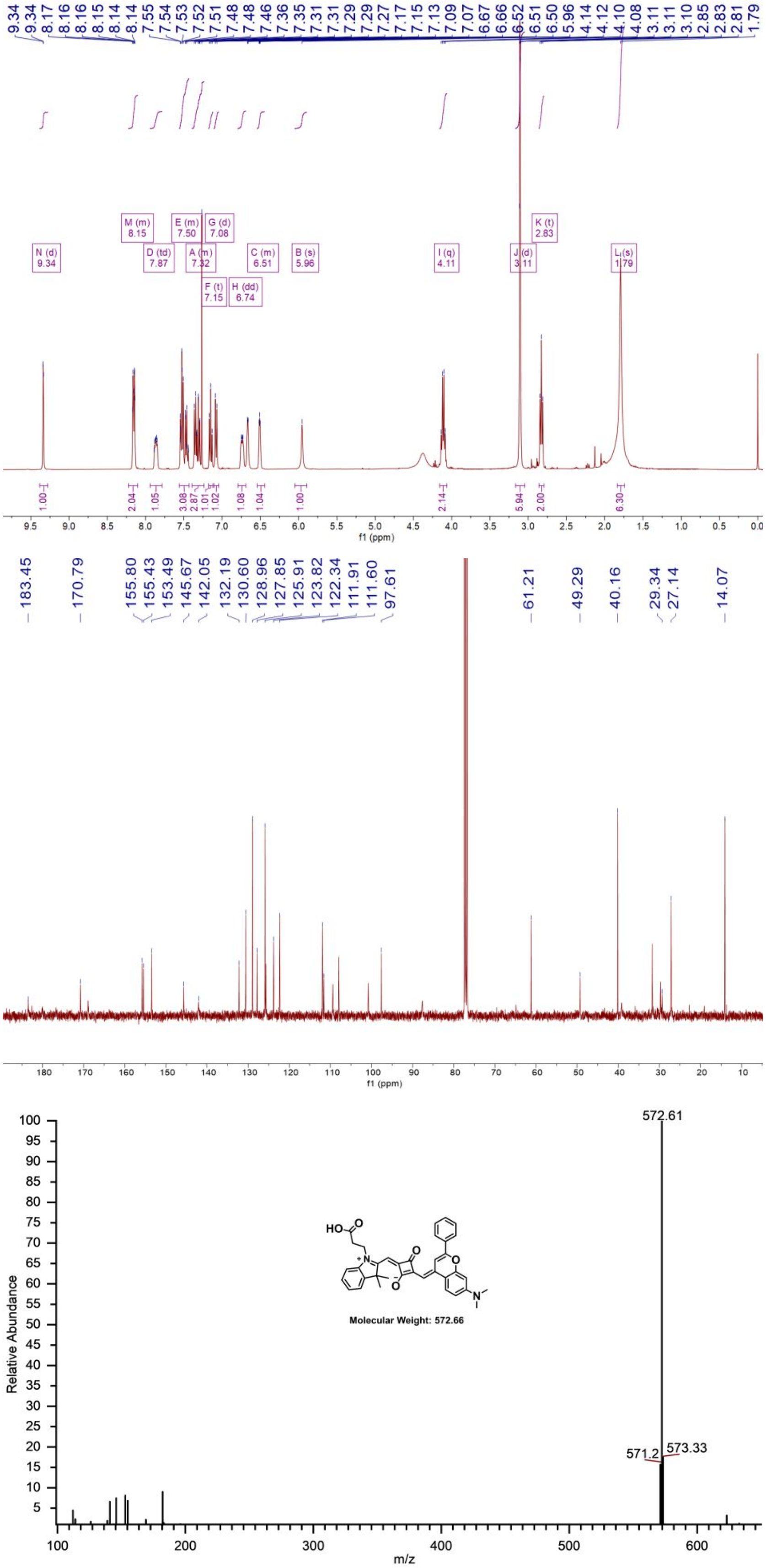

Figure S4. ^{1}H NMR (top), ^{13}C NMR (middle) and ESI-MS (bottom) of SF-F.

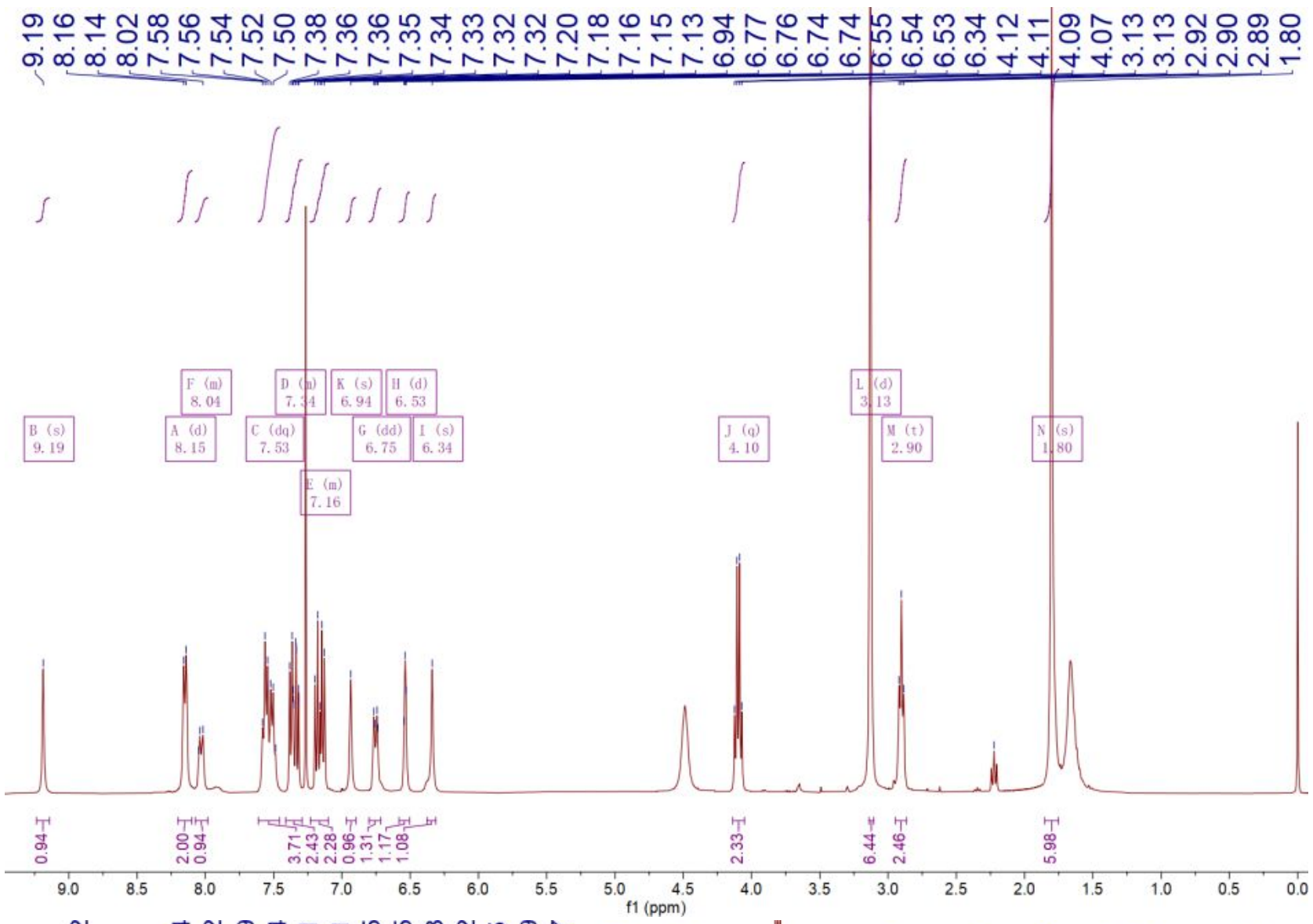

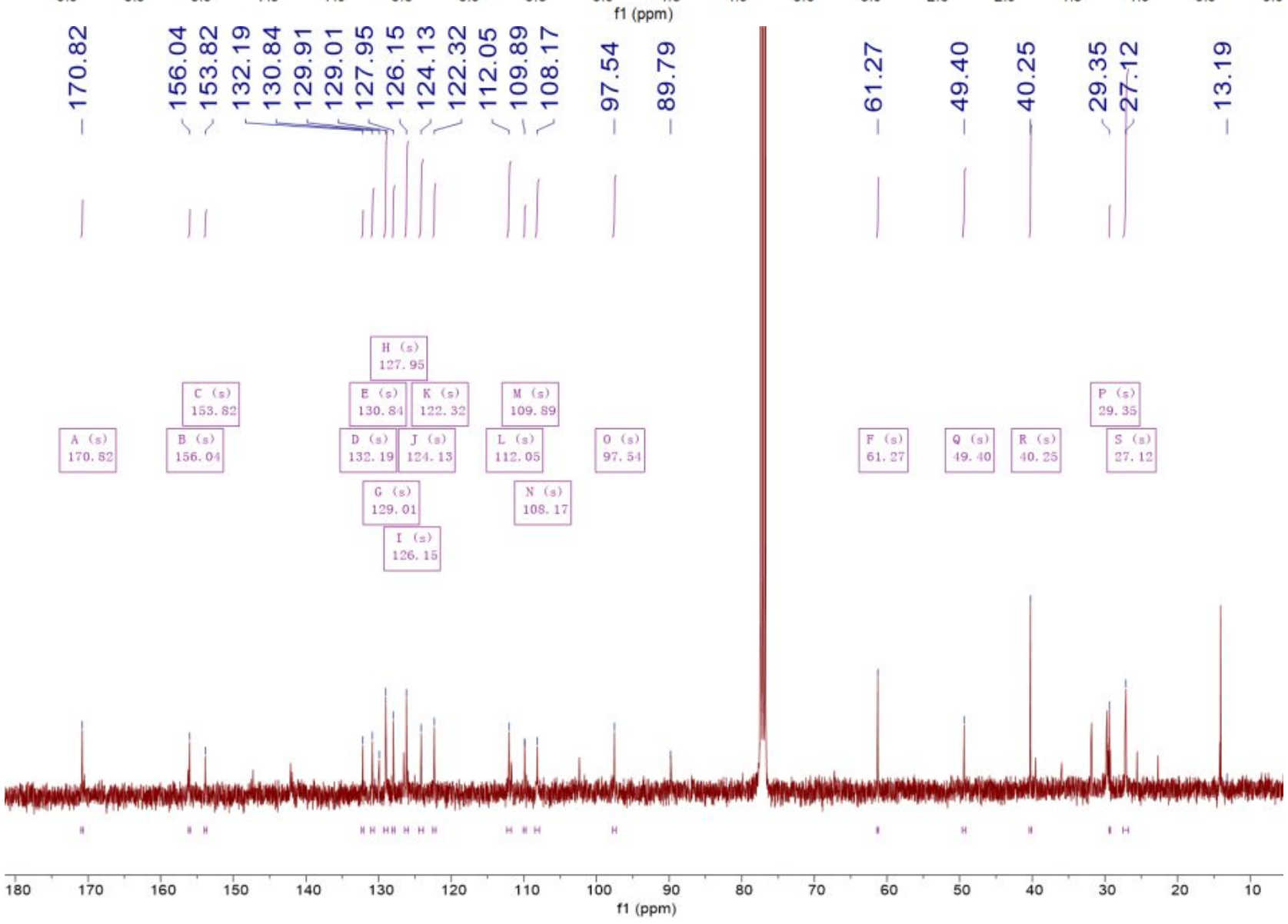

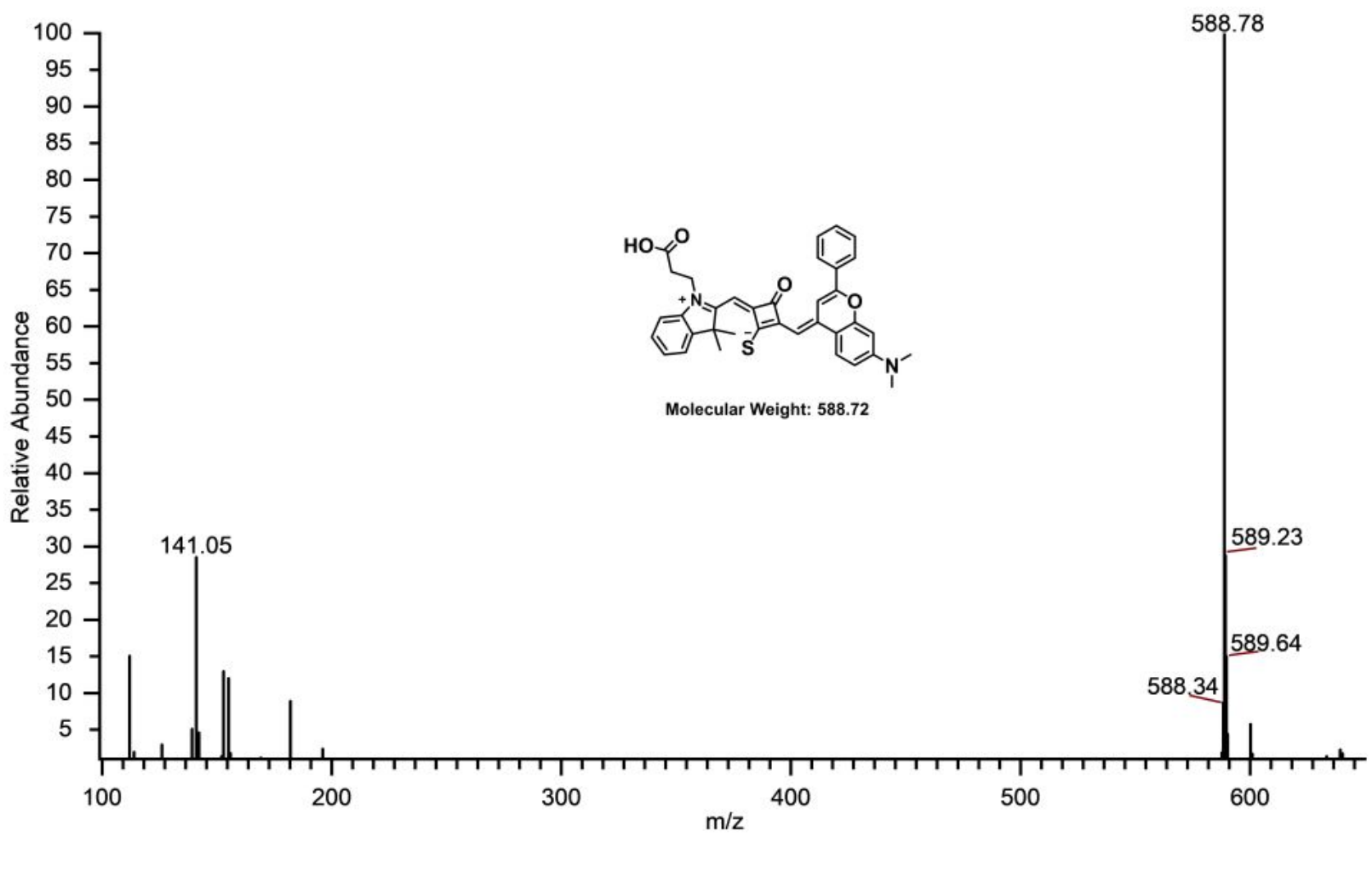

Figure S5. ^{1}H NMR (top), ^{13}C NMR (middle) and ESI-MS (bottom) of SF-F-S.

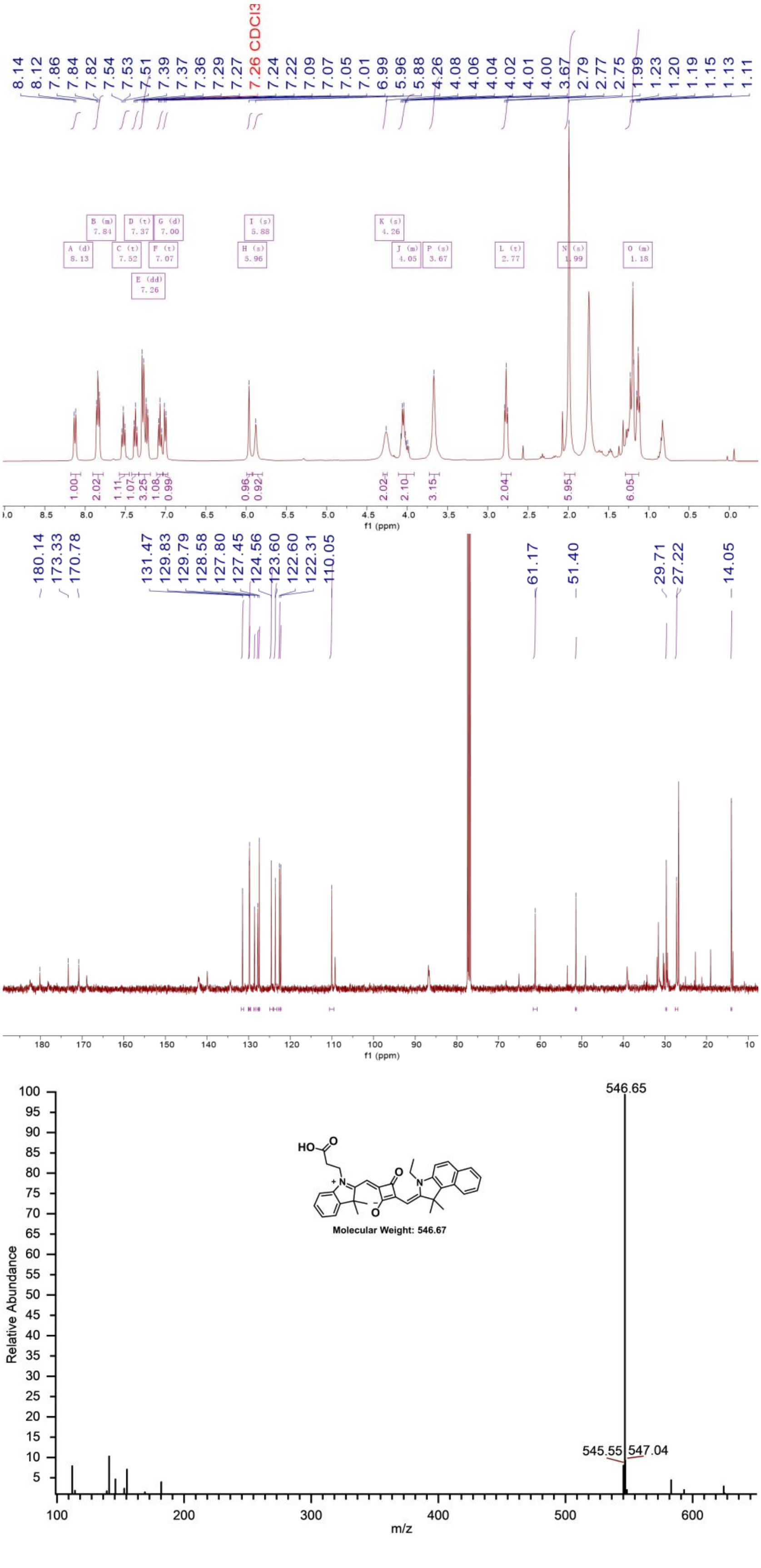

Figure S6. ^{1}H NMR (top), ^{13}C NMR (middle) and ESI-MS (bottom) of SF-BI.

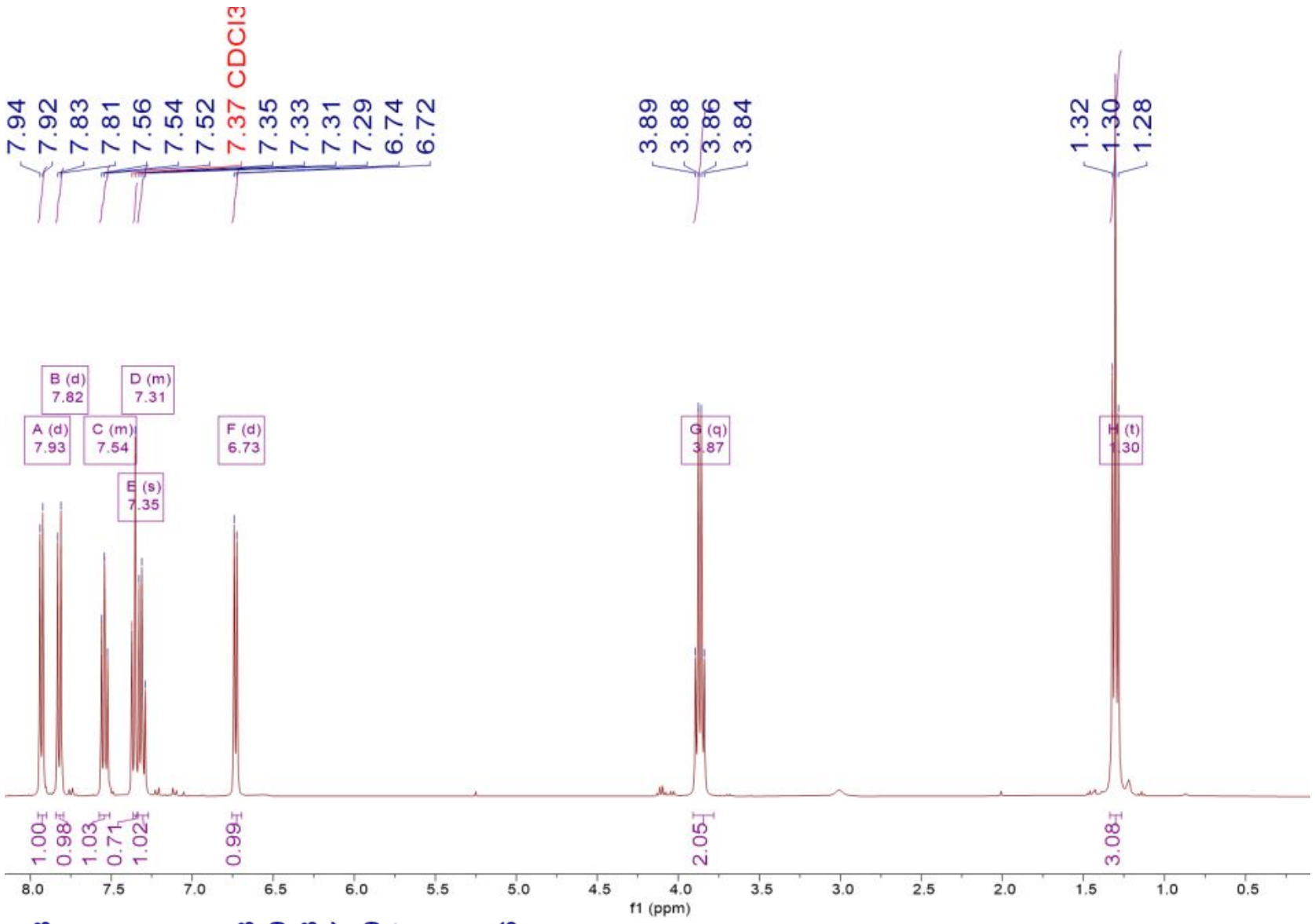

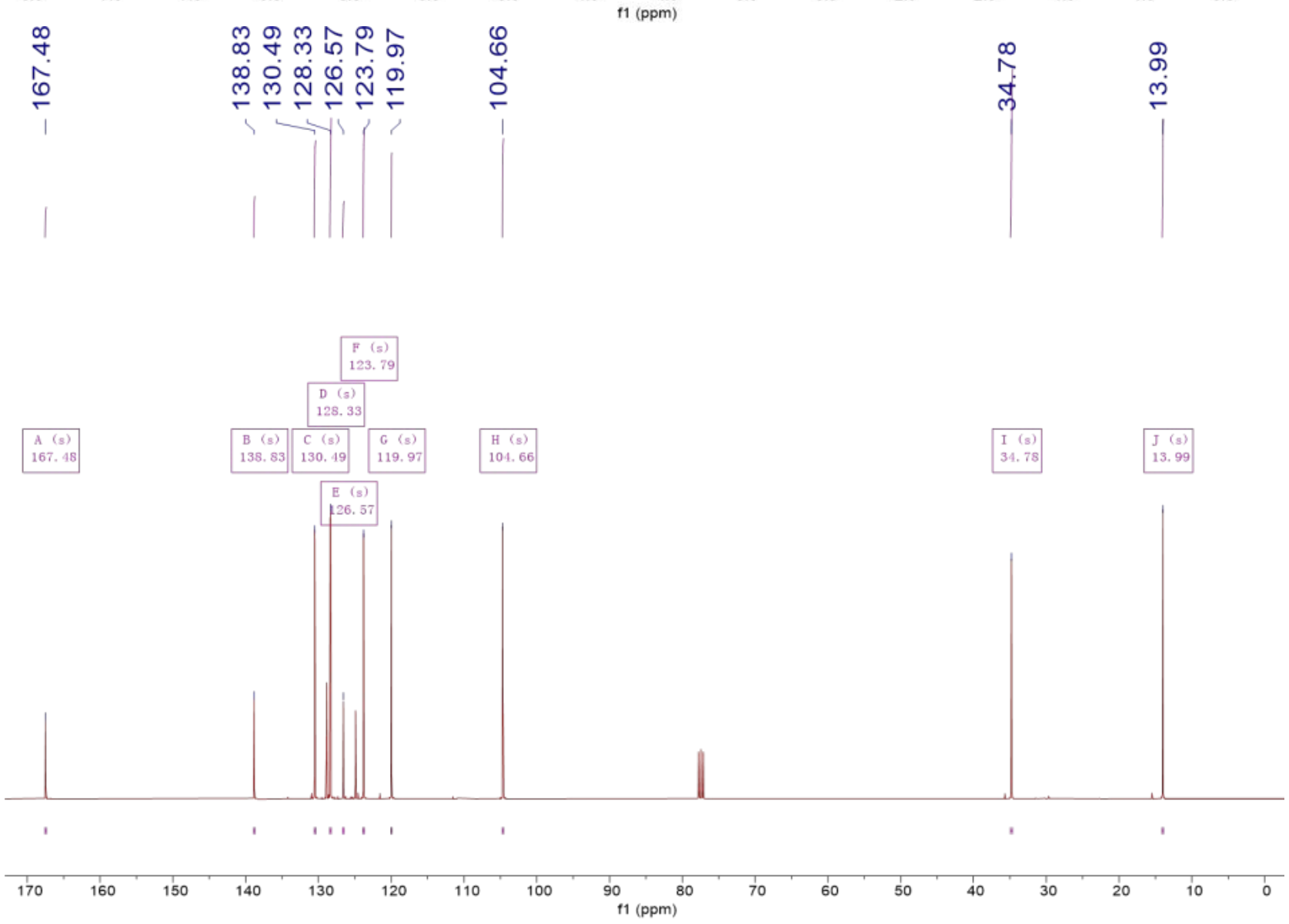

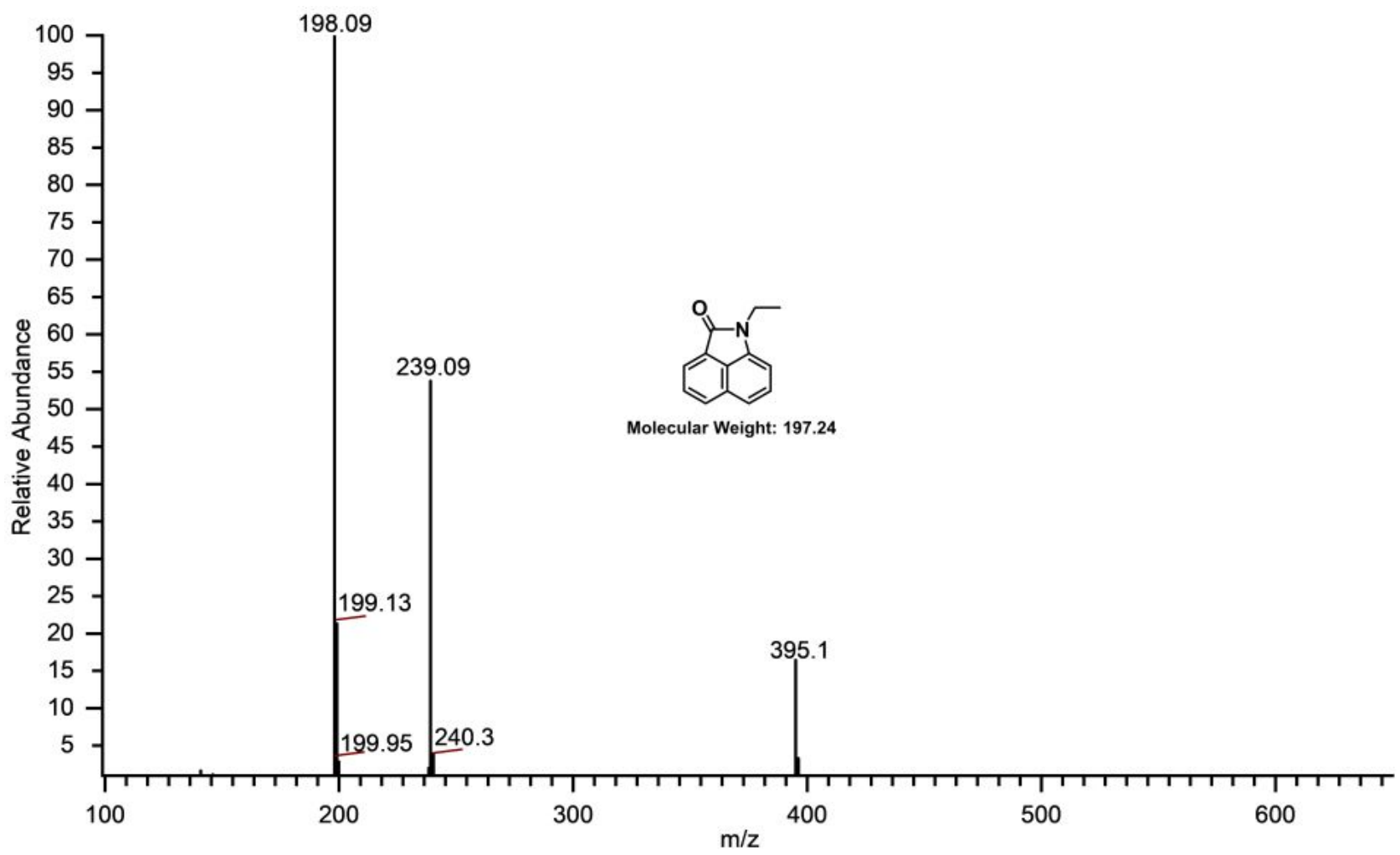

Figure S7. ^{1}H NMR (top), ^{13}C NMR (middle) and ESI-MS (bottom) of compound 4.

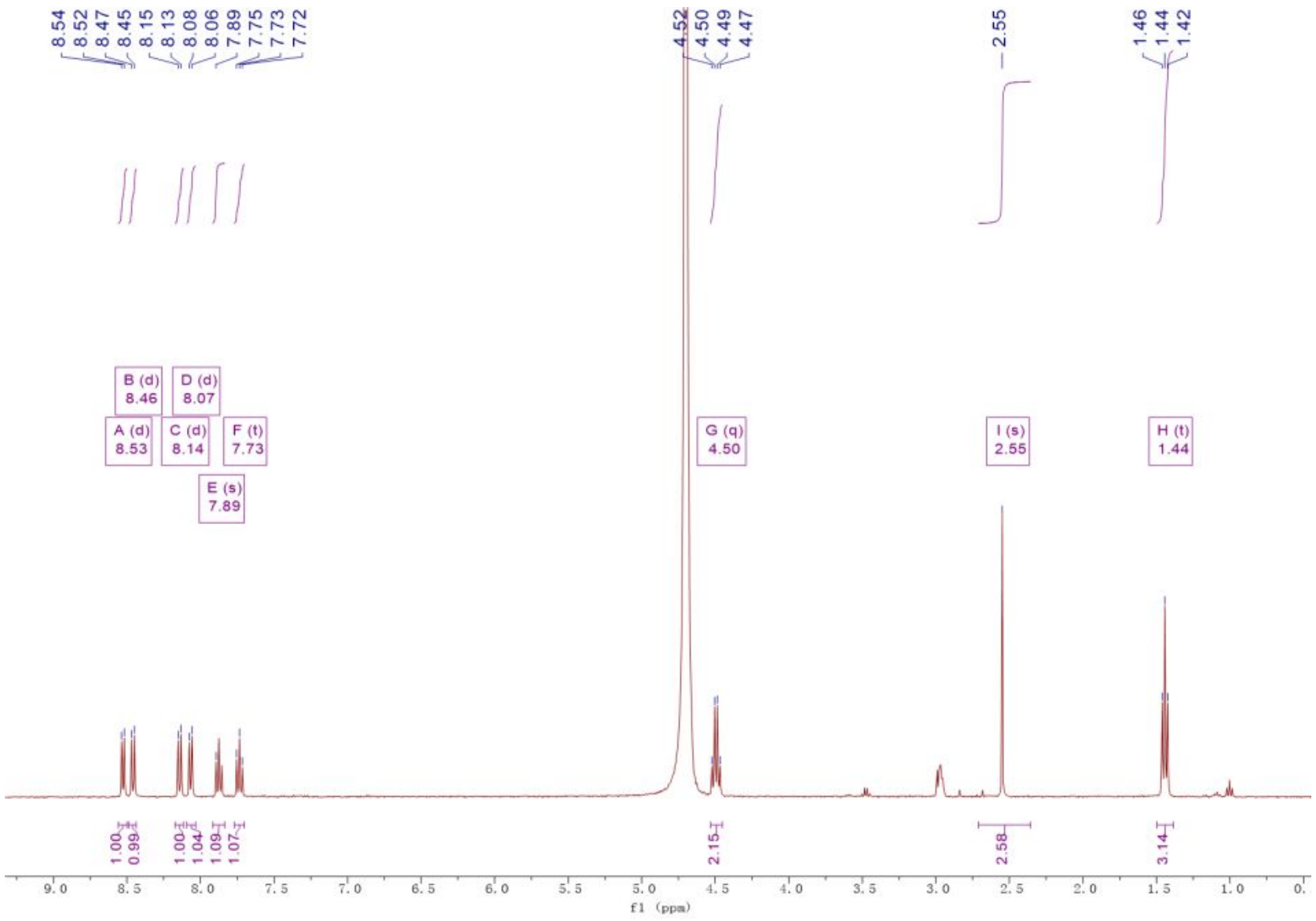

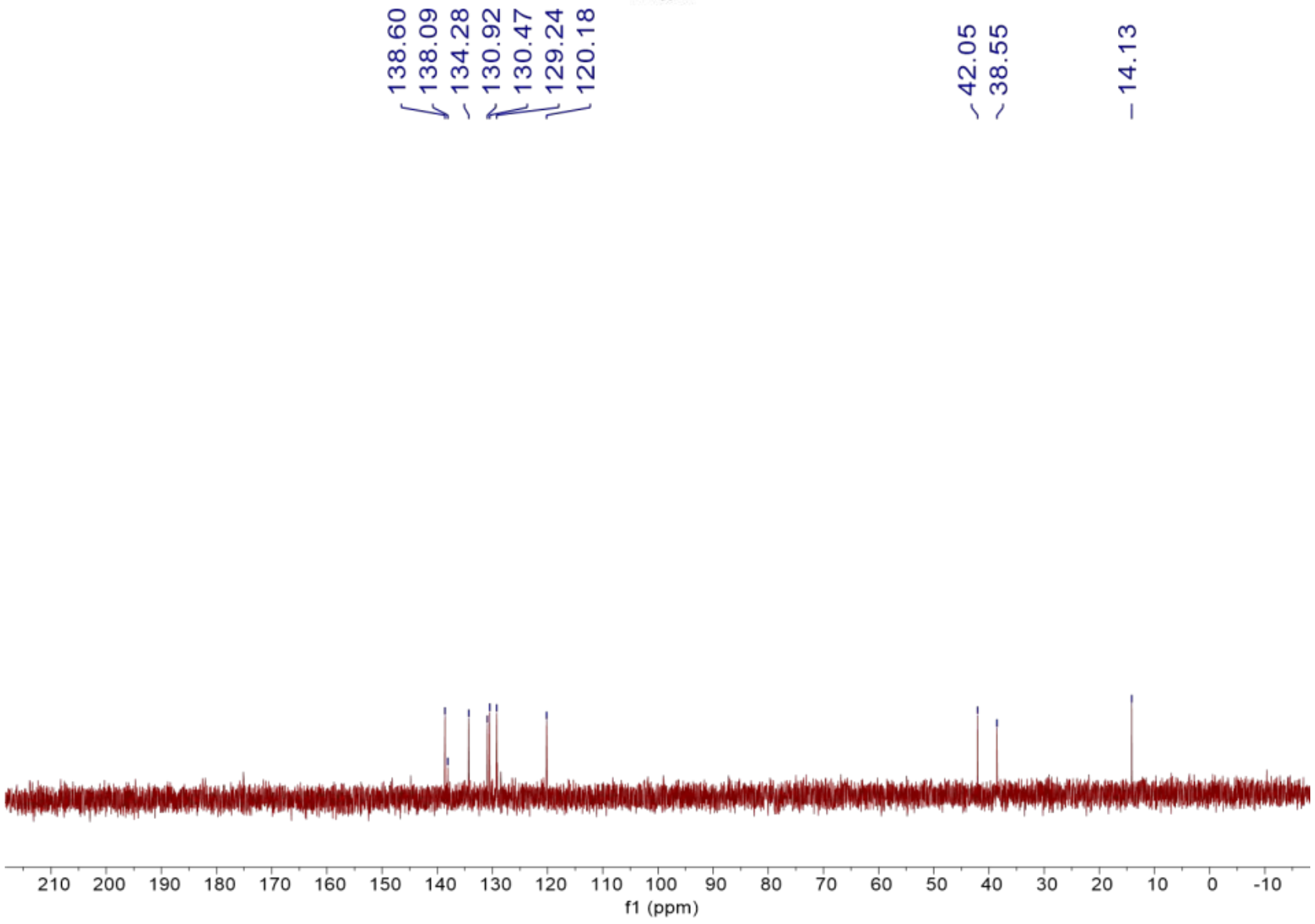

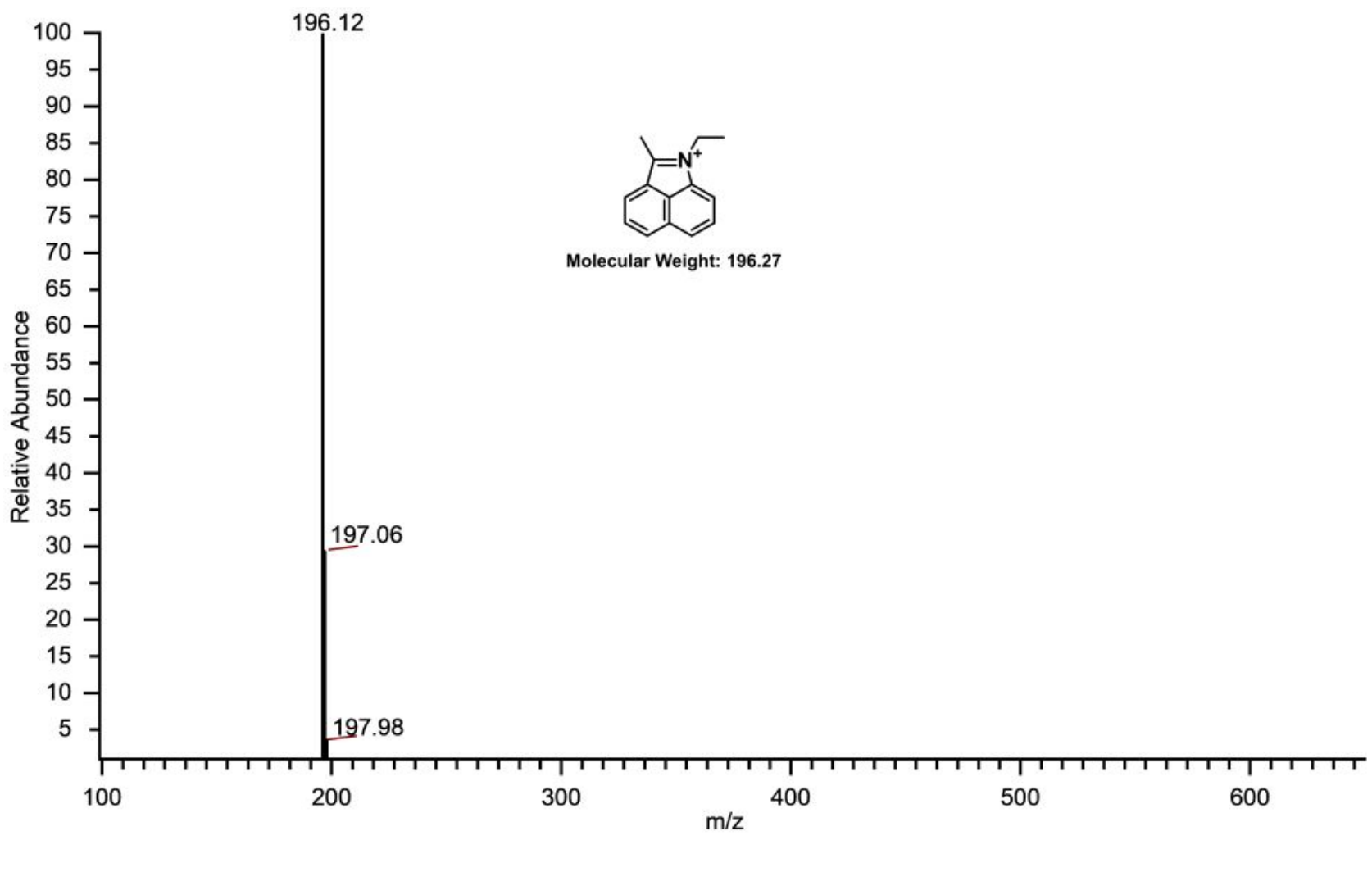

Figure S8. ^{1}H NMR (top), ^{13}C NMR (middle) and ESI-MS (bottom) of compound 5.

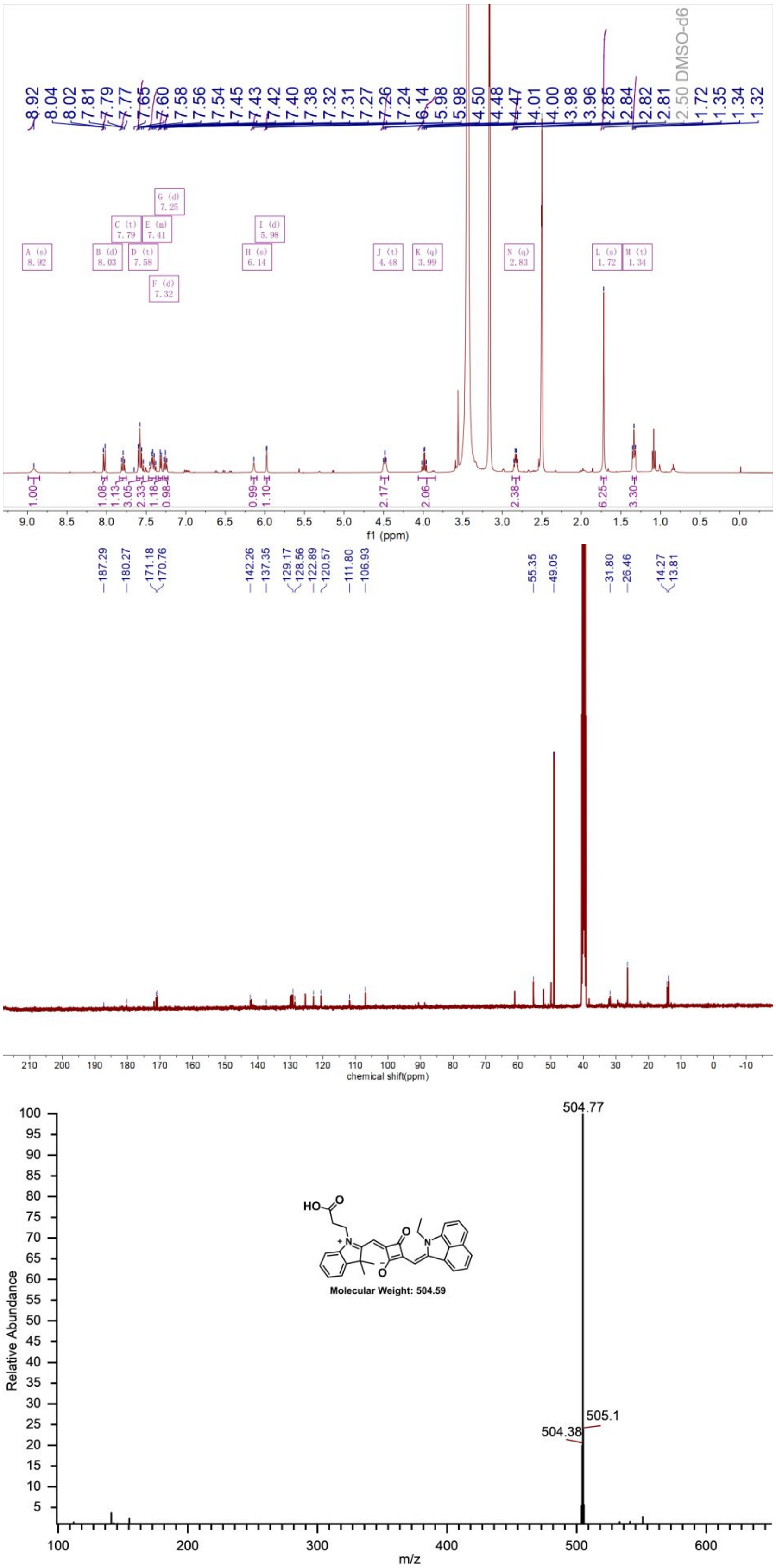

Figure S9. ^{1}H NMR (top), ^{13}C NMR (middle) and ESI-MS (bottom) of SF-BcI.

Table S1. Photophysical properties of the asymmetric squaraine derivatives in EtOH, MeOH, ACN and PBS.

Squaraine derivants	Solvent	λ_{abs}/nm	λ_{em}/nm	$\Delta\lambda$/nm	Φ_f/%	ξ/$M^{-1}cm^{-1}$
SF[a]	EtOH	641	646	5	4.16	39700
	MeOH	632	642	10	2.66	35900
	ACN	632	645	13	3.77	31300
	PBS	625	637	12	0.61	41800
SF-F[b]	EtOH	750	772	22	1.06	42700
	MeOH	747	768	21	0.36	36100
	ACN	760	777	17	0.24	35500
	PBS	703	720	17	0.69	7300
SF-F-S[b]	EtOH	756	790	24	0.66	40500
	MeOH	749	789	40	0.57	42500
	ACN	765	802	37	0.14	46630
	PBS	719	764	45	0.81	12400
SF-BI[a]	EtOH	646	656	10	4.18	48900
	MeOH	643	652	9	2.66	37100
	ACN	646	657	11	1.96	40700
	PBS	654	666	12	2.42	38400
SF-Bcl[b]	EtOH	763	833	70	0.85	38100
	MeOH	755	825	70	0.27	38300
	ACN	719	831	112	0.41	37700
	PBS	736	780	44	0.05	18400

a. Fluorescence quantum yields were calculated with cresyl violet as the reference (Φ_f=0.54, MeOH). b. Fluorescence quantum yields were calculated with IR 780 as the reference (Φ_f=0.17, EtOH).

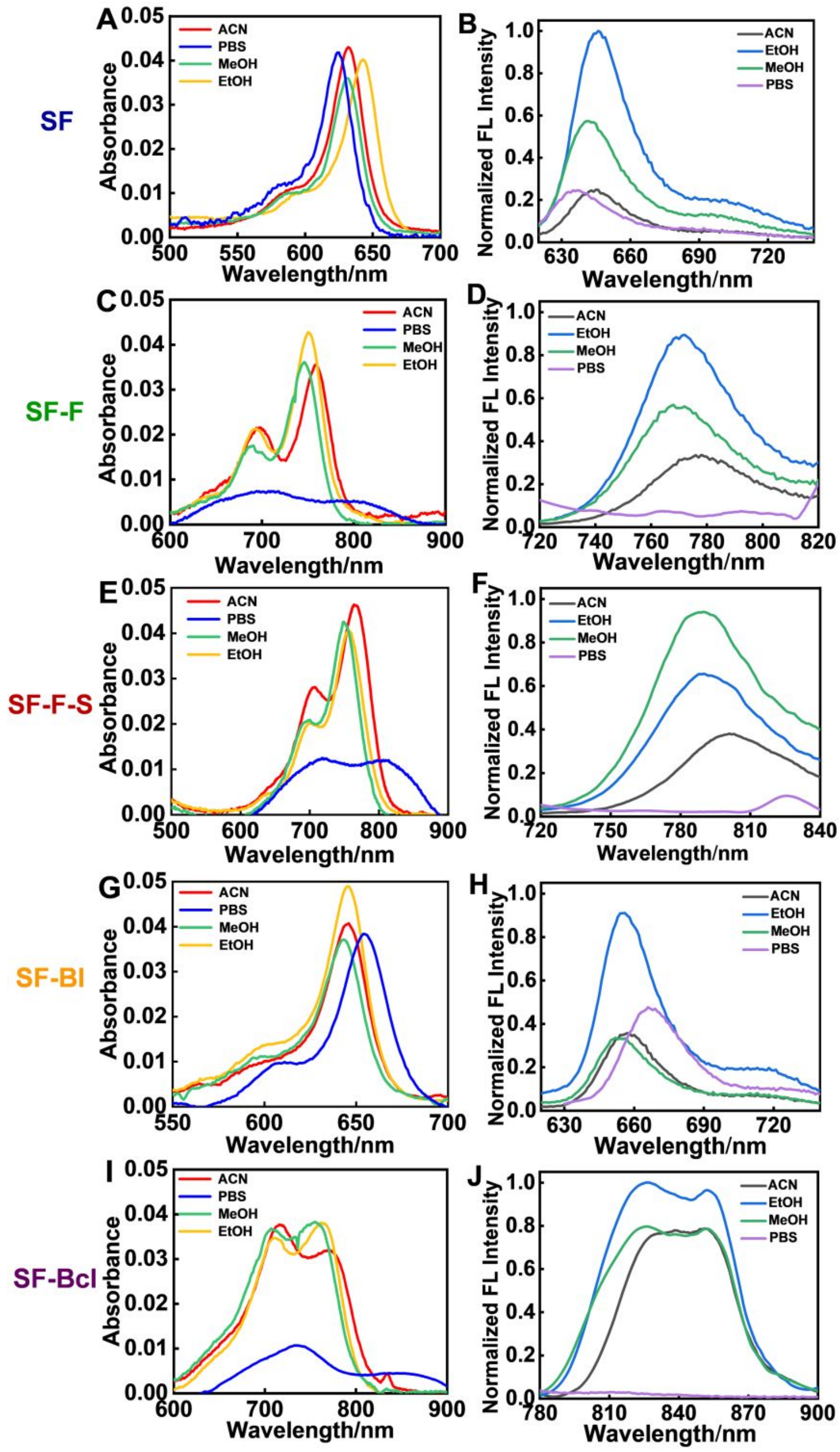

Figure S10. UV-vis-NIR absorption spectra of (A) SF (1 μM), (C) SF-F (1 μM), (E) SF-F-S (1 μM), (G) SF-BI (1 μM) and (I) SF-BcI (1 μM) in different solvent (DMSO, EtOH, ACN and PBS). Normalized fluorescence emission spectra of (B) SF (1 μM), (D) SF-F (1 μM), (F) SF-F-S (1 μM), (H) SF-BI (1 μM) and (J) SF-BcI (1 μM) in different solvents (DMSO, EtOH, ACN and PBS). The maximum of fluorescence intensity of squaraine derivatives was set as 1.0.

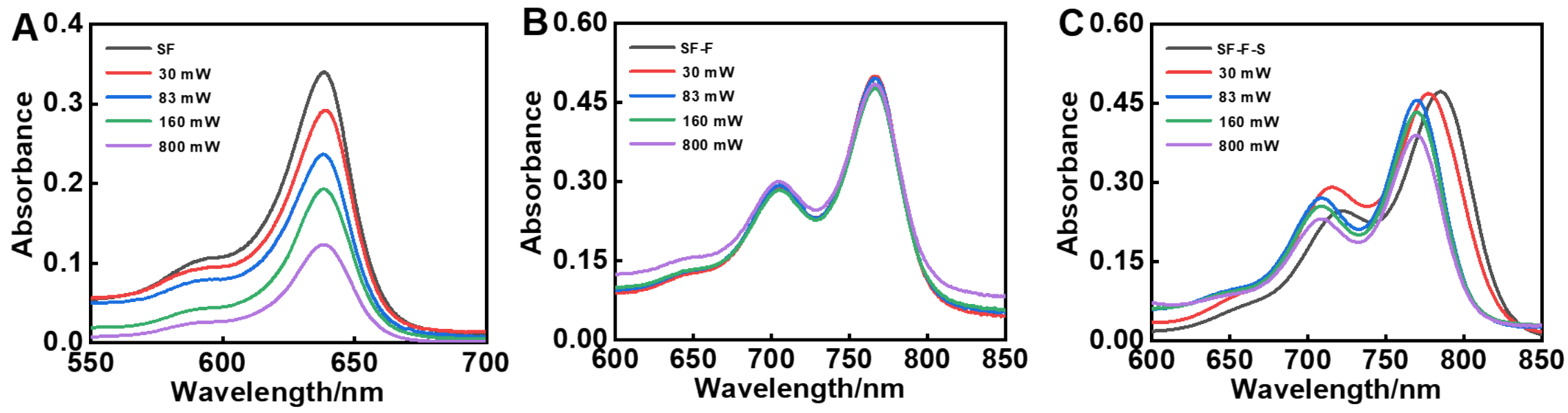

Figure S11. UV-vis-NIR absorption spectra of (A) SF (5 μM), (B) SF-F (5 μM), (C) SF-F-S (5 μM) under the laser illumination at different powers.

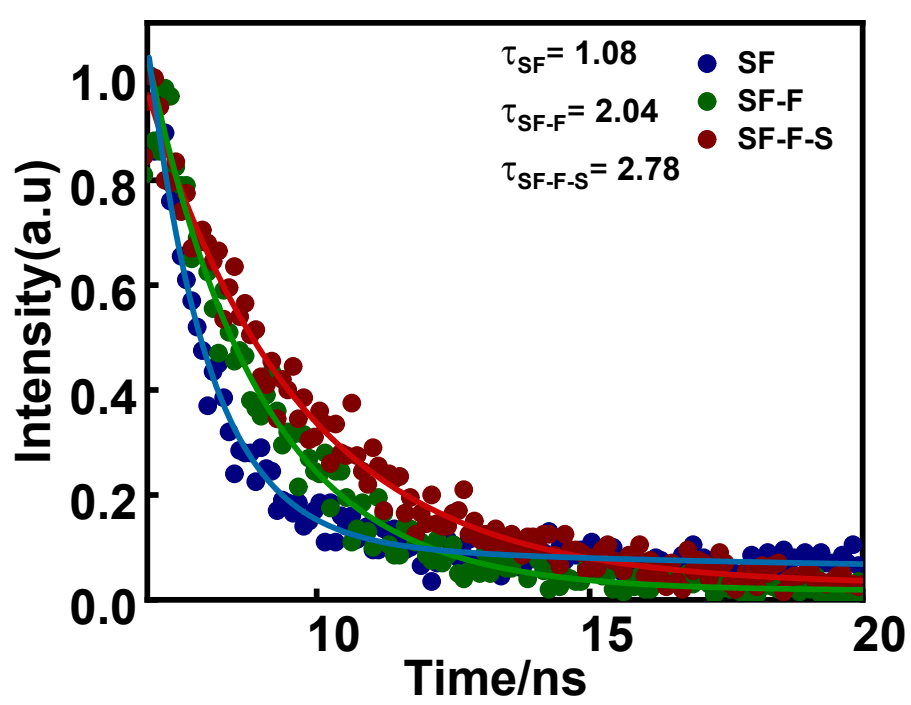

Figure S12. Fluorescent lifetime of SF, SF-F and SF-F-S.

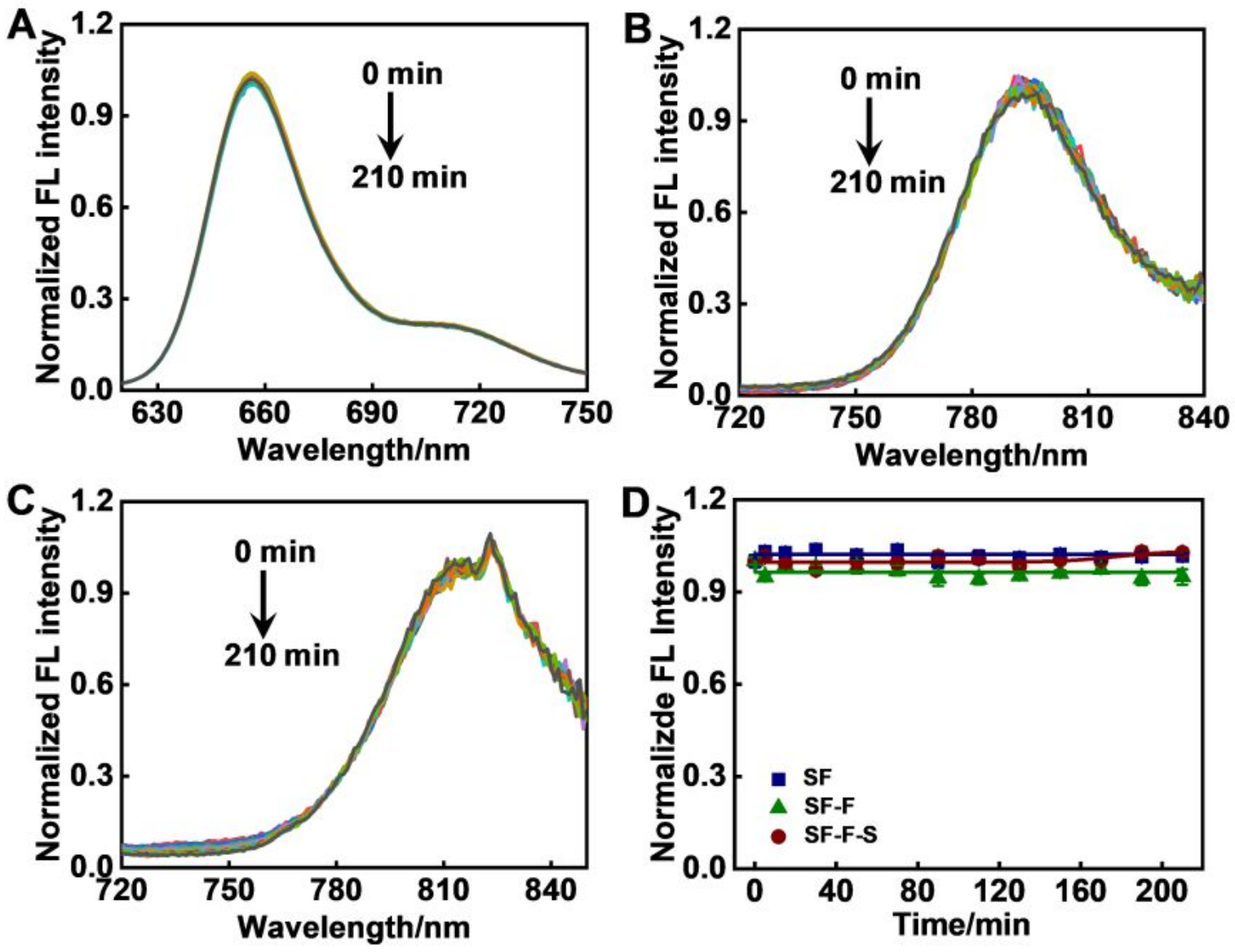

Figure S13. Normalized fluorescence emission spectra of (A) SF (5 μM), (B) SF-F (5 μM), (C) SF-F-S (5 μM) without laser illumination at different time points. (D) Normalized fluorescence intensity of SF (blue line), SF-F (green line) and SF-F-S (red line) at different time points, and the fluorescence intensity of squaraine derivatives at 0 min was set as 1.0.

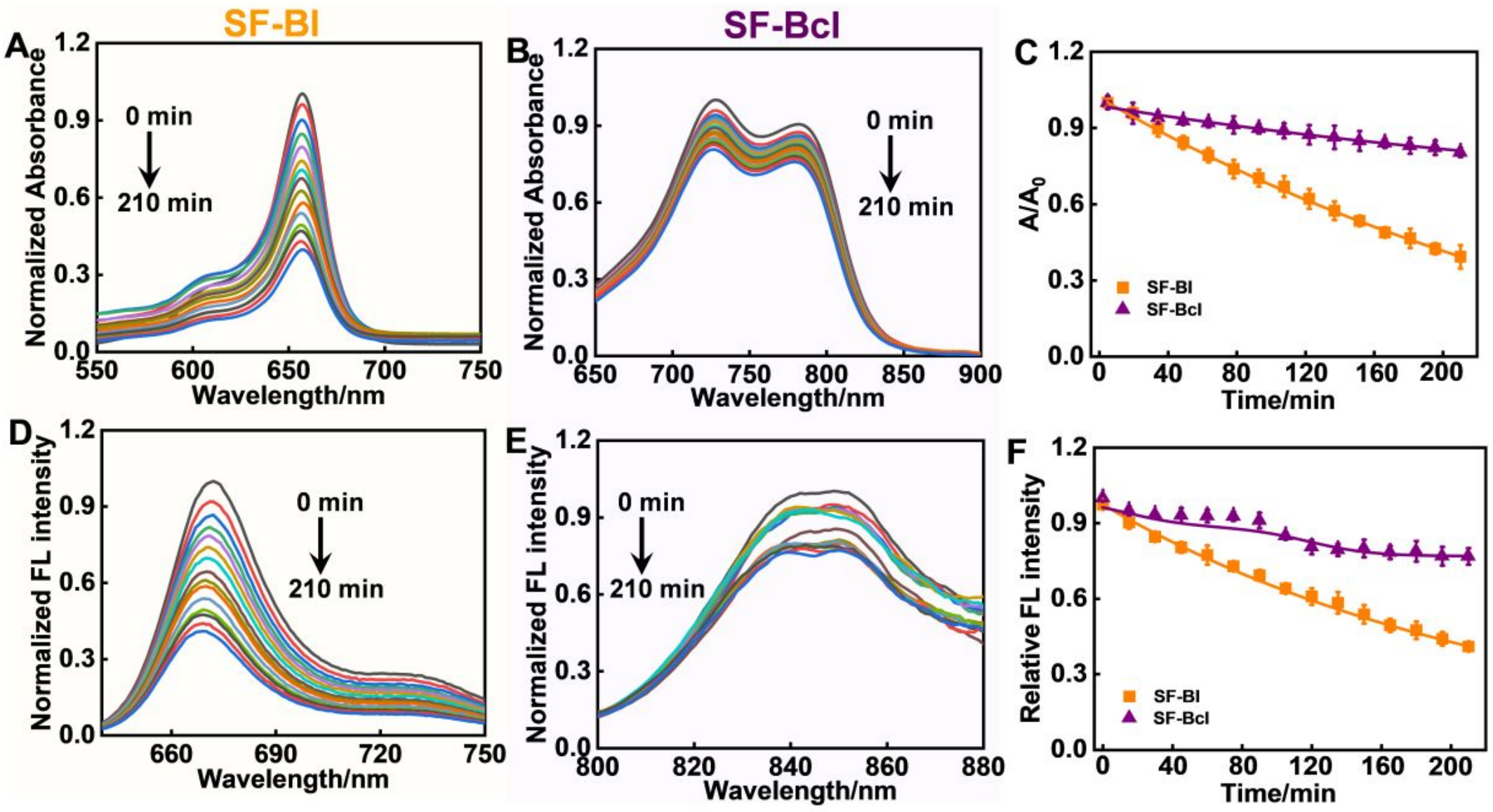

Figure S14. UV-vis-NIR absorption spectra of (A) SF-BI, (B) SF-BcI in trifluoroethanol under laser illumination (83 mW) at different time points (0, 15, 30, 45, 60, 75, 90, 105, 120, 135, 150, 165, 180, 195 and 210 min). (C) Changes of relative absorption for the squaraine derivatives at different time points. Fluorescence emission spectra of (D) SF-BI, (E) SF-BcI in trifluoroethanol under laser illumination (83 mW) at different time points (0, 15, 30, 45, 60, 75, 90, 105, 120, 135, 150, 165, 180, 195 and 210 min). (F) Fluorescence ratio changes of squaraine derivatives at different time points.

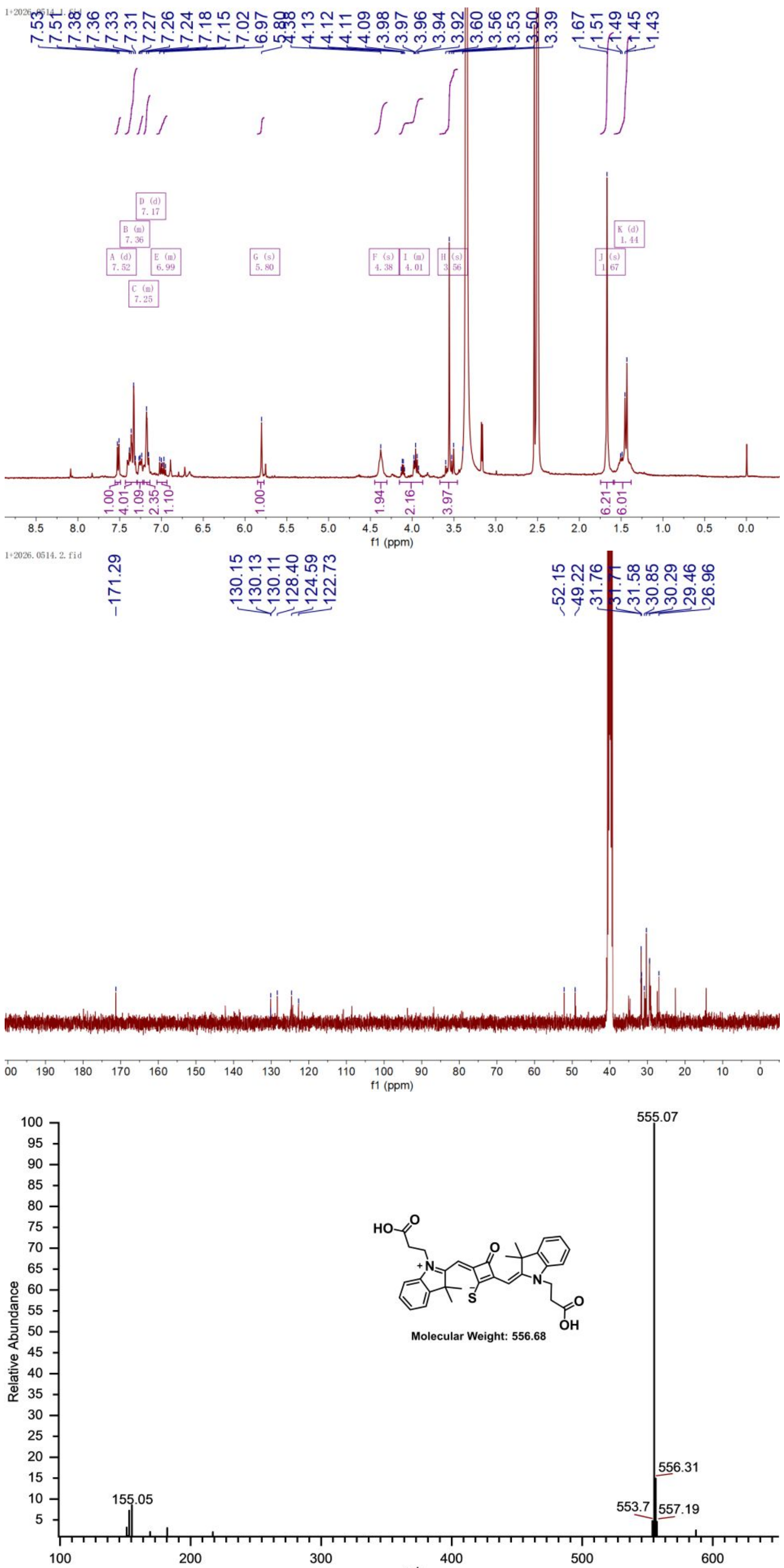

Figure S15. ^{1}H NMR (top), ^{13}C NMR (middle) and ESI-MS (bottom) of SF-S.

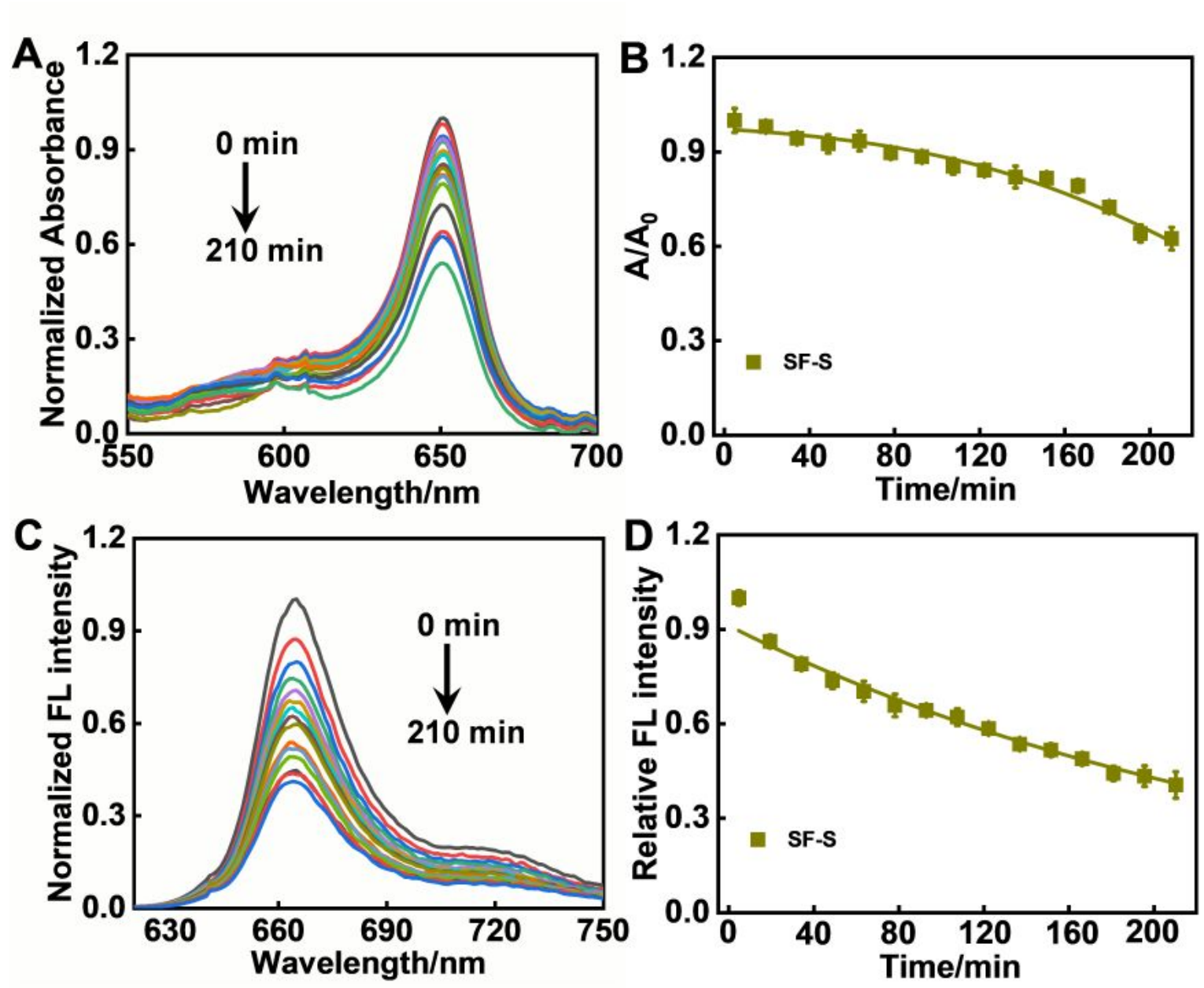

Figure S16. (A) UV-vis-NIR absorption and (B) fluorescence emission spectra of SF-S in trifluoroethanol under laser illumination (83 mW) at different time points (0, 15, 30, 45, 60, 75, 90, 105, 120, 135, 150, 165, 180, 195 and 210 min). (C) Changes of relative absorption and fluorescence intensity of SF-S at different time points.

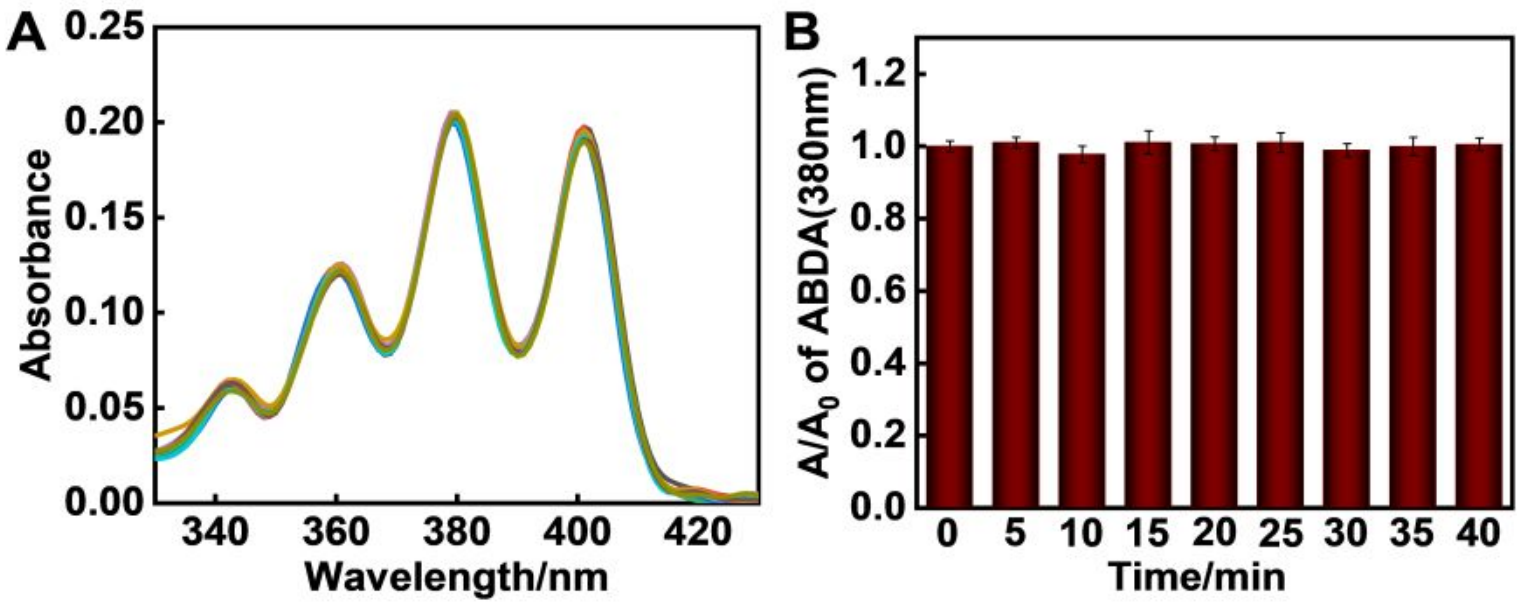

Figure S17. (A) UV-vis absorption spectra of ABDA (15 μM) under the laser illumination (83 mW) at different time points. (B) Relative changes of absorbance at 380 nm of ABDA under the laser illumination. Error bars were calculated from three repeated measurements.

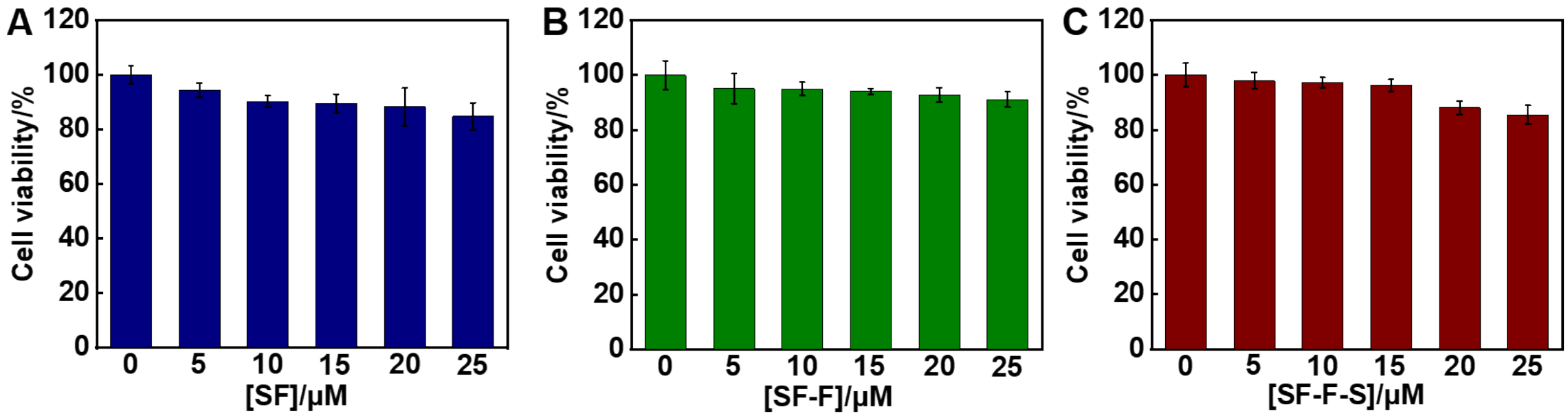

Figure S18. Survival rate of HepG2 cells after being treated with different concentrations (0, 5, 10, 15, 20 and 25 μM) of (A) SF, (B) SF-F, (C) SF-F-S. Error bars were calculated from three repeated measurements.

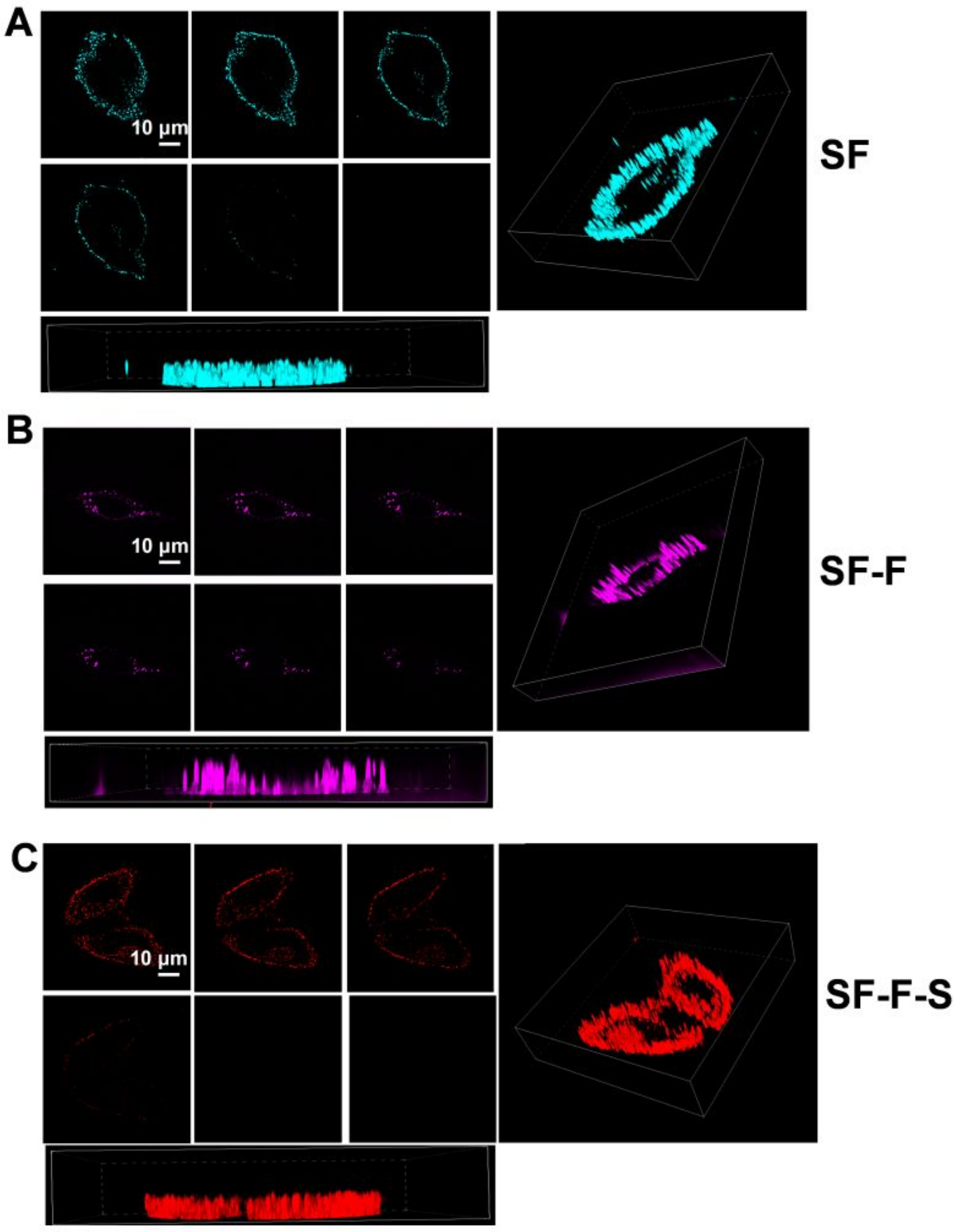

Figure S19. 3D-SIM images of HepG2 cells incubated with (A) SF (10 μM, λ_{ex} = 640 nm, λ_{em} = 663-738 nm), (B) SF-F (10 μM, λ_{ex} = 640 nm, λ_{em} = 663-738 nm), (C) SF-F-S (10 μM, λ_{ex} = 640 nm, λ_{em} = 663-738 nm). Scale bars: 10 μm.

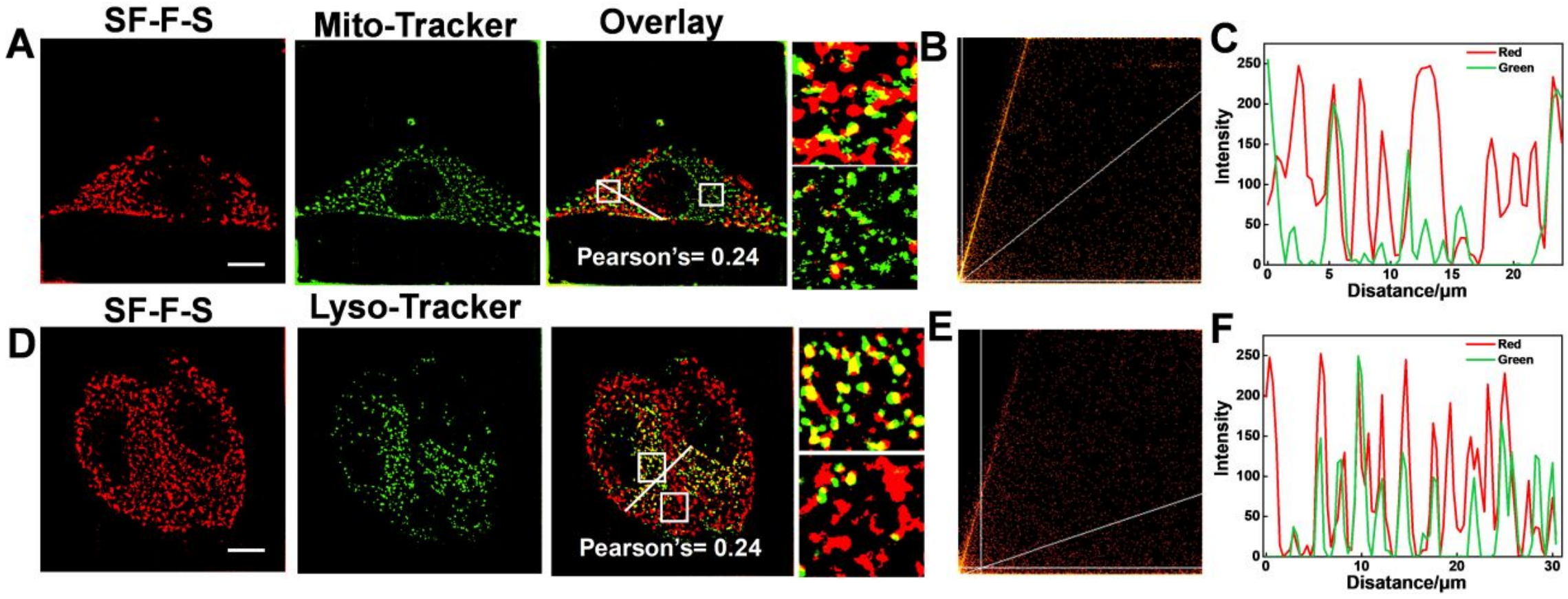

Figure S20. SIM images of living cells incubated with SF-F-S (10 μM, λ_{ex} = 640 nm, λ_{em} = 663-738 nm) and further being treated with (A) Mito-Tracker Green (5 μM, λ_{ex} = 488 nm, λ_{em} = 500-545 nm) and (B) Lyso-Tracker Green (5 μM, λ_{ex} = 488 nm, λ_{em} = 500-545 nm). (B, E) Intensity scatter plot of two channels. (C, F) Intensity profile of ROI across the HepG2 cells incubated with SF-F and organellar tracker. Scale bars: 10 μm.

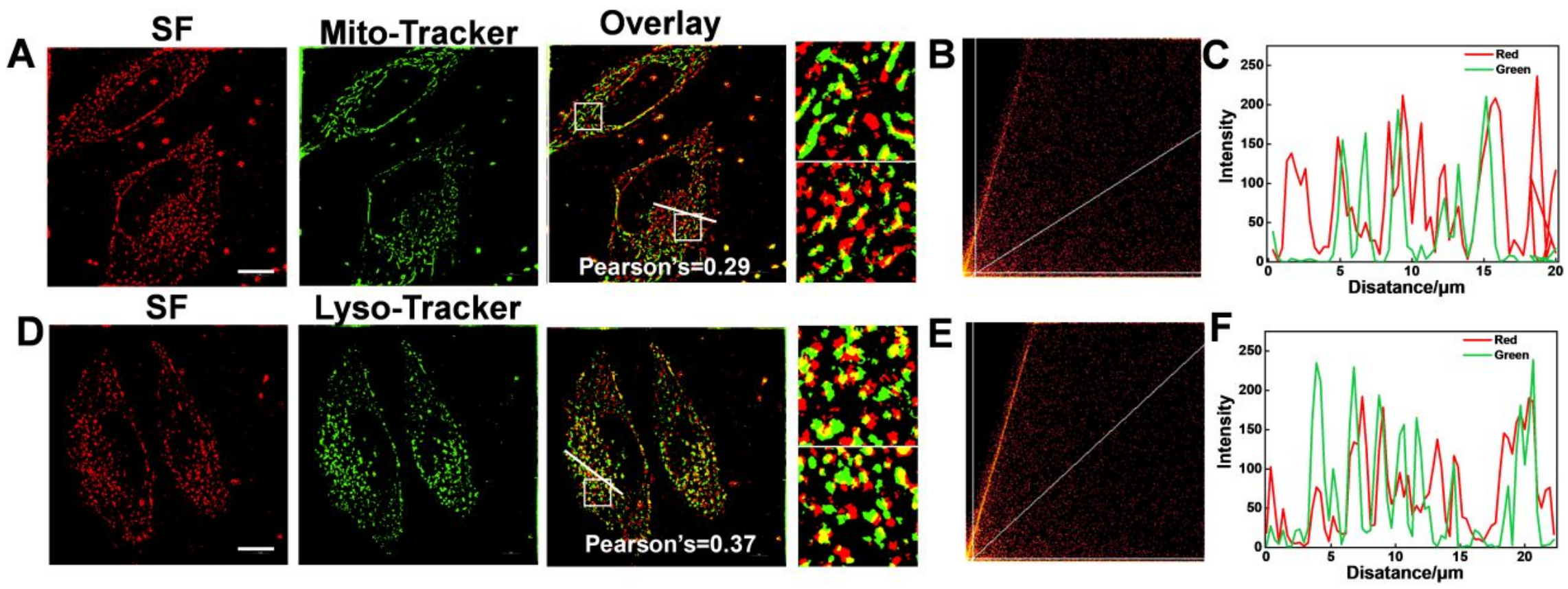

Figure S21. SIM images of living cells incubated with SF (10 μM, λ_{ex} = 640 nm, λ_{em} = 663-738 nm) and further being treated with (A) Mito-Tracker Green (5 μM, λ_{ex} = 488 nm, λ_{em} = 500-545 nm) and (B) Lyso-Tracker Green (5 μM, λ_{ex} = 488 nm, λ_{em} = 500-545 nm). (B, E) Intensity scatter plot of two channels. (C, F) Intensity profile of ROI across the HepG2 cells incubated with SF-F and organellar tracker. Scale bars: 10 μm.

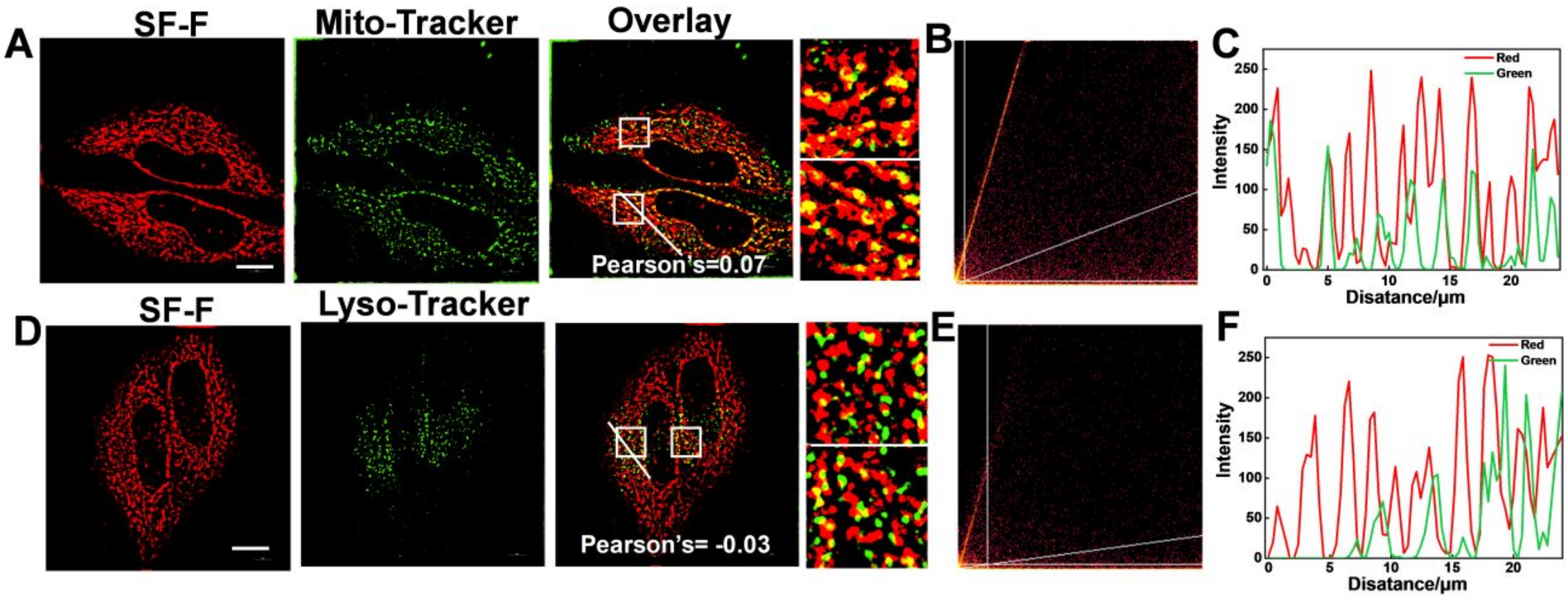

Figure S22. SIM images of living cells incubated with SF-F (10 μM, λ_{ex} = 640 nm, λ_{em} = 663-738 nm) and further being treated with (A) Mito-Tracker Green (5 μM, λ_{ex} = 488 nm, λ_{em} = 500-545 nm) and (B) Lyso-Tracker Green (5 μM, λ_{ex} = 488 nm, λ_{em} = 500-545 nm). (B, E) Intensity scatter plot of two channels. (C, F) Intensity profile of ROI across the HepG2 cells incubated with SF-F and organellar tracker. Scale bars: 10 μm.

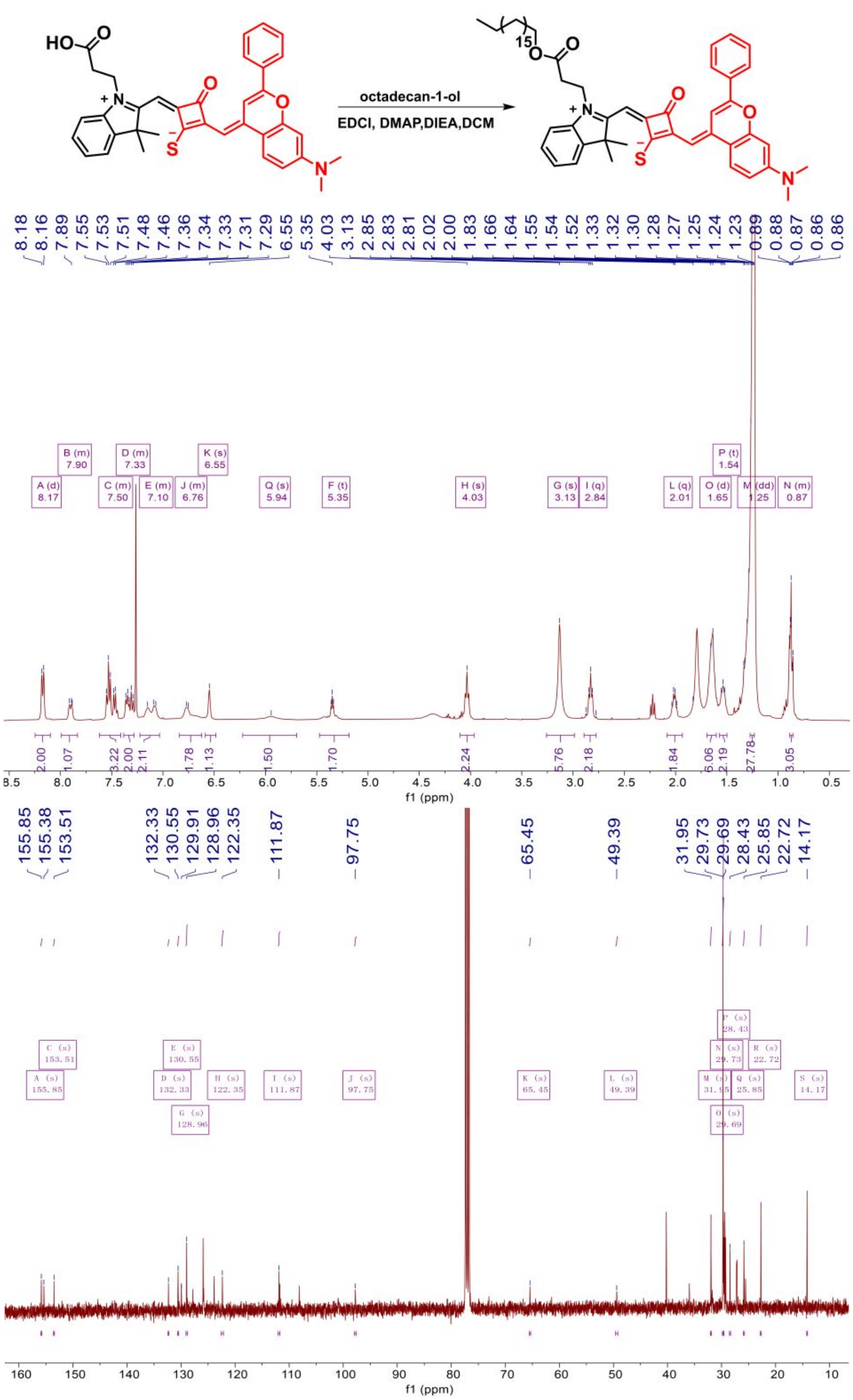

Figure S23. Synthesis of Mito-SF-F-S with SF-F-S (top), and the ^{1}H NMR (middle) and ^{13}C NMR (bottom) of Mito-SF-F-S.

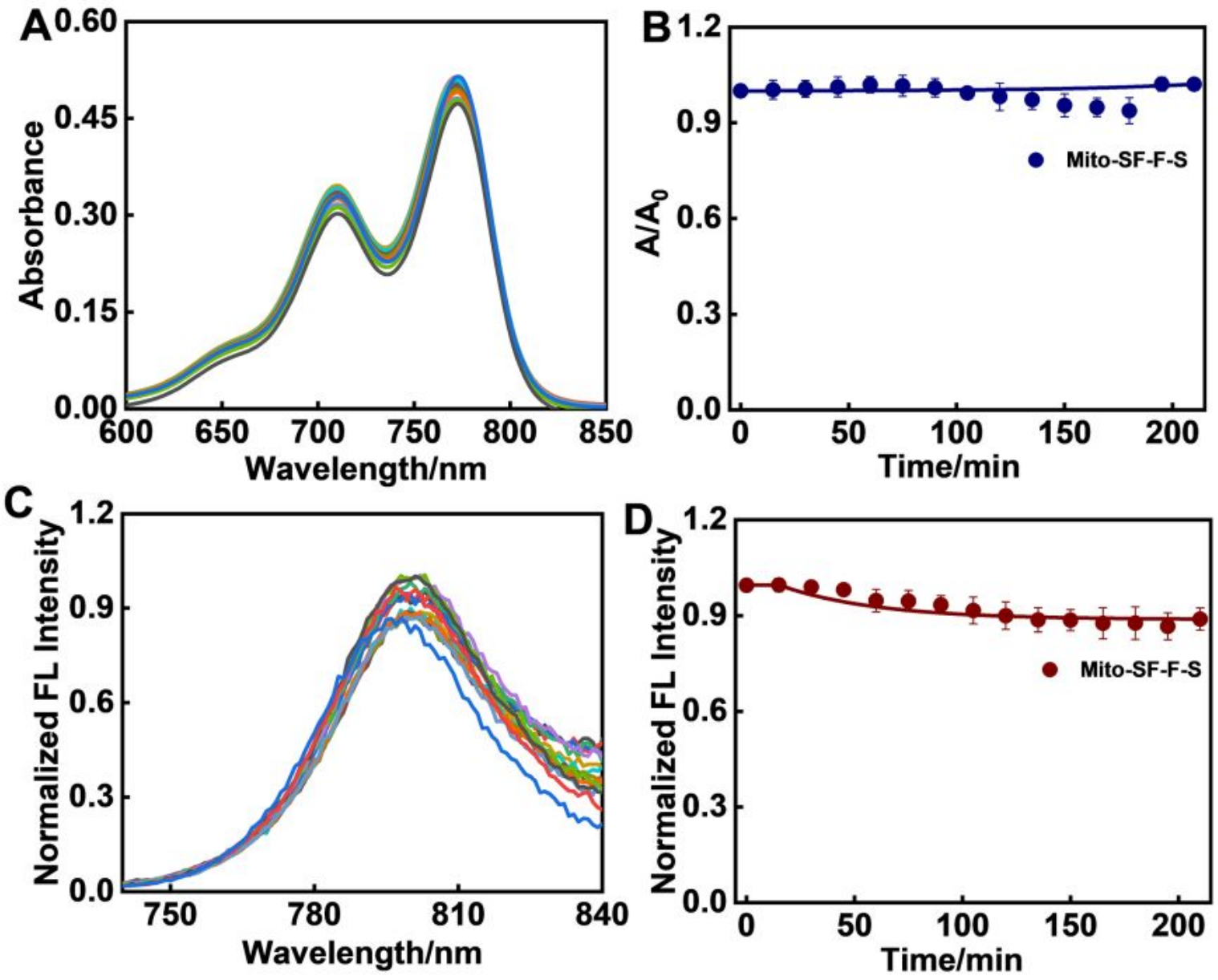

Figure S24. (A) UV-vis-NIR absorption spectra and (C) normalized fluorescence emission spectra of Mito-SF-F-S (5 μM) under the laser illumination (83 mW) at different time points. (B, D) The corresponding change of relative absorption value and fluorescence ratio of Mito-SF-F-S (5 μM) under the laser illumination (83 mW) at different time points. Error bars were calculated from three repeated measurements.

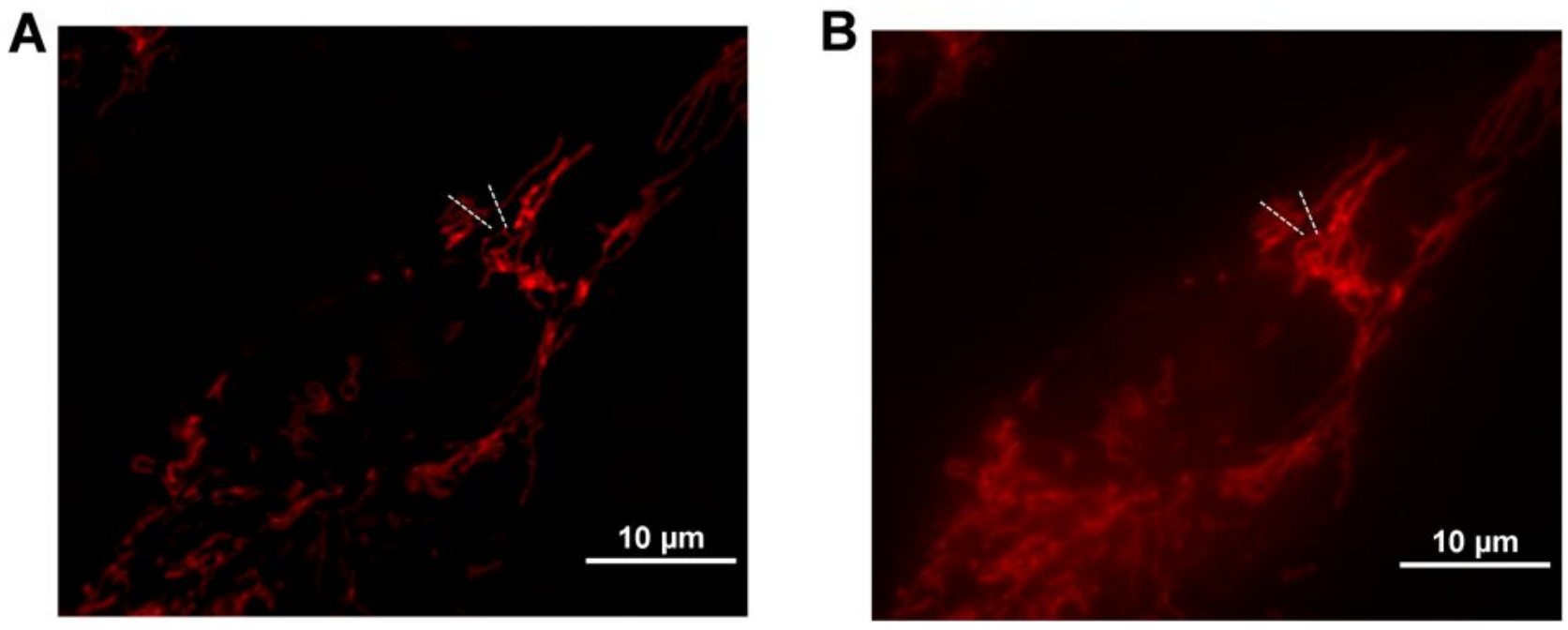

Figure S25. (A) SIM and (B) wide-field images of live cells incubated with Mito-SF-F-S (10 μM).

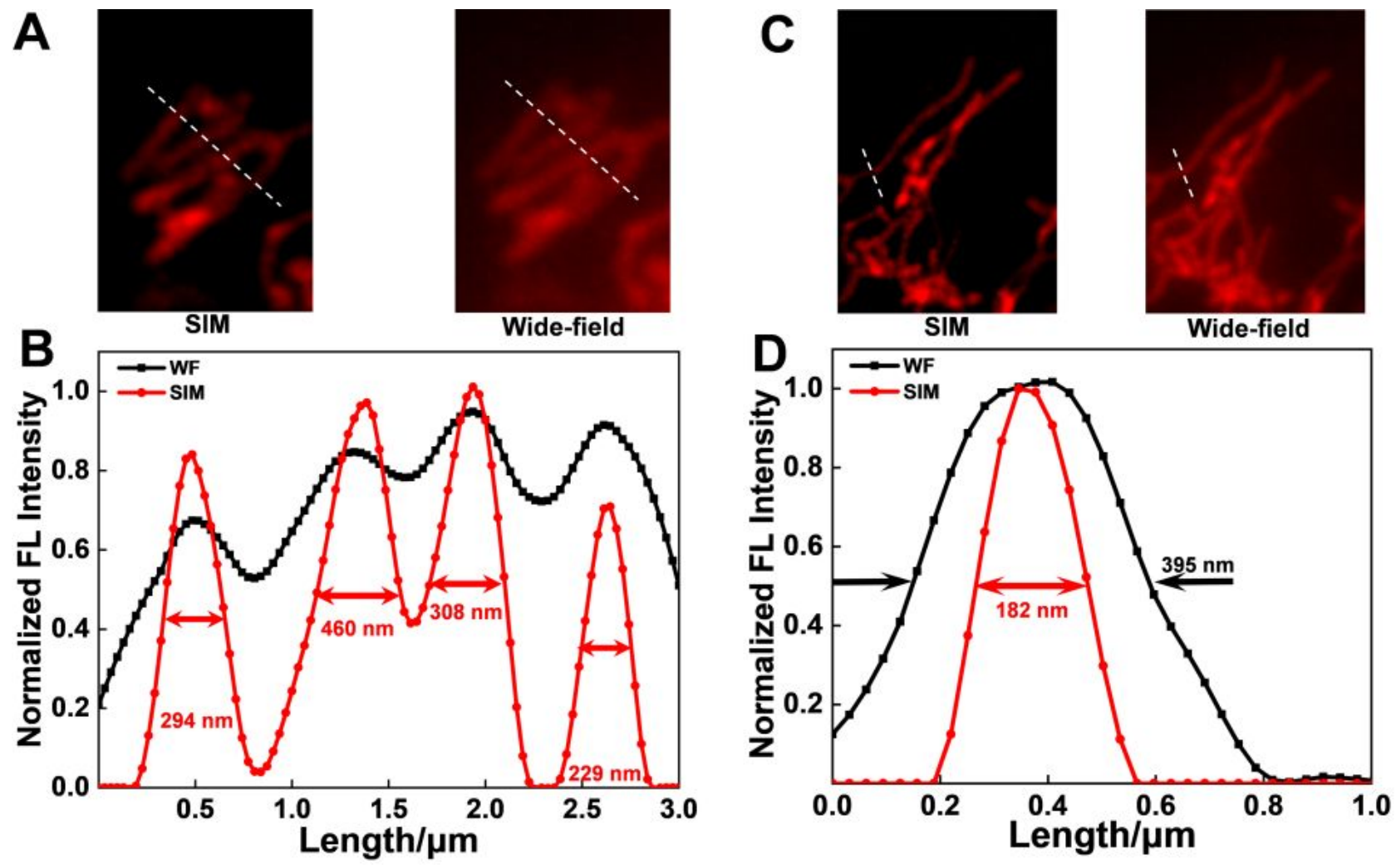

Figure S26. (A, C) Enlarged SIM and wide-field images of the region of interest shown in Figure S25. (B, D) Normalized fluorescence intensity profile along the white dashed line shown in (A) and (C).

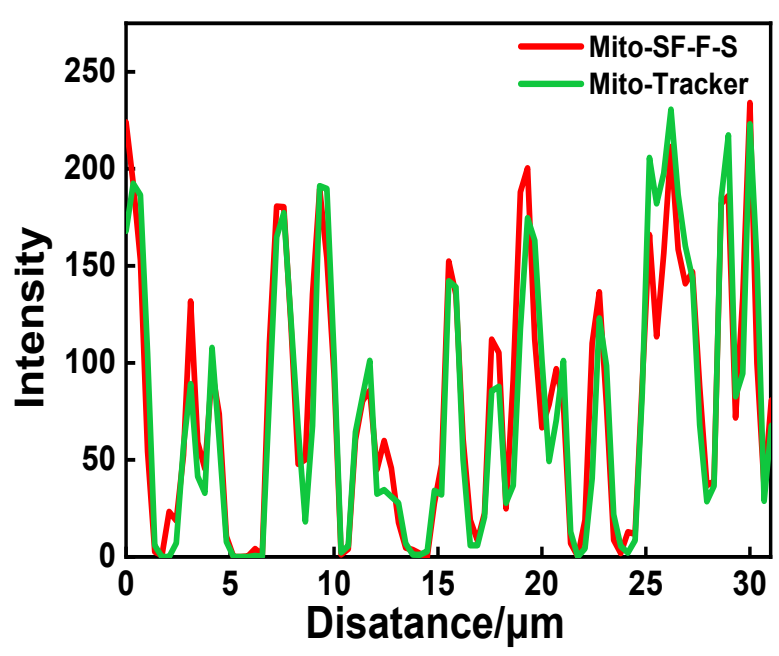

Figure S27. Intensity profile of linear region of interest (ROI 1) across the cells in the merged image.

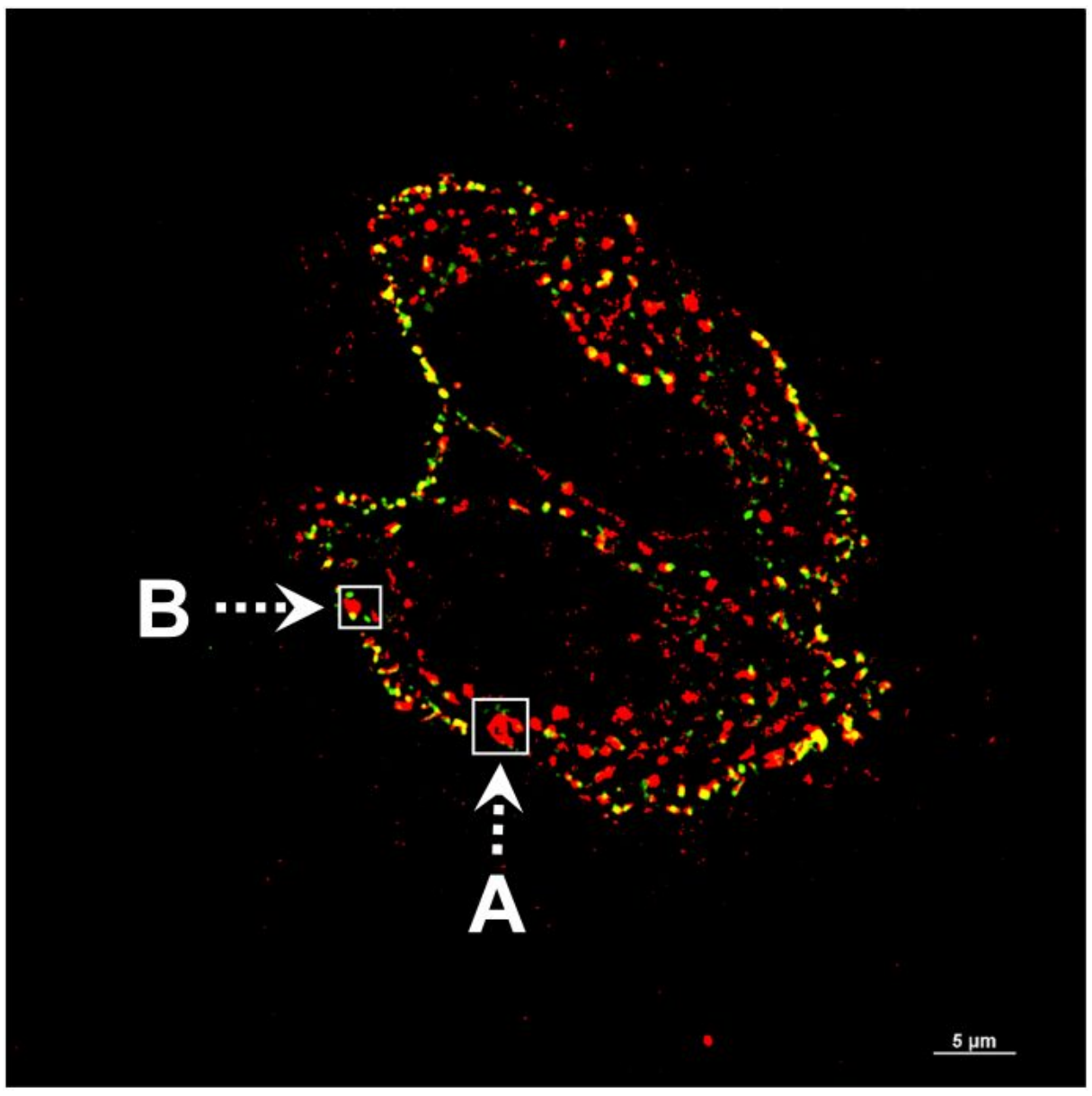

Figure S28. SIM image of living cells incubated with lysosomal tracker (5 μM, λ_{ex} = 488 nm, λ_{em} = 500-545 nm) and Mito-SF-F-S (10 μM, λ_{ex} = 640 nm, λ_{em} = 663-738 nm) and then stimulated with CCCP (10 μM). The site (A) and site (B) are shown in Figure 6 (G) and (I).

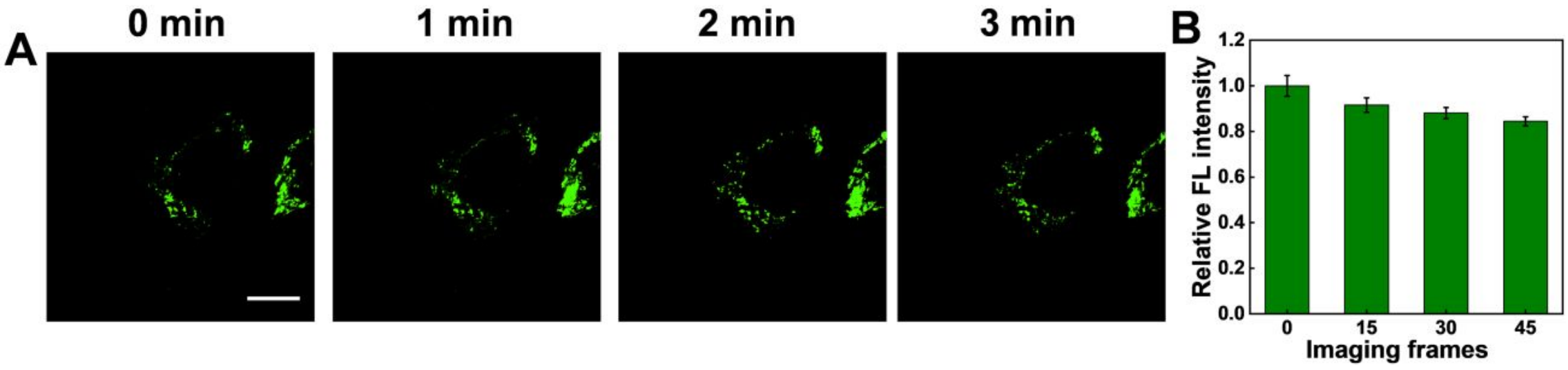

Figure S29. (A) SIM images of living cells incubated with Lyso-Tracker Green (5 μM, λ_{ex} = 488 nm, λ_{em} = 500-545 nm) and imaged at different time points (0, 1, 2 and 3 min). Scale bar: 10 μm. (B) Changes of relative fluorescence intensity of live cells incubated with Lyso-Tracker Green under continuous imaging mode, and the fluorescence intensity of cells at 0 min was set as 1.0. Error bars were calculated from three repeated measurements.

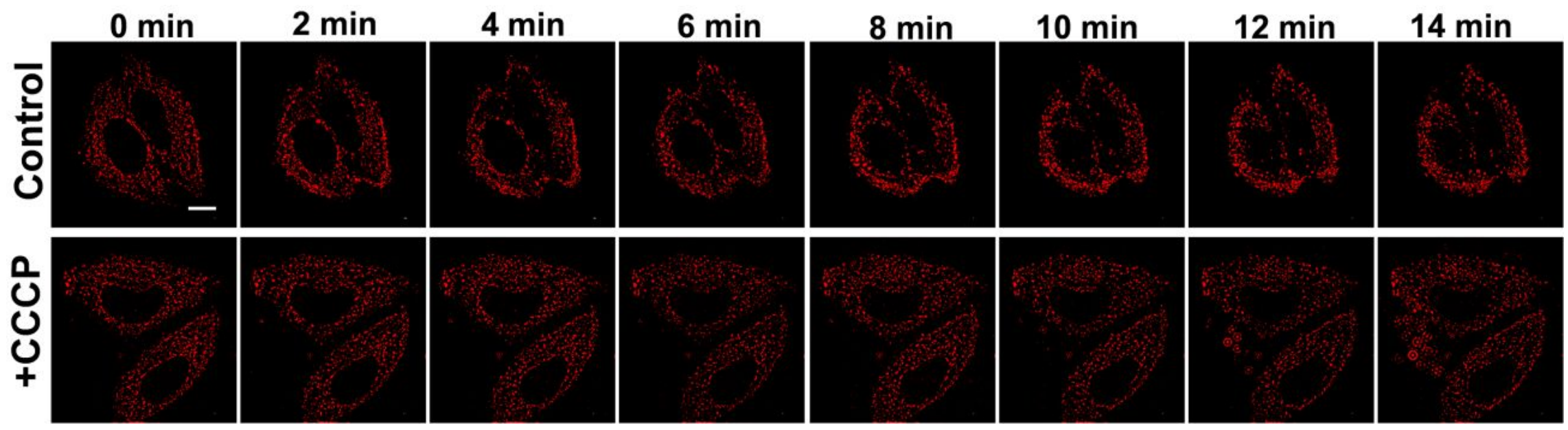

Figure S30. SIM images of living cells incubated with Mito-SF-F-S (10 μM, λ_{ex} = 640 nm, λ_{em} = 663-738 nm) in the absence (above) and presence (below) CCCP (10 μM) and imaged at different time points (0, 2, 4, 6, 8, 10, 12 and 14 min). Scale bar: 10 μm.

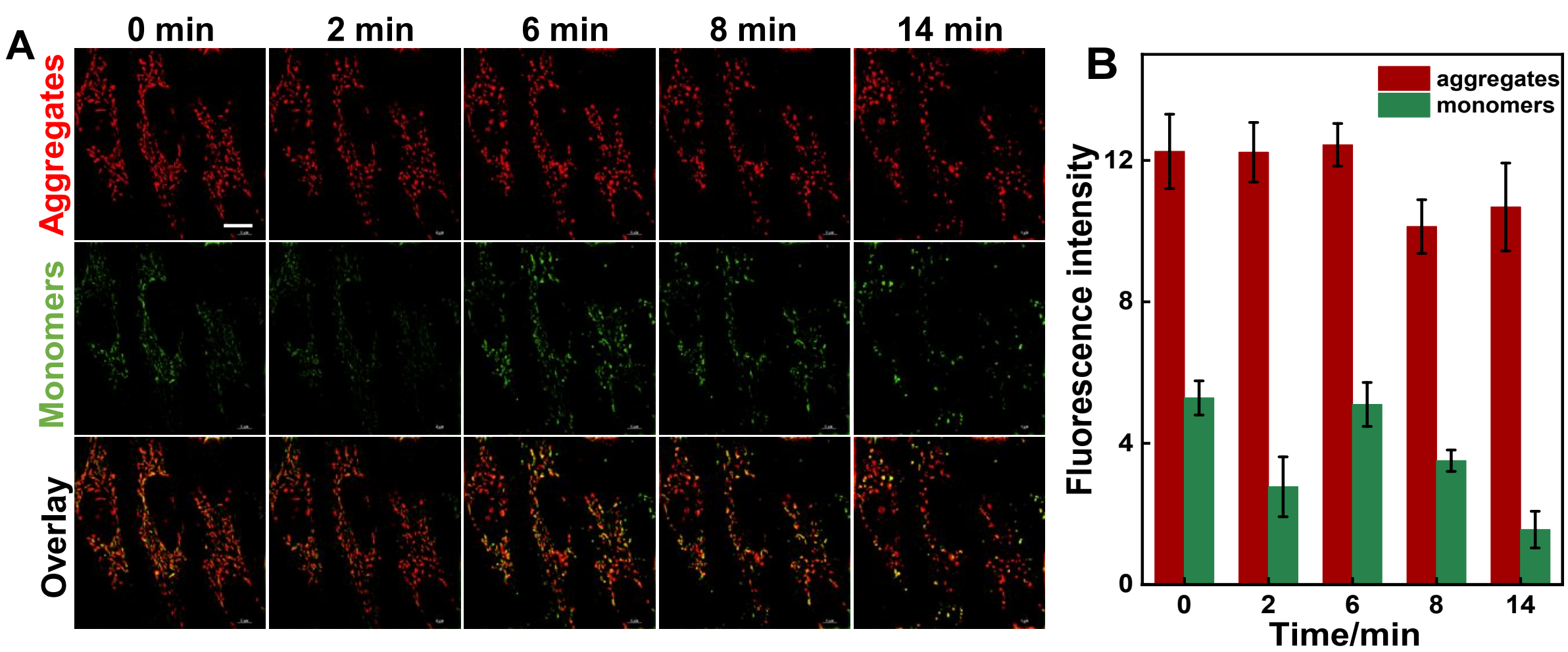

Figure S31. (A) SIM images of living cells incubated with JC-1 (10 μM, Monomers, λ_{ex} = 488 nm, λ_{em} = 500-545 nm; Aggregates, λ_{ex} = 561 nm, λ_{em} = 570-640 nm) and imaged at different time points (0, 2, 6, 8 and 14 min). (B) Fluorescence intensity of cells shown in (A). Scale bar: 10 μm. Error bars were calculated from three repeated measurements.

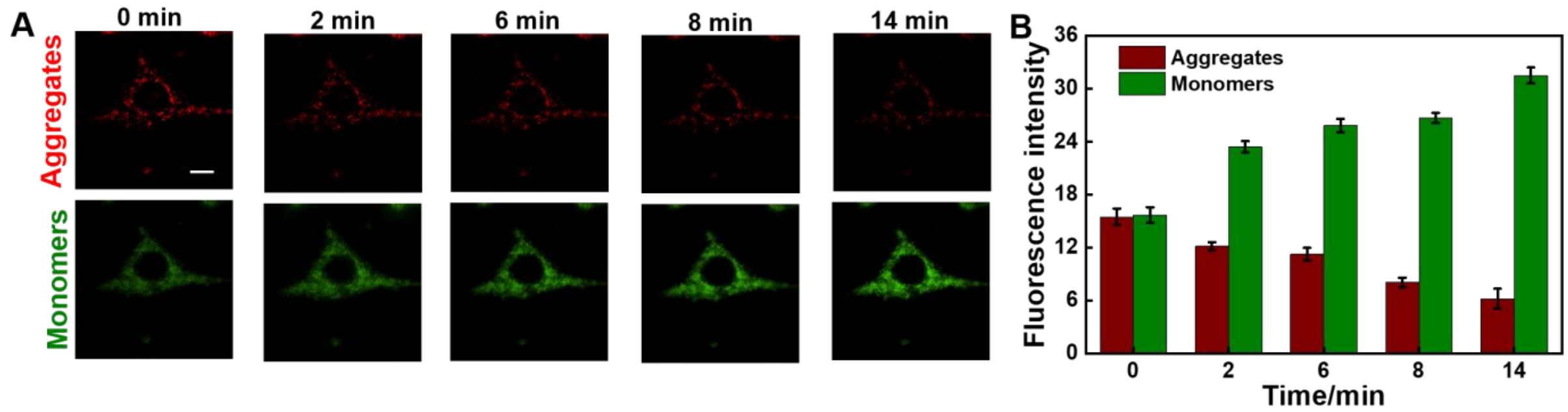

Figure S32. (A) SIM images of live cells incubated JC-1 (10 μM, Monomers, λ_{ex} = 488 nm, λ_{em} = 500-545 nm; Aggregates, λ_{ex} = 561 nm, λ_{em} = 570-640 nm) in the presence of CCCP (10 μM) at different time points (0, 2, 4, 6, 8 and 14 min) (50 W/cm^2). (B) Change of fluorescence intensity of live cells shown in (A). Error bars were calculated from three repeated measurements.

References

(1) Zhou, Y.; Yang, S.; Xiao, Y.; Zou, Z.; Qing, Z.; Liu, J.; Yang, R. Cytoplasmic Protein-Powered In Situ Fluorescence Amplification for Intracellular Assay of Low-Abundance Analyte. *Anal. Chem.* **2019**, *91*, 15179−15186.

(2) Chen, J.; Wong, J.; Kuo, P.; Yang, D. Synthesis and Characterization of Coumarin-Based Spiropyran Photochromic Colorants. *Org. Lett*. **2008**, *10*, 4823–4826.

(3) Isak, S.; Eyring, E. Fluorescence quantum yield of cresyl violet in methanol and water as a function of concentration. *J. Phys. Chem.* **1992**, *96*, 1738–1742.

(4) Cosco, E.; Caram, J.; Bruns, O.; Franke, D.; Day, R.; Farr, E.; Bawendi, M.; Sletten, E. Flavylium Polymethine Fluorophores for Near- and Shortwave Infrared Imaging. *Angew. Chem. Int. Ed.* **2017**, *56*, 13126−13129.

(5) Yuan, L.; Lin, W.; Yang, Y.; Chen, H. A Unique Class of Near-Infrared Functional Fluorescent Dyes with Carboxylic-Acid-Modulated Fluorescence ON/OFF Switching: Rational Design, Synthesis, Optical Properties, Theoretical Calculations, and Applications for Fluorescence Imaging in Living Animals. *J. Am. Chem. Soc*. **2012**, *134*, 1200–1211.

(6) Mosmann, T. J. Rapid colorimetric assay for cellular growth and survival: application to proliferation and cytotoxicity assays. *Immunol. Methods.* **1983**, *65*, 55–63.
